# Haplotype-resolved chromosome-level genome of hexaploid Jerusalem artichoke provides insights into its origin, evolution, and inulin metabolism

**DOI:** 10.1016/j.xplc.2023.100767

**Published:** 2023-11-17

**Authors:** Sen Wang, Anqi Wang, Rong Chen, Dong Xu, Hengchao Wang, Fan Jiang, Hangwei Liu, Wanqiang Qian, Wei Fan

**Affiliations:** 1 Guangdong Laboratory for Lingnan Modern Agriculture (Shenzhen Branch), Genome Analysis Laboratory of the Ministry of Agriculture and Rural Affairs, Agricultural Genomics Institute at Shenzhen, Chinese Academy of Agricultural Sciences, Shenzhen, Guangdong 518120, China; 2 Guangdong Provincial Key Laboratory for Crop Germplasm Resources Preservation and Utilization, Agro-Biological Gene Research Center, Guangdong Academy of Agricultural Sciences, Guangzhou 510640, China; 3 College of Agronomy, Qingdao Agricultural University, Qingdao 266109, China

**Keywords:** *Helianthus tuberosus*, hexaploid genome, hybridization origin, chromosome rearrangement, inulin metabolism genes

## Abstract

Jerusalem artichoke (*Helianthus tuberosus*) is a global multifunctional crop. It has wide applications in the food, health, feed, and biofuel industries and in ecological protection; it also serves as a germplasm pool for breeding of the global oil crop common sunflower (*Helianthus annuus*). However, biological studies of Jerusalem artichoke have been hindered by a lack of genome sequences, and its high polyploidy and large genome size have posed challenges to genome assembly. Here, we report a 21-Gb chromosome-level assembly of the hexaploid Jerusalem artichoke genome, which comprises 17 homologous groups, each with 6 pseudochromosomes. We found multiple large-scale chromosome rearrangements between Jerusalem artichoke and common sunflower, and our results show that the hexaploid genome of Jerusalem artichoke was formed by a hybridization event between a tetraploid and a diploid *Helianthus* species, followed by chromosome doubling of the hybrid, which occurred approximately 2 million years ago. Moreover, we identified more copies of actively expressed genes involved in inulin metabolism and showed that these genes may still be undergoing loss of function or sub- or neofunctionalization. These genomic resources will promote further biological studies, breeding improvement, and industrial utilization of *Helianthus* crops.

## Teaser

This study reports a 21-Gb chromosome-level genome assembly for the hexaploid Jerusalem artichoke, the identification of multiple large-scale chromosome rearrangements between Jerusalem artichoke and common sunflower, and the observation of more copies of actively expressed genes involved in inulin metabolism in Jerusalem artichoke. It provides evidence supporting that the hexaploid genome of Jerusalem artichoke was formed by hybridization between a tetraploid and a diploid *Helianthus* species.

## Introduction

Jerusalem artichoke (*Helianthus tuberosus*) is a tuber crop in the Asteraceae (Compositae), the largest family of angiosperms, which includes over 25 000 species (Mandel et al., 2019). This crop was first domesticated by native North Americans, grown for its tubers as food and vegetables, and introduced to Europe and then Asia after the discovery of the New World (Yang et al., 2018). Jerusalem artichoke and the global oil crop common sunflower (*Helianthus annuus*) belong to the same genus, and they can be crossed with each other to transfer disease-resistance genes through breeding (Kantar et al., 2018). Currently, the fructan-rich tubers of Jerusalem artichoke are used mainly for industrial production of inulin (fructan with higher polymerization) and fructan oligosaccharides, which are widely used as dietary fiber in the health and fitness industry and as additives or sweeteners in the food and beverage industry (Khuenpet et al., 2017). The tubers can also be used for bioethanol and biodiesel production, and the shoots and leaves serve as nutrition-rich silage for animal feed. Because of its strong stress resistance and high biomass production, Jerusalem artichoke has been widely grown on desertified, saline, and alkaline land to protect and restore ecosystems (Lv et al., 2019).

Jerusalem artichoke is a perennial hexaploid (2n = 6x = 102) species of the genus *Helianthus*, which includes over 50 annual and perennial species ranging from diploids (2n = 2x = 34) to tetraploids (2n = 4x = 68) to hexaploids (Qiu et al., 2019). Previous phylogenetic and evolutionary studies have shown that *Helianthus* is a young genus that arose approximately 3 million years ago (mya) and experienced an ancient whole-genome triplication (WGT1) approximately 45 mya and an ancient whole-genome duplication (WGD2) approximately 29 mya (Rieseberg and Burke, 2008; Badouin et al., 2017). *Helianthus* is a typical genus for reticulate evolution studies, and many species are thought to have originated from hybridization (Owens et al., 2023). Even after several million years of evolution, many *Helianthus* species can still cross with each other naturally, indicating weak reproductive isolation in this genus (Barb et al., 2014). For example, hybridization between Jerusalem artichoke and common sunflower can create fertile neotetraploid hybrids, which can produce viable seeds and transfer stress-tolerance genes to common sunflower cultivars (Kantar et al., 2018). The perennial hexaploid Jerusalem artichoke has been considered an autoallopolyploid originating from the hybridization of perennial *Helianthus* species, and autotetraploid hairy sunflower (*Helianthus hirsutus*) and diploid sawtooth sunflower (*Helianthus grosseserratus*) have been proposed as its likely progenitors (Bock et al., 2014). Until now, when and how this hybridization took place have been unknown, and clear details regarding the origin and evolution of Jerusalem artichoke are still lacking.

Over 15% of angiosperm plants contain fructans as essential compounds for energy metabolism and stress tolerance, and inulin-type fructan is the major energy-storing compound for Asteraceae plants (Valluru and Van Den Ende, 2008). The tuber yield of Jerusalem artichoke is as high as 30–45 tons per hectare, and inulin can account for up to 80% of its dry matter, making it an ideal crop for industrial inulin production (Tanjor et al., 2009). In addition, Jerusalem artichoke has been the model for plant fructan metabolism studies, and the enzymes involved in inulin synthesis were first purified from its tuber tissues (Koops and Jonker, 1994). Inulin is synthesized from sucrose and accumulates in the vacuoles of tuber cells. Its synthesis is catalyzed by two enzymes: 1-sucrose:sucrose fructosyltransferase (1-SST) and 1-fructan:fructan fructosyltransferase (1-FFT) (Vijn and Smeekens, 1999). The hydrolysis of inulin is catalyzed by 1-fructan exohydrolase I (1-FEHI and 1-FEHII) (Vijn and Smeekens, 1999). To date, four genes encoding these four enzymes have been cloned and functionally verified in Jerusalem artichoke: *1-SST*, *1-FFT*, *1-FEHI*, and *1-FEHII* (Van Der Meer et al., 1998; Xu et al., 2015). It should be noted that Jerusalem artichoke is a young hexaploid plant, and its genome may have multiple copies of inulin metabolism genes derived from multiple rounds of whole-genome polyploidization. Many copies of genes involved in inulin metabolism may have been missed in previous studies, and little is known about their fate during the evolutionary history of the hexaploid genome.

In this study, we constructed a 21-Gb chromosome-level hexaploid genome assembly for Jerusalem artichoke, revealed its genome polyploidization history, compared the chromosome synteny between Jerusalem artichoke and common sunflower, and provided genome-wide evidence for the hybridization origin that led to its current hexaploidy. We also identified copies of genes involved in inulin metabolism and revealed their fate during the evolutionary history of this hexaploid genome. The generated reference genome of Jerusalem artichoke will promote biological studies and industrial utilization of this multifunctional crop.

## Results

### Haplotype-resolved chromosome-level assembly of the hexaploid genome

Given the high heterozygosity of Jerusalem artichoke, obtaining a haplotype-resolved assembly that captures all of the variations in gene alleles and homologs, similar to those of autotetraploid potato (*Solanum tuberosum*) (Bao et al., 2022) and alfalfa (*Medicago sativa*) (Chen et al., 2020a), is very important for functional studies and genetic breeding. We selected a local cultivar of Jerusalem artichoke with 2n = 6x = 102 chromosomes for genome sequencing (Supplemental Figure 1) and generated 317.7 Gb of PacBio high-fidelity (HiFi) reads (Supplemental Table 1). The sequencing depth was approximately 15.3-fold coverage of the hexaploid genome size of 20.8 Gb, estimated by K-mer (K = 19) analysis of 550.3 Gb of Illumina short reads (Supplemental Figure 2). The HiFi reads were assembled into 1033 contigs with an N50 of 6.3 Mb and a total assembly size of 21.6 Gb, similar to the estimated genome size (Table 1; Supplemental Table 2). The contig assembly size of 21.6 Gb is just six-fold higher than the genome size of common sunflower (3.6 Gb) (Badouin et al., 2017), and BUSCO (Benchmarking Universal Single-Copy Orthologs) completeness of the contig assembly was as high as 98.4% (eudicot lineage), indicating that we obtained a relatively complete contig assembly for this hexaploid genome.

**Table 1:** Statistics for genome assembly and annotation of *H. tuberosus*.

	Hexaploid genome (6×)		Reference genome (3×)	
	Number	Size	Number	Size
Genome assembly				
Estimated genome size	–	20.8 Gb	–	10.4 Gb
Total assembly size	–	21.6 Gb	–	10.5 Gb
Contig L50/N50 size	1033	6.3 Mb	500	6.5 Mb
Scaffold L50/N50 size	49	201.0 Mb	23	211.5 Mb
Pseudochromosomes	102	20.2 Gb	51	10.5 Gb
BUSCO completeness of assembly	–	98.4%	–	97.2%
LAI	–	18.2	–	17.8
QV value	–	50.7	–	52.4
Mapped reads		99.2%		98.6%
Genome annotation				
TRs	1 983 572	805.1 Mb	9621	376.0 Mb
TEs	16 695 768	20.1 Gb	8 052 723	9.8 Gb
Protein-coding genes	388 053	447.0 Mb	199 842	229.9 Mb
Genes in pseudochromosomes	360 255	420.0 Mb	185 943	216.4 Mb
BUSCO completeness of annotation	–	98.4%	–	96.6%

**Figure 1:**
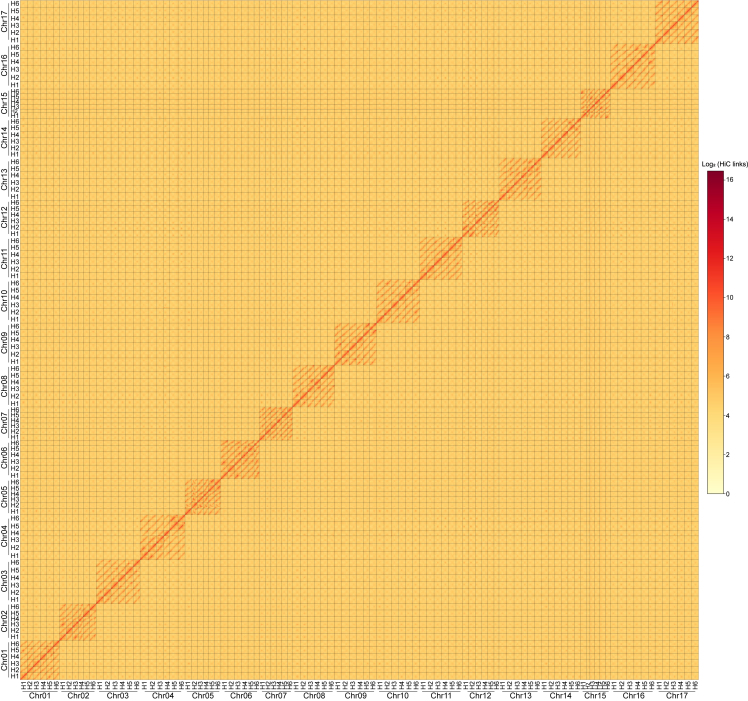
Hexaploid genome-wide Hi-C contact heatmap of *H. tuberosus*. A total of 102 chromosomes were classified into 17 homologous groups; different chromosomes are separated by solid lines. Each bin in the heatmap represents a 3-Mb genomic region, and its color is proportional to the log2-transformed Hi-C links between two 3-Mb bins or within one 3-Mb bin.

To further assemble the contigs into haplotype-resolved chromosome-level scaffolds, we used genomic Hi-C (high-throughput chromosome conformation capture) sequencing to capture the spatial organization information of the 102 chromosomes of Jerusalem artichoke. Using ∼1.0 G of Hi-C read pairs (Supplemental Table 3), we performed two rounds of the divide-and-conquer strategy and a small amount of manual curation (Supplemental Figures 3–6) in JuiceBox (Durand et al., 2016) to obtain the final 102 chromosome-level haplotype-resolved scaffolds with an N50 size of 201.0 Mb (Figure 1 and Supplemental Table 2). The total length of the 102 pseudochromosomes was 20.2 Gb, accounting for 93.5% of the total genome size. In the hexaploid-genome-wide Hi-C contact heatmap, 102 pseudochromosomes clearly formed 17 groups, each with exactly 6 pseudochromosomes, and most Hi-C signals were concentrated within 1 pseudochromosome or among the 6 pseudochromosomes of the same group, suggesting that the 6 pseudochromosomes in each group are homologous chromosomes. Sequence alignment of the 102 pseudochromosomes of Jerusalem artichoke to the 17 chromosomes of common sunflower also showed that the 6 pseudochromosomes of each group were homologous, and we named the 17 groups on the basis of their syntenic relationships to the corresponding 17 common sunflower chromosomes. Although some chromosome fragments were missing for hexaploid Jerusalem artichoke, we successfully constructed relatively complete and haplotype-resolved chromosome-level scaffolds for most groups of homologous chromosomes. The mapping rate of the short-read data was 99.2%, the long terminal repeat (LTR) assembly index (LAI) of ∼18 met the standards for a reference genome, and the quality value (QV) calculated by Merqury was greater than 50, all suggesting the high quality of the hexaploid assembly (Table 1). The *de novo* assembly approach used for hexaploid Jerusalem artichoke provides a cost-effective solution for chromosome-level assembly of high-ploidy genomes, especially when the ancestors are unknown or unavailable.

We comprehensively annotated the hexaploid genome of Jerusalem artichoke to identify tandem repeats (TRs), transposable elements (TEs), and protein-coding and noncoding RNA genes. TRs, with a total length of 805.1 Mb, accounted for 3.7% of the whole genome, and TEs, with a total length of 20.1 Gb, accounted for up to 93% of the whole genome. The most common TEs were *Gypsy* (45.7%) and *Copia* (12.1%) LTR retrotransposons (Supplemental Tables 4 and 5), similar to those reported in the common sunflower genome (Badouin et al., 2017). Combining *ab initio* prediction, transcript alignment of 281 542 full-length mRNAs and 28 RNA sequencing (RNA-seq) datasets from different tissues (Supplemental Tables 6 and 7), and protein homologs from 10 Asteroideae species (Table 1 and Supplemental Table 8), we annotated 388 053 protein-coding genes in the hexaploid genome of Jerusalem artichoke (Supplemental Table 9), which was approximately six times the gene number of common sunflower (Badouin et al., 2017). Gene functional annotation predicted the functions of 365 080 protein-coding genes (94.00%) based on at least one hit from the NCBI-NR, Kyoto Encyclopedia of Genes and Genomes (KEGG), and InterPro databases (Supplemental Table 10). Overall, the gene number and density distribution were similar among the 17 groups of homologous chromosomes, and each chromosome had an average of 3532 genes (Supplemental Tables 11 and 12). BUSCO completeness of the protein-coding genes was 98.4% (eudicot lineage), the same as that of the genome assembly. We also identified 11 951 tRNA genes and 37 336 rRNA genes (Supplemental Table 13). Thus, the reference genome of hexaploid Jerusalem artichoke and its comprehensive annotations lay the foundation for further biological studies of this crop.

### Origin of *H. tuberosus* from hybridization between a tetraploid and a diploid *Helianthus* species

**Figure 2:**
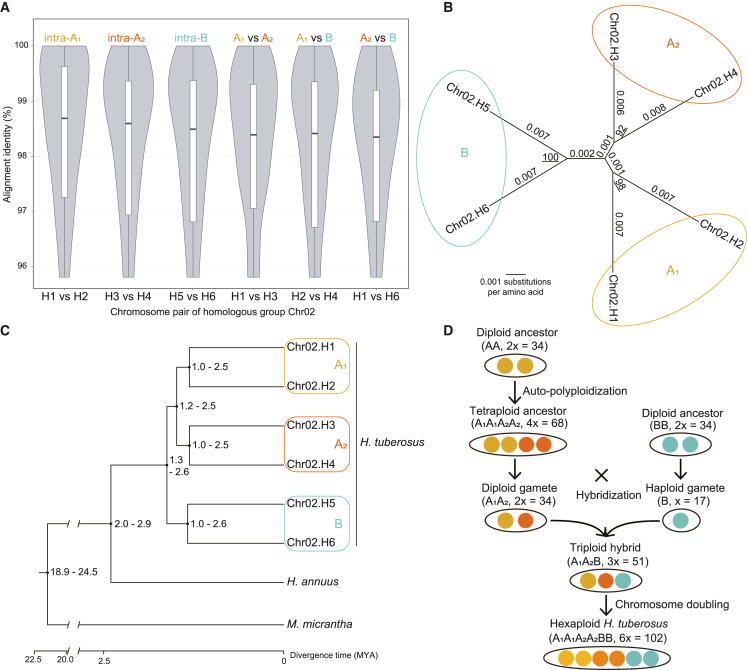
Hybridization origin of autoallohexaploid *H. tuberosus*. **(A)** Violin plot and boxplot of alignment identities of single-copy genes between homologous chromosomes from the same subgenome (intra-A_1_, A_2_, or B) and different subgenomes (A_1_ vs. A_2_, A_1_ vs. B, or A_2_ vs. B). **(B)** Unrooted phylogenetic tree of six homologous chromosomes constructed from the trimmed CMSA of single-copy genes. Branch lengths indicate the number of substitutions per amino acid, and underlined integers refer to bootstrap support values of the corresponding nodes. **(C)** Divergence time tree of six homologous chromosomes of *H. tuberosus*, *H. annuus*, and *M. micrantha*. **(A–C)** The homologous group chr02 was selected as a representative of the 17 groups of homologous chromosomes. Homologous synaptic pairs of chromosomes (heterozygous pairs) in the different subgenomes A_1_, A_2_, and B are marked by ellipses or rounded rectangles in different colors. **(D)** Illustration of the inferred history from the diploid ancestors (AA, BB) and tetraploid ancestor (A_1_A_1_A_2_A_2_) to the hybridization and chromosome-doubling events that generated hexaploid *H. tuberosus* (A_1_A_1_A_2_A_2_BB).

For a recently arisen hexaploid with high heterozygosity such as Jerusalem artichoke, it is of great interest to determine the relationships among homologous chromosomes and infer the genome structure and polyploid origin. The six chromosomes of a given homologous group can be divided into three heterozygous pairs that undergo synapsis during meiosis. If the divergence among heterozygous pairs is clearly larger than that within heterozygous pairs, then the hexaploid genome has a subgenome structure. Using the haplotype-resolved chromosome-level hexaploid genome assembly of Jerusalem artichoke, we compared the sequence divergence among the 6 chromosomes for each of the 17 homologous groups and found that the sequence differences between any 2 chromosomes from the same homologous group were highly similar, with a divergence of approximately 2%. Thus, it was difficult to discriminate synaptic chromosome pairs or subgenomes (Supplemental Figure 7). We thought that the chromosome-level sequence divergence analysis may have contained too much noise caused by alignment errors. We therefore identified and aligned single-copy genes for each homologous group of 6 chromosomes, and the identity of genes from chromosomes of the same subgenome clearly revealed subgenome structure: the average identity of genes from the same subgenome (97.7%, i.e., ∼2.3% heterozygous rate) was slightly higher than the average identity of genes from different subgenomes (97.3%) (Table 2), as exemplified by the homologous group of chromosome 02 (chr02) (Figure 2A and Supplemental Figure 8). By comparison, the divergence rate between Jerusalem artichoke and common sunflower was about 5% when calculated with a similar method. These findings provide a genomic basis for the previously observed chromosome synapsis behavior in which the 102 chromosomes of hexaploid Jerusalem artichoke frequently form 51 bivalents during meiosis (Atlagić et al., 1993).

**Table 2:** Comparisons of homologous chromosomes of *H. tuberosus*.

	Length (Mb)	Gene number	Single-copy genes	Genes used for phylogeny^a^	Phylogenetic tree topology^b^	Intra-A_1_/A_2_/B identity^c^	Inter-A_1_/A_2_/B identity^d^
Chr01	173–212	3212–3716	607	227	([H_1_,H_2_],[H_3_,H_4_],[H_5_,H_6_])	97.16%	97.04%
Chr02	130–200	2374–3198	542	542	([H_1_,H_2_], [H_3_,H_4_], [H_5_,H_6_])	97.24%	96.99%
Chr03	206–230	3663–4109	864	864	([H_1_,H_2_], [H_3_,H_4_], [H_5_,H_6_])	97.19%	96.98%
Chr04	166–255	2615–4539	383	383	([H_1_,H_2_], [H_3_,H_4_], [H_5_,H_6_])	98.67%	97.16%
Chr05	144–193	2628–3140	548	240	([H_1_,H_2_], [H_3_,H_4_], [H_5_,H_6_])	97.69%	97.32%
Chr06	159–209	3230–3917	691	691	([H_1_,H_2_], [H_3_,H_4_], [H_5_,H_6_])	98.00%	97.14%
Chr07	155–173	2309–2795	509	509	([H_1_,H_2_], [H_3_,H_4_], [H_5_,H_6_])	98.02%	97.81%
Chr08	193–229	3290–3858	735	735	([H_1_,H_2_], [H_3_,H_4_], [H_5_,H_6_])	97.81%	97.42%
Chr09	193–226	3531–4336	748	748	([H_1_,H_2_], [H_3_,H_4_], [H_5_,H_6_])	97.90%	97.41%
Chr10	198–228	3443–3997	760	760	([H_1_,H_2_], [H_3_,H_4_], [H_5_,H_6_])	97.46%	97.24%
Chr11	188–223	3286–3942	678	678	([H_1_,H_2_], [H_3_,H_4_], [H_5_,H_6_])	97.43%	97.22%
Chr12	148–201	2599–3657	495	495	([H_1_,H_2_], [H_3_,H_4_], [H_5_,H_6_])	98.88%	97.67%
Chr13	190–227	3156–3989	421	421	([H_1_,H_2_], [H_3_,H_4_], [H_5_,H_6_])	97.52%	97.09%
Chr14	184–205	3702–4104	867	867	([H_1_,H_2_], [H_3_,H_4_], [H_5_,H_6_])	97.84%	97.31%
Chr15	99–166	1998–3079	438	165	([H_1_,H_2_], [H_3_,H_4_], [H_5_,H_6_])	97.55%	97.13%
Chr16	205–246	3419–4461	716	716	([H_1_,H_2_], [H_3_,H_4_], [H_5_,H_6_])	97.60%	97.26%
Chr17	199–235	3454–4215	620	620	([H_1_,H_2_], [H_3_,H_4_], [H_5_,H_6_])	97.56%	97.42%

a Number of single-copy genes used to infer the phylogenetic tree of six homologous chromosomes in each group. For chr01, chr05, and chr15, only single-copy genes with alignment identity of 92%–100% were used.

b A phylogenetic tree was constructed from the CMSA of single-copy genes (gap regions were trimmed out).

c Average alignment identity of single-copy genes between two homologous chromosomes of the same subgenome: A_1_ (H_1_ and H_2_), A_2_ (H_3_ and H_4_), or B (H_5_ and H_6_).

d Average alignment identity of single-copy genes between two homologous chromosomes of different subgenomes: A_1_ and A_2_, A_1_ and B, or A_2_ and B.

The origin and genome formula of hexaploid Jerusalem artichoke have not previously been demonstrated through a genome-wide comparison. We reconstructed the phylogeny of homologous chromosomes using a concatenated multiple sequence alignment (MSA) of single-copy genes for each of the 17 homologous groups, and the topology and branch lengths of most unrooted phylogenetic trees indicated that hexaploid Jerusalem artichoke was an autoallopolyploid with 3 subgenomes, A_1_, A_2_, and B (Table 2; Figure 2B and Supplemental Figure 9). We inferred that the direct ancestors of Jerusalem artichoke may have been an autotetraploid and a diploid *Helianthus* species and that the hybridization of these two ancestors and the subsequent chromosome doubling led to speciation of Jerusalem artichoke. Similarly, previous studies of the origin of Jerusalem artichoke based on rDNA analysis and cytologic investigation of meiosis in a hybrid between Jerusalem artichoke and common sunflower also suggested that the hexaploid genome structure was A_1_A_2_B (Kostoff, 1939) and that the ancestors may have been autotetraploid hairy sunflower and diploid sawtooth sunflower (Bock et al., 2014). Here, we provide more convincing genome-scale evidence for the hybridization origin of autoallohexaploid Jerusalem artichoke.

Treating each homologous chromosome as a virtual species, we estimated the divergence time of homologous chromosomes for each group and two outgroups, common sunflower and *Mikania micrantha* (Supplemental Figure 10). Taking chr02 as an example, the estimated divergence time between 6 chromosomes of Jerusalem artichoke and common sunflower was 2.0–2.9 mya; interspecies hybridization and chromosome doubling occurred 1.0–2.6 mya and led to the rise of hexaploid Jerusalem artichoke (Figure 2C). On the basis of the estimated time tree, we inferred the history from the diploid ancestors to hexaploid Jerusalem artichoke as follows. Two perennial diploids (AA and BB) arose earlier than 2 mya from the *Helianthus* ancestor, and fusion of unreduced gametes or chromosome doubling of somatic cells occurred in the diploid A genome donor and produced a perennial tetraploid (A_1_A_1_A_2_A_2_). Shortly after that, its reduced diploid gamete (A_1_A_2_) fused with the haploid gamete (B) and generated a triploid hybrid (A_1_A_2_B), and chromosome doubling of this hybrid produced the original hexaploid (A_1_A_1_A_2_A_2_BB) Jerusalem artichoke (Figure 2D). After millions of years of mutation, evolution, vegetative reproduction, and domestication, the original hexaploid has become the extant highly heterozygous hexaploid Jerusalem artichoke.

**Figure 3:**
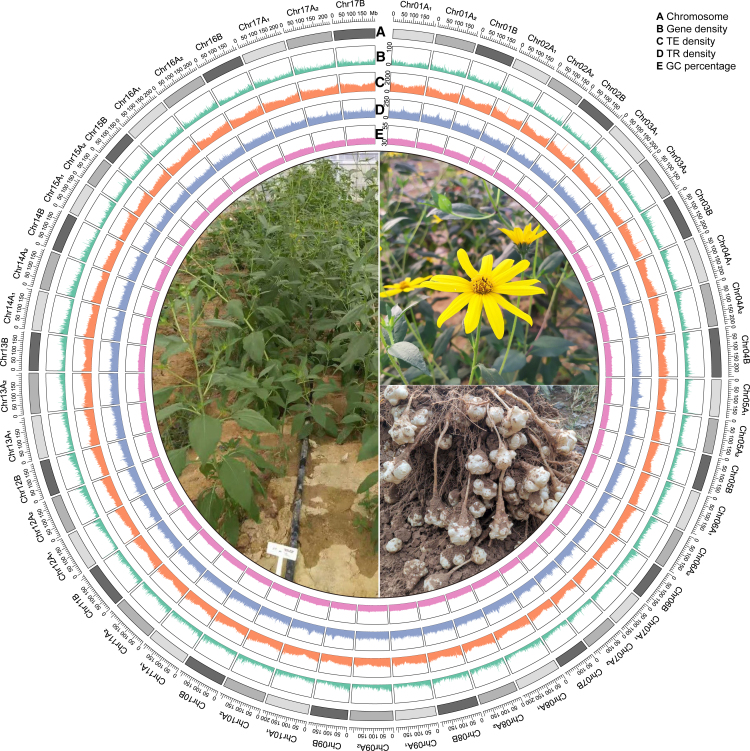
Circos plot of genome annotations for the three subgenomes of *H. tuberosus*. The 5 circular tracks from outside to inside show (A) chromosomes and lengths, (B) gene density, (C) transposable element (TE) density, (D) tandem repeat (TR) density, and (E) GC percentage. Feature density and GC percentage were calculated in sliding 1-Mb windows. Photos of *H. tuberosus* plants, flowers, and tubers are presented in the center.

With the phylogenetic topology for each homologous group, we were able to divide the six chromosomes (H_1_–H_6_) of the same homologous group into three heterozygous pairs (A_1_ [H_1_ and H_2_], A_2_ [H_3_ and H_4_], and B [H_5_ and H_6_]), with each pair derived from a subgenome. By selecting 1 chromosome from each heterozygous pair, we obtained 3 reference chromosomes (H_1_, H_3_, and H_5_) for each of the 17 homologous groups, which together constituted the reference genome of Jerusalem artichoke. The 199 842 genes residing in these 51 reference chromosomes were taken as the reference gene set of Jerusalem artichoke (Figure 3). The BUSCO completeness ratio (97.2%), read mapping rate (98.6%), and LTR LAI (17.8) of the triploid reference genome were a little lower than those of the hexaploid assembly, but the Merqury QV (52.4) was somewhat higher, suggesting that the triploid reference genome was still a high-quality genome assembly (Table 1). The statistics and assessment for each ploidy and each chromosome are shown in Supplemental Tables 11 and 12.

### Large-scale chromosome rearrangements have shaped the *Helianthus* genome

**Figure 4:**
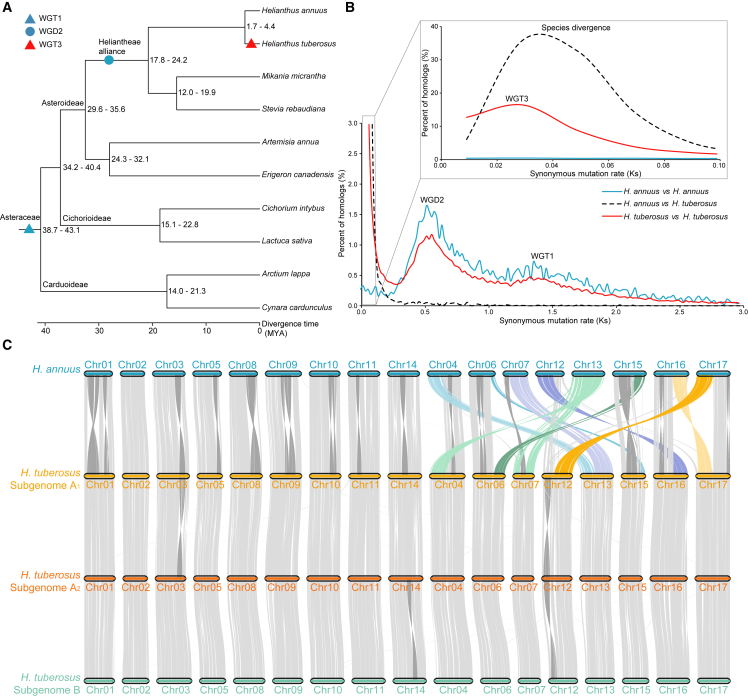
Genome evolution of *H. tuberosus*. **(A)** Species divergence time tree of 10 Asteraceae species constructed from the CMSA of 1136 conserved ortholog groups (OGs), with the ancient whole-genome triplication (WGT1; blue triangle) marked for the ancestor of the Asteraceae, the ancient whole-genome duplication (WGD2; blue circle) marked for the ancestor of the Heliantheae alliance, and the recent WGT3 (orange circle) marked for *H. tuberosus* (highlighted in red). **(B)** Distribution curve of the synonymous mutation rate (Ks) of syntenic gene pairs within *H. tuberosus*, within *H. annuus*, and between the two species; WGT1, WGD2, WGT3, and species divergence are marked at the corresponding Ks peaks. The reference gene set from haploid *H. annuus* and the reference gene sets from the three subgenomes of *H. tuberosus* were used in the Ks distribution analysis. **(C)** Macrosyntenic blocks between 17 chromosomes of *H. annuus* and 51 chromosomes from 3 subgenomes of *H. tuberosus*. Intra-chromosomal inversions are highlighted in gray, and inter-chromosomal translocations are highlighted in different colors.

To determine the phylogenetic history of Jerusalem artichoke, we selected nine representative species of Asterids II (two Carduoideae, two Cichorioideae, and five Asteroideae species) and one outgroup, *Coffea canephora* (Denoeud et al., 2014), as a representative of Asterids I, to identify OGs (ortholog groups) (Supplemental Table 14). The species phylogeny and the time tree constructed with the concatenated MSA of 1109 conserved OGs showed that Jerusalem artichoke and common sunflower were in the same clade and diverged from each other 1.7–4.4 mya (Figure 4A and Supplemental Figure 11).

Multiple genome polyploidization events are reported to have shaped the evolution of Asteraceae species (Zhang et al., 2021). We analyzed the synonymous mutation rate (Ks) of syntenic genes within and between the hexaploid Jerusalem artichoke genome and the common sunflower genome and found that both species experienced the WGT1 (Ks peak at ∼1.4) of the Asteraceae ancestor and the later WGD2 (Ks peak at ∼0.55) of the ancestor of the Heliantheae alliance, consistent with a previous report on the common sunflower genome (Badouin et al., 2017). In addition, Jerusalem artichoke also experienced a recent WGT3 (Ks peak at ∼0.03) event after its divergence (Ks peak at ∼0.035) from common sunflower (Figure 4B). The intraspecies synteny dot plot of Jerusalem artichoke and the interspecies synteny dot plot between Jerusalem artichoke and common sunflower (Supplemental Figures 12 and 13) also support this WGT3 event.

The six chromosomes of each homologous group of hexaploid Jerusalem artichoke were largely syntenic to each other, except for several intra-chromosome inversions that occurred in some chromosomes (Figure 4C; Supplemental Figures 12 and 13), which may affect synapsis during meiosis and explain why Jerusalem artichoke rarely produces seeds. However, the chromosome-level syntenic relationships between Jerusalem artichoke and common sunflower were far from one-to-one, indicating that large-scale chromosome rearrangements have taken place in the ancestors of the Jerusalem artichoke or common sunflower lineages (Figure 4C). We compared the 17 H1 chromosomes representing subgenome A_1_ with the 17 chromosomes of common sunflower to illustrate the chromosome inversions and translocations between these two species. There were 9 pairs of 1-to-1 chromosomes between Jerusalem artichoke and common sunflower: chr01, chr02, chr03, chr05, chr08, chr09, chr10, chr11, and chr14. The remaining 8 pairs of chromosomes showed large-scale translocations between Jerusalem artichoke and common sunflower: chr04 to chr04 and chr13, chr06 to chr06 and chr15, chr07 to chr07 and chr13, chr12 to chr12 and chr16, chr13 to chr07 and chr04, chr15 to chr15 and chr06, chr16 to chr16 and chr17, and chr17 to chr17 and chr12. In total, at least 11 chromosome breaks and 13 fusions were needed to generate the extant karyotypes of Jerusalem artichoke and common sunflower from their latest common ancestor.

In a previous study, phylogenetic analysis of the genetic maps of several annual *Helianthus* plants, including *H. annuus*, *Helianthus petiolaris*, *Helianthus argophyllus*, and *Helianthus niveus*, revealed that only 8 of the 17 chromosomes of all these *Helianthus* plants were involved in inter-chromosomal translocations, and the other 9 chromosomes had only intra-chromosomal inversions (Ostevik et al., 2020). In our findings, the eight chromosomes of common sunflower with translocations were exactly consistent with those reported previously on the basis of genetic maps (Ostevik et al., 2020), suggesting that translocation events were nonrandom among all chromosomes in all *Helianthus* plants. Our findings here provide further examples of exceptional large-scale chromosome rearrangements in *Helianthus* species. These non-random large-scale genome rearrangements may change the gene structure and regulation network, extend the genomic regions that are protected from introgression, promote the recombination of non-allelic genes, influence reproductive isolation and speciation, and thus contribute to rapid diversification and adaptation of all *Helianthus* plants. Information on these chromosome rearrangements may also be beneficial for the breeding process.

### Multiple actively expressed copies of inulin metabolism genes are in the early stage of evolution in Jerusalem artichoke

**Figure 5:**
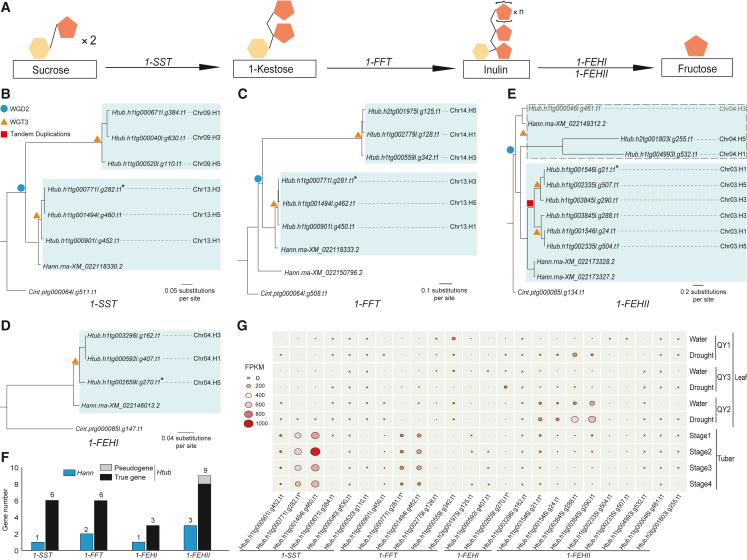
Evolutionary fates and expression patterns of inulin metabolism genes in the three subgenomes of *H. tuberosus*. **(A)** The roles of *1-SST*, *1-FFT*, and *1-FEH* genes in inulin synthesis and degradation. **(B–E)** Phylogenetic trees of six *1-SST* genes, six *1-FFT* genes, three *1-FEHI* genes, and nine *1-FEHII* genes from *H. tuberosus* (*Htub*), with the homologous genes from *H. annuus* (*Hann*) and *C. intybus* (*Cint*) as outgroups. The locations for *Htub* genes are shown on the right side of the gene ID. WGD2 (blue circle) and the recent WGT3 (orange triangle) are marked at the *Helianthus* ancestor and *Htub*, respectively. The tandem duplications of *FEHII* are marked by red squares, and the two WGD2-derived clades of inulin metabolism genes are highlighted in light green. Gene names in black are true genes with complete structures, and gene names in gray are pseudogenes with internal stop codons, frameshifts, or truncated exons. **(F)** Copy numbers of inulin metabolism genes in *Htub* and *Hann*. **(G)** Expression heatmap of inulin metabolism genes in tuber tissues of four different growth stages and leaf tissues of three cultivars under watered or drought conditions. The size and color of the circle are proportional to the fragments per kilobase per million reads.

Jerusalem artichoke is a model species for studies of plant fructan metabolism and a major crop used for industrial inulin production. Its three subgenomes may have multiple copies of genes involved in inulin synthesis and hydrolysis (Figure 5A), which are of great interest and significance. Using the sequences of the three subgenomes, we comprehensively identified all copies of inulin metabolism genes in Jerusalem artichoke, which have experienced both ancient and recent genome polyploidization events (Supplemental Figures 14–16). In total, there were six *1-SST* and six *1-FFT* genes derived from WGD2 and WGT3 and one clade of three *1-SST* and three *1-FFT* genes located near each other on the homologous chr13 chromosomes (Figure 5B and 5C), as has also been reported for the genomes of chicory, endive, great burdock, and yacon (Fan et al., 2022). There were three *1-FEHI* genes, which were located on the homologous chr04 chromosomes. These *1-FEHI* genes were derived from WGT3 based on one clade of WGD2, and the other clade of WGD2 was missing (Figure 5D). Nine *1-FEHII* genes, including eight true genes and one pseudogene, were located on the homologous chr03 chromosomes (six genes) and chr04 chromosomes (three genes). Note that each chromosome of homologous group chr03 had two tandemly located *1-FEHII* genes. These *1-FEHII* genes were derived from WGD2, tandem duplication of one clade of WGD2, and WGT3 events (Figure 5E). Compared with genes of an ancient polyploid species that has undergone long-term evolution, the multiple copies of inulin metabolism genes in Jerusalem artichoke may still be undergoing loss of function or sub- or neofunctionalization.

The three subgenomes of Jerusalem artichoke had six, six, three, and eight true gene copies of *1-SST*, *1-FFT*, *1-FEHI*, and *1-FEHII*, respectively (Figure 5F). In addition, one pseudogene of *1-FEHII* with an internal frameshift was identified. RNA-seq analysis showed that most of these inulin metabolism genes were actively transcribed in tuber or leaf tissues of Jerusalem artichoke (Figure 5G). Across the growth stages of tuber development, the newly identified two *1-SST* (*Htub.h1tg000901l.g452.t1* and *Htub.h1tg001494l.g460.t1*) and two *1-FFT* (*Htub.h1tg000901l.g450.t1* and *Htub.h1tg001494l.g462.t1*) genes and the previously cloned *1-SST* (*Htub.h1tg000771l.g282.t1*) and *1-FFT* (*Htub.h1tg000771l.g281.t1*) genes showed similar expression patterns, which may contribute to the process of inulin accumulation in tubers. The remaining three *1-SST* genes showed very low expression and may have lost their functions. The remaining three *1-FFT* genes were expressed mainly in leaf tissue, possibly as a result of subfunctionalization. The newly identified *1-FEHI* (*Htub.h1tg000592l.g407.t1*) and five *1-FEHII* genes (*Htub.h1tg001546l.g24.t1*, *Htub.h1tg003845l.g288.t1*, *Htub.h1tg003845l.g290.t1*, *Htub.h1tg002335l.g507.t1*, and *Htub.h1tg002335l.g504.t1*) and the previously cloned *1-FEHI* (*Htub.h1tg002659l.g270.t1*) and *1-FEHII* (*Htub.h1tg001546l.g21.t1*) genes were expressed mainly in leaf tissues, and their expression was clearly induced under drought-stress conditions. This was consistent with previous findings that inulin hydrolysis catalyzed by 1-FEH can increase solute concentrations and help to sustain osmotic balance, enabling plants to adapt to drought environments (Ende, 2013). The remaining one *1-FEHI* gene and three *1-FEHII* genes showed very low expression and may have lost their functions. Thus, the newly identified, actively expressed genes involved in inulin metabolism in Jerusalem artichoke deserve further study and utilization in the future.

Inulin metabolism is important for energy storage and stress tolerance in Jerusalem artichoke and should therefore be finely regulated to cope with growth, development, and environmental changes. In Asteraceae plants, several MYB transcription factors (TFs) have been reported to regulate the expression of inulin metabolism genes in chicory (Wei et al., 2017a, 2017b). Using transcriptome profiles of Jerusalem artichoke at various growth stages and under various stress conditions (Supplemental Table 15), we performed WGCNA (weighted gene co-expression network analysis) to identify potential TFs that may regulate the expression of inulin metabolism genes (Supplemental Figure 17A). We found that the expression of four *bHLH*, three *YABBY*, four *ERF*, and one *WRKY* TF gene was highly correlated with that of *1-FFT* genes in tuber tissues at different growth stages and in leaf tissues under different drought stresses (Supplemental Figure 17B). *ERF* and *WRKY* TFs have been reported to participate in plant drought responses (Valluru, 2015; Zhao et al., 2020), and the binding motifs of these TFs were present in the promoter regions of the *1-FFT* genes (Supplemental Table 16). Therefore, identification of inulin metabolism genes and their potentially associated TFs will lead to a more complete understanding of inulin metabolism in Jerusalem artichoke.

## Discussion

For polyploid species, haplotype-resolved assemblies are necessary to capture the allelic variations in homologous genes, which are very important for studies of gene expression regulation, genomic imprinting, and heterosis utilization (Van De Peer et al., 2021). Until now, obtaining a haplotype-resolved chromosome-level assembly has been challenging for polyploid species, especially autopolyploids, because a high content of repetitive elements, highly similar homologous sequences, a large genome size, and high heterozygosity are frequently present in the same genome, creating intractable difficulties for haplotype phasing and chromosome scaffolding (Kong et al., 2023). Although the chromosome-level assembly of diploid genomes has become routine, only a few autopolyploid plants, such as autotetraploid potato (Bao et al., 2022), alfalfa (Chen et al., 2020b), and sugarcane (Zhang et al., 2022), have haplotype-phased chromosome-level genome assemblies. These assemblies rely on a genetic map constructed from selfing populations to phase contigs into different haplotypes or on a reference genome of a closely related diploid species without chromosome rearrangements to group contigs into different homologous groups; uniquely mapped Hi-C read pairs are then used to anchor the contigs into pseudochromosomes (ALLHiC prune, partition, and optimize steps). In the present study, it would have been difficult to construct a genetic map for autoallohexaploid Jerusalem artichoke because it is mainly propagated through rhizomes and has a very weak ability to produce fertile seeds. Moreover, although there is a chromosome-level genome of the close diploid relative common sunflower, large-scale chromosome translocations between common sunflower and Jerusalem artichoke make it difficult to use the common sunflower reference genome to separate Jerusalem artichoke contigs into correct homologous groups.

Because ALLHiC is a Hi-C scaffolding tool designed for haplotype-resolved assembly of polyploid genomes (Zhang et al., 2019), we expected that it would be able to produce chromosome-level scaffolds of Jerusalem artichoke. Unexpectedly, the uniquely mapped Hi-C pairs from the ALLHiC prune step were too sparse to confidently anchor the contigs of one homologous group into haplotype-resolved chromosome-level scaffolds. To solve this problem, we retained both uniquely mapped and multi-mapped Hi-C read pairs for each homologous group of contigs, and only the best hit was kept for each Hi-C read. Most of the scaffolding work relied on software, and a few mis-joins were manually corrected in JuiceBox. Because of the large-scale translocations, one round of scaffolding work did not generate a satisfactory haplotype-resolved assembly for all chromosomes. Luckily, the second round of scaffolding work, which took advantage of the assembly results from the first round, greatly improved the completeness of the final scaffold assembly, especially for homologous groups with large-scale translocations. Note that the assemblies of a few chromosomes are still somewhat shorter than those of other chromosomes in the same homologous group owing to the absence of some highly similar DNA fragments that could not be successfully resolved in the contig or scaffold assembly processes with current technologies. This near-chromosome-level reference genome assembly is a great milestone on the route to a final telomere-to-telomere assembly for Jerusalem artichoke. In addition, the approaches used here provide a cost-effective solution for genome assembly of polyploid plants, which account for over 35% of angiosperm plants (Wu et al., 2020), especially polyploids without known or available ancestors, and will therefore promote the study and utilization of polyploid crops.

The genus *Helianthus* has been an ideal model for studies of hybridization speciation and reticulate evolution (Owens et al., 2023). On the basis of a phylogenetic analysis of homologous chromosomes, we inferred that Jerusalem artichoke originated from hybridization between a tetraploid and a diploid *Helianthus* species approximately 2 mya. Although previous studies based on rDNA analysis and cytologic investigation of meiosis suggested a similar hypothesis, our genome-scale evidence is much more convincing, and we were able to estimate the time of hybridization. A limitation of our analysis is that we cannot infer the ancestor of Jerusalem artichoke owing to a lack of genomic evidence from its parental species. When the genomes of hairy sunflower and sawtooth sunflower are sequenced, the parentage of Jerusalem artichoke can be tested thoroughly. In addition, the large-scale chromosome rearrangements between perennial Jerusalem artichoke and annual common sunflower observed here and the exceptional chromosome rearrangements in annual *Helianthus* species reported previously (Ostevik et al., 2020) both suggest the important role of genome rearrangement in *Helianthus* evolution. Genomic resources for hexaploid Jerusalem artichoke will promote more in-depth studies on the speciation and evolution of *Helianthus* plants. Genome analysis can be an effective approach for elucidating the origin and evolutionary history of polyploid plants, even when their ancestors are unknown or unavailable.

Jerusalem artichoke is a model for studies of plant fructan metabolism and a major crop for industrial inulin production (Vijn and Smeekens, 1999; Tanjor et al., 2009). The enzymes that catalyze inulin synthesis and hydrolysis, 1-SST, 1-FFT, 1-FEHI, and 1-FEHII, were first purified from its tuber tissues (Koops and Jonker, 1994). Notably, the previously cloned genes encoding these enzymes are much fewer in number than expected for hexaploid Jerusalem artichoke (Van Der Meer et al., 1998; Xu et al., 2015), perhaps because of the limitations of PCR cloning. Here, we used the hexaploid genome to identify all copies of inulin metabolism genes, clarify their evolutionary fates during multiple rounds of genome polyploidization, and identify actively expressed gene copies involved in inulin metabolism and their potential associated TFs. These results generate a more complete understanding of inulin metabolism in Jerusalem artichoke and provide more gene resources for inulin research and industrial production. Jerusalem artichoke also has strong resistance to drought, freezing, alkaline, and salt stress and has been widely used for animal feed, bioenergy production, and ecological conservation (Lv et al., 2019). The near-chromosome-level reference genome generated here will promote gene mining, breeding improvement, and industrial utilization of this multifunctional crop.

## Methods

### Plant materials and genome sequencing

A single plant from a local cultivar of Jerusalem artichoke widely grown in northwestern China was selected for karyotype analysis and genome sequencing. The fresh root tips of 4-week-old seedlings were sampled for karyotype analysis by fluorescence staining in order to verify the ploidy and chromosome number of the sequenced cultivar. Fresh young leaves were used for genomic DNA extraction with the Hi-DNAsecure Plant Kit (Tiangen) according to the provided protocol. For the genome survey, high-quality DNA was ultrasonically sheared into 350-bp fragments, prepared into sequencing libraries using the TruSeq DNA Library Prep Kit (Illumina), and sequenced in paired-end 150-bp (PE150) mode on the Illumina NovaSeq 6000 platform. For contig assembly, DNA samples were mechanically sheared into 15-kb fragments using g-TUBEs (Covaris), prepared into SMRT dumbbell libraries using the PacBio SMRTbell Express Template Prep Kit 2.0, and sequenced in HiFi mode on the PacBio Sequel II platform. To assist with chromosome-level scaffold assembly, fresh young leaves of 4-week-old seedlings were used for Hi-C sequencing. Nuclear DNA was cross-linked by soaking leaf tissues in formaldehyde solution. The cross-linked genomic DNA was extracted using the Hi-DNAsecure Plant Kit and digested by endonuclease HindIII. The digested DNA was repaired, ligated to circular fragments, sheared into 350-bp inserts, converted to a short-read sequencing library using the TruSeq DNA Library Prep Kit, and sequenced on the Illumina NovaSeq 6000 platform in PE150 mode.

### Transcriptome sequencing

To obtain full-length transcripts for gene annotation, rhizome, tuber, stem, and leaf tissues of 4-week-old Jerusalem artichoke seedlings and flower and tuber samples of adult plants were sampled for total RNA extraction using the RNeasy Plant Mini Kit (QIAGEN) according to the provided protocols. The mRNA molecules in the quality-checked RNA samples were reverse transcribed to cDNA using the NEBNext Single Cell/Low Input cDNA Synthesis & Amplification Module and the PacBio Iso-Seq Express Oligo Kit. The cDNA fragments (500–6000 bp) were prepared into isoform sequencing libraries using the PacBio SMRTbell Express Template Prep Kit 2.0 and sequenced in HiFi mode on the PacBio Sequel II platform.

For expression profiling of all genes during tuber development, tuber tissues of 13-, 15-, 17-, and 19-week-old Jerusalem artichoke plants were sampled for total RNA extraction and quality checking as described above. The mRNA molecules were sheared into short fragments of 350 bp, converted into sequencing libraries using the VAHTS mRNA-Seq V3 Library Prep Kit (TaKaRa), and sequenced on the Illumina NovaSeq 6000 platform in PE150 mode.

### Assembly of haplotype-resolved pseudochromosomes

To obtain a complete contig assembly for Jerusalem artichoke, we first estimated its hexaploid genome size by K-mer analysis of Illumina short reads using GCE v.1.02 (Binghang et al., 2013). We then used a haplotype-resolved assembly strategy to integrate the genomic HiFi reads with Hi-C data to produce haplotype-resolved contigs using HiFiasm v.0.16.1 (Cheng et al., 2021) with the parameters “-u -l 0 --h1 --h2” to recover inter-haplotype variations and reduce potential mis-join errors as much as possible. The hap1 and hap2 contigs generated by HiFiasm were combined to constitute the whole sequences of the hexaploid genome, and the total assembly size was close to the estimated genome size of 21 Gb. The quality of the contig assembly was assessed by the completeness of 2326 conserved genes of the eudicot lineage using BUSCO v.5.0 (Manni et al., 2021).

We performed two rounds of the split-and-conquer strategy to assemble the contigs of the hexaploid genome into chromosome-level scaffolds. Each round included four major steps. First, HiC-Pro v.3.1.0 (Servant et al., 2015) was used to map the genomic Hi-C reads to the hexaploid genome contigs and process the mappings to obtain valid Hi-C pairs, and only one best hit was retained for multi-mapped reads. Second, we aligned all contigs of Jerusalem artichoke to the 17 chromosomes of common sunflower using Minimap2 v.2.20 (Li, 2018), identified the allelic contigs that had similar alignment length and overlapping positions, and separated all contigs into 17 groups. Third, we removed the Hi-C valid pairs among allelic contigs and separated the remaining valid pairs into 17 groups by their mapping relationship to contigs. For each group, the contigs and valid pairs were converted into PA5 format and used as input for YaHS v.1.2 (Zhou et al., 2023) to generate draft scaffolds and accompanying “.assembly” and “.hic” files. Fourth, the draft scaffolding files of each group were loaded into JuiceBox v.1.11.08 (Durand et al., 2016) and manually edited to correct scaffolding errors and generate the final scaffolds. For example, the absence of a Hi-C signal from the lower left to the upper right along the diagonal indicates a mis-join inside a contig (green border) or a scaffold (blue border), which can be corrected by a split at the mis-join position. The enrichment of the Hi-C signal like a bowtie, far away from the diagonal, indicates a mis-placement (similar to translocation) of the corresponding sequence, which can be corrected by moving the sequence to the correct place. The bowtie-like enrichment of the Hi-C signal near and parallel to the diagonal indicates a mis-orientation (similar to inversion) of the corresponding sequence, which can be corrected by reversing the sequence.

The first round of scaffolding work primarily produced nine groups of chromosome-level scaffolds and eight groups of chromosome-fragment-level scaffolds. These eight groups of scaffolds were then combined and loaded into JuiceBox for further manual curation to re-join the fragment-level scaffolds into chromosome-level scaffolds on the basis of reliable signals. Although we achieved near-chromosome-level scaffold assembly for all 17 groups, some chromosomes were much shorter than others in the same homologous group. In the second round of scaffolding, the largest pseudochromosome of each of the 17 homologous groups produced in the first round was selected to constitute a pseudo-monoploid genome of Jerusalem artichoke. These 17 selected chromosomes of Jerusalem artichoke were then used to replace the chromosomes of common sunflower in step 2 to avoid the mis-grouping of contigs caused by large-scale chromosome translocations between common sunflower and Jerusalem artichoke. In addition, the grouping of contigs that did not have translocations was also improved owing to the higher similarity of intra-species alignment. Through the second round of scaffolding work, the assembly quality of most chromosomes was improved, especially chromosomes from homologous groups with large-scale translocations. The assembly completeness and quality were assessed by mapping Illumina short reads with BWA v.0.7.17 (Li and Durbin, 2009) and HiFi long reads with Minimap2 v.2.20 (Li, 2018) and by BUSCO v.5.0 (Manni et al., 2021), K-mer based QV, Merqury v.1.3 (Rhie et al., 2020), and LAI from LTR_retriever v.2.9.0 (Ou and Jiang, 2018).

### Genome annotation

TRs and TEs were comprehensively annotated in the hexaploid genome of Jerusalem artichoke. TRs were identified using TRF v.4.07 (Benson, 1999). TEs were identified in three steps. First, structurally intact TEs of LTR retrotransposons, DNA transposons, and Helitron transposons were predicted in the whole genome using EDTA v.2.0.0 (Ou et al., 2019). Second, more homologous TEs with sequence similarity to the intact TEs, known TEs in the Repbase database v.26.05, and TE proteins were identified using RepeatMasker v.4.1.2 (http://repeatmasker.org/RepeatMasker/). Third, a *de novo* TE library was constructed using RepeatModeler v.2.0.2 (Flynn et al., 2020) and classified using TERL v.1.0 (Da Cruz et al., 2021); species-specific TEs were then identified in the genome using RepeatMasker v.4.1.2. According to our previous experience, TEs longer than 80 bp were soft masked in the hexaploid genome before gene prediction.

Protein-coding gene models were annotated in the TE soft-masked hexaploid genome of Jerusalem artichoke. The *ab initio* gene prediction parameters of Augustus v.3.4.0 were obtained from the intermediate results of the BUSCO evaluation of the genome assembly. The supporting hints of full-length transcripts were generated by aligning the pooled transcripts from rhizome, tuber, stem, leaf, and flower tissues to the hexaploid genome using GMAP v.2020-10-27 (Wu and Watanabe, 2005) with parameters “-n 6 --min-trimmed-coverage=95 --min-identity=95” and converting the results to a hints file using an Augustus script. Twenty-eight datasets of RNA-seq reads from various tissues, cultivars, and treatments were also mapped to the hexaploid genome using HISAT v.2.2.1 (Kim et al., 2019), assembled into gene structures using StringTie v.2.0 (Pertea et al., 2015), and converted into a hints file. The supporting hints of homologous proteins were generated by aligning the proteomes of 10 representative Asteroideae species (*H. annuus*, *Artemisiaannua*, *Erigeron canadensis*, *Arctium lappa*, *Artemisia argyi*, *Glebionis coronaria*, *Scalesia atractyloides*, *Smallanthus sonchifolius*, *Cichorium endivia*, and *Cichorium intybus*) to the hexaploid genome of Jerusalem artichoke using Miniprot v.0.7 (Li, 2023) with parameters “-u --gff” and converting the results to a hints file. The completeness of the predicted gene set for hexaploid Jerusalem artichoke was assessed using BUSCO v.5.0 with the eudicots_odb10 database (Manni et al., 2021). Augustus v.3.4.0 (Stanke et al., 2006) was then used to predict better gene models using the obtained gene prediction parameters and the input of supporting hints from transcripts and homologous proteins. Functional annotations of the protein-coding genes were obtained by searching against the NCBI-NR and KEGG databases using Diamond v.0.9.24 (Buchfink et al., 2015) and the InterPro database using InterProScan v.5.52-86 (Jones et al., 2014). Non-coding rRNA and tRNA genes were predicted using tRNAScan-SE v.2.0 (Lowe and Chan, 2016) and RNAmmer v.1.2 (Lagesen et al., 2007), respectively.

### Analysis of species phylogeny and genome polyploidization

The phylogenetic position and history of Jerusalem artichoke in the Asteraceae were reconstructed based on a concatenated sequence alignment of conserved OGs. The OGs among six Asteroideae species (hexaploid *H. tuberosus*, *H. annuus* [NCBI HanXRQr2.0-SUNRISE], *M. micrantha* [NCBI ASM936387v1], *Stevia rebaudiana* [FigShare 15169491.v1], *A. annua* [NCBI ASM311234v1], and *E. canadensis* [NCBI C_canadensis_v1]), two Cichorioideae species (*Lactuca sativa* [NCBI Lsat_ Salinax_v7] and *C. intybus* [NCBI ASM2352571v1]), two Carduoideae species (*A. lappa* [NCBI ASM2352574v1] and *Cynara cardunculus* [NCBI CcrdV1]), and one outgroup (*C. canephora* [NCBI AUK_PRJEB4211_v1]) were obtained using OrthoFinder v.2.5.2 (Emms and Kelly, 2019) with parameters “-M msa -A mafft -T fasttree -1 -y.” From all OGs, we selected 1136 conserved OGs, each of which had 6 gene copies in *H. tuberosus* but only one gene in the other species. Then, one representative gene from *H. tuberosus* and all single-copy genes from other species were used to independently build the MSAs for these 1136 conserved OGs with Muscle v.3.8.31 (Edgar, 2004). All the MSAs were joined together to form a concatenated MSA (CMSA), which was used for species tree construction in RAxML-NG v.1.0.3 with GTR mode (Kozlov et al., 2019) and for divergence time tree construction using the RelTime-ML method in MEGA v.11 (Tamura et al., 2012) with one calibration point, the divergence of *C. cardunculus* and *L. sativa* 37–45 mya obtained from TimeTree5 (Kumar et al., 2022).

Whole-genome polyploidization events for Jerusalem artichoke were investigated by analyzing chromosome-level macro-synteny and the Ks distribution of syntenic gene pairs. All-vs-all alignment was performed for the proteome sequences of hexaploid Jerusalem artichoke using Diamond v.0.9.24 (Buchfink et al., 2015), and the results were used as input for MCScanX (Wang et al., 2012) to identify syntenic genomic blocks with more than 10 genes. We used the R package ggplot2 to draw the intra-species syntenic gene dot plot for hexaploid Jerusalem artichoke. The Ks values of syntenic gene pairs were calculated using KaKs_Calculator v.2.0 (Wang et al., 2010) with the GMYN model, and the clear Ks peaks indicating WGDs or WGTs were verified by chromosome-level macro-synteny and previously reported WGD or WGT events in Asteraceae.

### Analysis of chromosome rearrangements

To discover the chromosome break and fusion events, we performed a chromosome-level synteny analysis between Jerusalem artichoke and common sunflower using MCScanX (Wang et al., 2012). Inter-species syntenic genomic blocks with more than 10 genes were visualized using the R package RIdeogram (Hao et al., 2020). Because the 6 homologous chromosomes in each Jerusalem artichoke group showed nearly identical syntenic relationships with common sunflower chromosomes, we selected 17 chromosomes, each representing one homologous group, to make comparisons with 17 common sunflower chromosomes and infer the chromosome breaks and fusions that happened in the *Helianthus* lineage.

### Divergence analysis of homologous chromosomes

We analyzed the sequence divergence among homologous chromosomes to elucidate the hexaploid genome structure of Jerusalem artichoke and determine whether this crop is an autoallopolyploid with a hybridization origin. First, the genomic sequences of each group of six homologous chromosomes were aligned to each other using Nucmer in MUMmer v.4 (Marcais et al., 2018), and the identities of alignment blocks between any two homologous chromosomes were used to infer whether they came from the same subgenome or two different subgenomes, assuming that the former would have a higher identity than the latter. Second, we identified the conserved single-copy genes among the six homologous chromosomes of each group using OrthoFinder v.2.5.2 (Emms and Kelly, 2019) and calculated the alignment identities between any two homologous chromosomes using their single-copy genes. Conserved single-copy genes were shown to better discriminate homologous chromosomes from the same subgenome or two different subgenomes.

### Inferring the history of hexaploid origin and evolution

Treating each chromosome in one homologous group of Jerusalem artichoke as a virtual species, we constructed an unrooted phylogenetic tree of the six homologous chromosomes using RaxML-NG v.1.0.3 with LG+G8+F mode (Kozlov et al., 2019) based on the CMSA of conserved single-copy genes. To minimize interference from alignment gaps, we used trimAl v.1.2 (Capella-Gutiérrez et al., 2009) to trim the MSA of single-copy genes before phylogenetic tree construction. For most homologous chromosome groups, the CMSA of all single-copy genes was used for phylogenetic tree construction; for chr01, chr05, and chr15, the CMSA of single-copy genes with alignment identity of 92%–100% was used for phylogenetic tree construction. Overall, the unrooted trees of homologous chromosomes were shown to be effective in clarifying the genome structure and hybridization origin of hexaploid Jerusalem artichoke.

To further determine the timeline of the hybridization origin and subsequent chromosome doubling during the evolution of hexaploid Jerusalem artichoke, we also treated each chromosome in each homologous group as a virtual species and identified more conserved single-copy genes among Jerusalem artichoke, common sunflower, and *M. micrantha* using OrthoFinder v.2.5.2 (Emms and Kelly, 2019). Then, for each homologous group, the divergence time tree was inferred using the RelTime-ML method in MEGA11 (Tamura et al., 2021) with the phylogenetic tree constructed from the trimmed CMSA of single-copy genes and one calibration time point obtained from TimeTree5, the divergence of common sunflower and *M. micrantha* 16–27 mya (Kumar et al., 2022). The estimated divergence times of three pairs of homologous chromosomes from the same subgenome indicate when inter-species hybridization and chromosome doubling occurred for the ancestors of Jerusalem artichoke.

### Identification and phylogeny of inulin metabolism genes

To find all inulin metabolism genes in the hexaploid genome of Jerusalem artichoke, we integrated multiple methods to identify both true genes and pseudogenes derived from multiple rounds of genome polyploidization. First, the previously cloned and functionally verified inulin metabolism genes of Jerusalem artichoke, *1-SST* (NCBI protein CAA08812.1), *1-FFT* (NCBI protein CAA08811.1), *1-FEHI* (NCBI protein AJW31155.1), and *1-FEHII* (NCBI protein AJW31156.1), were downloaded and searched against the proteome of the hexaploid genome using Diamond v.0.9.24 (Buchfink et al., 2015) with the parameter “--more-sensitive.” Protein hits to the protein sequences of the above four genes with both identity and coverage higher than 90% were retained as candidate inulin metabolism genes. Second, the candidate genes were checked for the presence of N-terminal (Pfam PF00251) and C-terminal (Pfam PF08244) domains of glycosyl hydrolase family 32 using HMMER v.3.1b2 (Potter et al., 2018). We also checked whether the candidate genes were grouped into the OGs that contained inulin metabolism genes of common sunflower and other Asteraceae species using the results of OrthoFinder v.2.5.2 (Emms and Kelly, 2019). Through the above approaches, the structurally complete, true inulin metabolism genes of hexaploid Jerusalem artichoke were identified.

We also identified inulin metabolism pseudogenes by aligning the known protein sequences of *1-SST*, *1-FFT*, *1-FEHI*, and *1-FEHII* to the genomic sequences using Exonerate v.2.2.0 (Slater and Birney, 2005). Newly predicted genes with internal stop codons, frameshifts, or truncated exons relative to the true inulin metabolism genes were identified as pseudogenes. We then used Muscle v.3.8.31 (Edgar, 2004) to perform MSA and FastTree v.2.0 with LG mode (Price et al., 2010) to construct phylogenetic trees for all inulin metabolism genes in hexaploid Jerusalem artichoke and their orthologs in common sunflower and chicory. Combined with the chromosome locations of inulin metabolism genes, these trees enabled us to clarify the origins and evolutionary fates of inulin metabolism genes during WGD2, WGT3, and complex mutation processes.

### Expression of inulin metabolism genes and potential TFs

To determine the expression patterns of inulin metabolism genes and find genes encoding their potential regulatory TFs, we collected 92 datasets of Jerusalem artichoke RNA-seq reads from tuber and leaf tissues, various growth stages, various cultivars, and various periods of water and drought stress and mapped them to the hexaploid genome using HISAT v.2.2.1 (Kim et al., 2019). To exclude the interference of multiple mappings on the expression profiling of homologous genes with high similarity, the mapped BAM files were filtered using SAMtools v.1.3 with parameters “-f 2 -F 256 -q 30” to retain only high-quality and properly mapped read pairs. We then used StringTie v.2.0 (Pertea et al., 2015) with the gene annotation GFF file of Jerusalem artichoke and the filtered RNA-seq mapped BAM files as input to calculate gene expression levels as fragments per kilobase per million fragments. The expression matrix of all genes in different samples was used for WGCNA to identify network modules containing inulin metabolism genes and TFs using the R package WGCNA (Langfelder and Horvath, 2008). TF genes were annotated using the online TF prediction tool of PlantRegMap v.5.0 (Tian et al., 2020), and the promoter regions (upstream 2 kb) of inulin metabolism genes were scanned for TF binding motifs using the FunTFBS tool in PlantRegMap v.5.0 (Tian et al., 2020). Afterward, TFs that potentially regulated the expression of inulin metabolism genes were visualized as networks using Cytoscape v.3.8.0 (Shannon et al., 2003). Expression heatmaps of inulin metabolism genes (true genes) and potential TF genes in tuber and leaf tissues from different stages, conditions, or cultivars were drawn using TBtools v.1.113 (Chen et al., 2020b).

## Data and code availability

The genomic and transcriptomic sequencing reads generated in this study have been deposited at the NCBI Sequence Read Archive under accession PRJNA918503 and the Sequence Archive of the China National GeneBank (CNGB) under accession CNP0004182. The hexaploid genome assembly and annotation have been deposited at NCBI GenBank under accession JARYGA000000000 and the Genome Warehouse of the National Genomics Data Center (NGDC) under accession GWHEQHM00000000; they are also available at Figshare (https://doi.org/10.6084/m9.figshare.22491205.v1).

## Funding

This work was supported by the 10.13039/501100012166National Key R&D Program of China (2021YFC2600101), the Shenzhen Science and Technology Program (JCYJ20190814163805604 and KQTD20180411143628272), the Fund of the Key Laboratory of Shenzhen (ZDSYS20141118170111640), and the 10.13039/501100012421Agricultural Science and Technology Innovation Program.

## Author contributions

W.F. conceived the study, and S.W. designed the study. A.W. and R.C. prepared the genomic and transcriptomic sequencing samples. S.W., A.W., R.C., D.X., H.W., F.J., and H.L. completed the bioinformatic analyses. S.W., R.C., and A.W. prepared the tables, figures, and supplemental information. S.W. and A.W. wrote the manuscript. W.F. and W.Q. supervised the project and revised the manuscript, and all authors read and approved the final version of the manuscript.

## Supplemental information

Document S1. Supplemental Figures 1–17 and Supplemental Tables 1–16

Document S2. Article plus supplemental information

## References

[bib1] Atlagić J., Dozet B., ŠKorić D. (1993). Meiosis and Pollen Viability in Helianthus tuberosus L. and its Hybrids with Cultivated Sunflower. Plant Breed..

[bib2] Badouin H., Gouzy J., Grassa C.J., Murat F., Staton S.E., Cottret L., Lelandais-Brière C., Owens G.L., Carrère S., Mayjonade B. (2017). The sunflower genome provides insights into oil metabolism, flowering and Asterid evolution. Nature.

[bib3] Bao Z., Li C., Li G., Wang P., Peng Z., Cheng L., Li H., Zhang Z., Li Y., Huang W. (2022). Genome architecture and tetrasomic inheritance of autotetraploid potato. Mol. Plant.

[bib4] Barb J.G., Bowers J.E., Renaut S., Rey J.I., Knapp S.J., Rieseberg L.H., Burke J.M. (2014). Chromosomal Evolution and Patterns of Introgression in *Helianthus*. Genetics.

[bib5] Benson G. (1999). Tandem repeats finder: a program to analyze DNA sequences. Nucleic Acids Res..

[bib6] Binghang L., Shi Y., Yuan J., Galaxy Y., Zhang H., Li N., Li Z., Chen Y., Mu D., Fan W. (2013). Estimation of genomic characteristics by analyzing k-mer frequency in de novo genome projects. Advance Access.

[bib7] Bock D.G., Kane N.C., Ebert D.P., Rieseberg L.H. (2014). Genome skimming reveals the origin of the Jerusalem Artichoke tuber crop species: neither from Jerusalem nor an artichoke. New Phytol..

[bib8] Buchfink B., Xie C., Huson D.H. (2015). Fast and sensitive protein alignment using DIAMOND. Nat. Methods.

[bib9] Capella-Gutiérrez S., Silla-Martínez J.M., Gabaldón T. (2009). trimAl: a tool for automated alignment trimming in large-scale phylogenetic analyses. Bioinformatics.

[bib10] Chen H., Zeng Y., Yang Y., Huang L., Tang B., Zhang H., Hao F., Liu W., Li Y., Liu Y. (2020). Allele-aware chromosome-level genome assembly and efficient transgene-free genome editing for the autotetraploid cultivated alfalfa. Nat. Commun..

[bib11] Chen C., Chen H., Zhang Y., Thomas H.R., Frank M.H., He Y., Xia R. (2020). TBtools: An Integrative Toolkit Developed for Interactive Analyses of Big Biological Data. Mol. Plant.

[bib12] Cheng H., Concepcion G.T., Feng X., Zhang H., Li H. (2021). Haplotype-resolved de novo assembly using phased assembly graphs with hifiasm. Nat. Methods.

[bib13] Da Cruz M.H.P., Domingues D.S., Saito P.T.M., Paschoal A.R., Bugatti P.H. (2021). TERL: classification of transposable elements by convolutional neural networks. Briefings Bioinf..

[bib14] Denoeud F., Carretero-Paulet L., Dereeper A., Droc G., Guyot R., Pietrella M., Zheng C., Alberti A., Anthony F., Aprea G. (2014). The coffee genome provides insight into the convergent evolution of caffeine biosynthesis. Science.

[bib15] Durand N.C., Robinson J.T., Shamim M.S., Machol I., Mesirov J.P., Lander E.S., Aiden E.L. (2016). Juicebox Provides a Visualization System for Hi-C Contact Maps with Unlimited Zoom. Cell Syst..

[bib16] Edgar R.C. (2004). MUSCLE: multiple sequence alignment with high accuracy and high throughput. Nucleic Acids Res..

[bib17] Emms D.M., Kelly S. (2019). OrthoFinder: phylogenetic orthology inference for comparative genomics. Genome Biol..

[bib18] Ende W.V.D. (2013). Multifunctional fructans and raffinose family oligosaccharides. Front. Plant Sci..

[bib19] Fan W., Wang S., Wang H., Wang A., Jiang F., Liu H., Zhao H., Xu D., Zhang Y. (2022). The genomes of chicory, endive, great burdock and yacon provide insights into Asteraceae palaeo-polyploidization history and plant inulin production. Mol. Ecol. Resour..

[bib20] Flynn J.M., Hubley R., Goubert C., Rosen J., Clark A.G., Feschotte C., Smit A.F. (2020). RepeatModeler2 for automated genomic discovery of transposable element families. Proc. Natl. Acad. Sci. USA.

[bib21] Hao Z., Lv D., Ge Y., Shi J., Weijers D., Yu G., Chen J. (2020). RIdeogram: drawing SVG graphics to visualize and map genome-wide data on the idiograms. PeerJ. Comput. Sci..

[bib22] Jones P., Binns D., Chang H.-Y., Fraser M., Li W., McAnulla C., McWilliam H., Maslen J., Mitchell A., Nuka G. (2014). InterProScan 5: genome-scale protein function classification. Bioinformatics.

[bib23] Kantar M.B., Hüber S., Herman A., Bock D.G., Baute G., Betts K., Ott M., Brandvain Y., Wyse D., Stupar R.M., Rieseberg L.H. (2018). Neo-Domestication of an Interspecific Tetraploid Helianthus annuus × Helianthus tuberous Population That Segregates for Perennial Habit. Genes.

[bib24] Khuenpet K., Jittanit W., Sirisansaneeyakul S., Srichamnong W. (2017). Inulin Powder Production from Jerusalem Artichoke (*Helianthus tuberosus* L.) Tuber Powder and Its Application to Commercial Food Products: INULIN POWDER PRODUCTION AND ITS APPLICATION. J. Food Process. Preserv..

[bib25] Kim D., Paggi J.M., Park C., Bennett C., Salzberg S.L. (2019). Graph-based genome alignment and genotyping with HISAT2 and HISAT-genotype. Nat. Biotechnol..

[bib26] Kong W., Wang Y., Zhang S., Yu J., Zhang X. (2023). Recent Advances in Assembly of Plant Complex Genomes. Dev. Reprod. Biol..

[bib27] Koops A.J., Jonker H.H. (1994). Purification and characterization of the enzymes of fructan biosynthesis in tubers of Helianthus tuberosus ‘Colombia’: I. Fructan: fructan fructosyl transferase. J. Exp. Bot..

[bib28] Kostoff D. (1939). Autosyndesis and structural hybridity in F1-hybrid Helianthus tuberosus L. x Helianthus annuus L. and their sequences. Genetica.

[bib29] Kozlov A.M., Darriba D., Flouri T., Morel B., Stamatakis A. (2019). RAxML-NG: a fast, scalable and user-friendly tool for maximum likelihood phylogenetic inference. Bioinformatics.

[bib30] Kumar S., Suleski M., Craig J.M., Kasprowicz A.E., Sanderford M., Li M., Stecher G., Hedges S.B. (2022). TimeTree 5: An Expanded Resource for Species Divergence Times. Mol. Biol. Evol..

[bib31] Lagesen K., Hallin P., Rødland E.A., Stærfeldt H.-H., Rognes T., Ussery D.W. (2007). RNAmmer: consistent and rapid annotation of ribosomal RNA genes. Nucleic Acids Res..

[bib32] Langfelder P., Horvath S. (2008). WGCNA: an R package for weighted correlation network analysis. BMC Bioinf..

[bib33] Li H. (2018). Minimap2: pairwise alignment for nucleotide sequences. Bioinformatics.

[bib34] Li H. (2023). Protein-to-genome alignment with miniprot. Bioinformatics.

[bib35] Li H., Durbin R. (2009). Fast and accurate short read alignment with Burrows–Wheeler transform. Bioinformatics.

[bib36] Lowe T.M., Chan P.P. (2016). tRNAscan-SE On-line: integrating search and context for analysis of transfer RNA genes. Nucleic Acids Res..

[bib37] Lv S., Wang R., Xiao Y., Li F., Mu Y., Lu Y., Gao W., Yang B., Kou Y., Zeng J., Zhao C. (2019). Growth, yield formation, and inulin performance of a non-food energy crop, Jerusalem artichoke (Helianthus tuberosus L.), in a semi-arid area of China. Ind. Crop. Prod..

[bib38] Mandel J.R., Dikow R.B., Siniscalchi C.M., Thapa R., Watson L.E., Funk V.A. (2019). A fully resolved backbone phylogeny reveals numerous dispersals and explosive diversifications throughout the history of Asteraceae. Proc. Natl. Acad. Sci. USA.

[bib39] Manni M., Berkeley M.R., Seppey M., Simão F.A., Zdobnov E.M. (2021). BUSCO Update: Novel and Streamlined Workflows along with Broader and Deeper Phylogenetic Coverage for Scoring of Eukaryotic, Prokaryotic, and Viral Genomes. Mol. Biol. Evol..

[bib40] Marçais G., Delcher A.L., Phillippy A.M., Coston R., Salzberg S.L., Zimin A. (2018). MUMmer4: A fast and versatile genome alignment system. PLoS Comput. Biol..

[bib41] Ostevik K.L., Samuk K., Rieseberg L.H. (2020). Ancestral Reconstruction of Karyotypes Reveals an Exceptional Rate of Nonrandom Chromosomal Evolution in Sunflower. Genetics.

[bib42] Ou S., Jiang N. (2018). LTR_retriever: A Highly Accurate and Sensitive Program for Identification of Long Terminal Repeat Retrotransposons. Plant Physiol..

[bib43] Ou S., Su W., Liao Y., Chougule K., Agda J.R.A., Hellinga A.J., Lugo C.S.B., Elliott T.A., Ware D., Peterson T. (2019). Benchmarking transposable element annotation methods for creation of a streamlined, comprehensive pipeline. Genome Biol..

[bib44] Owens G.L., Huang K., Todesco M., Rieseberg L.H. (2023). Re-evaluating Homoploid Reticulate Evolution in *Helianthus* Sunflowers. Mol. Biol. Evol..

[bib45] Pertea M., Pertea G.M., Antonescu C.M., Chang T.-C., Mendell J.T., Salzberg S.L. (2015). StringTie enables improved reconstruction of a transcriptome from RNA-seq reads. Nat. Biotechnol..

[bib46] Potter S.C., Luciani A., Eddy S.R., Park Y., Lopez R., Finn R.D. (2018). HMMER web server: 2018 update. Nucleic Acids Res..

[bib47] Price M.N., Dehal P.S., Arkin A.P. (2010). FastTree 2 – Approximately Maximum-Likelihood Trees for Large Alignments. PLoS One.

[bib48] Qiu F., Baack E.J., Whitney K.D., Bock D.G., Tetreault H.M., Rieseberg L.H., Ungerer M.C. (2019). Phylogenetic trends and environmental correlates of nuclear genome size variation in *Helianthus* sunflowers. New Phytol..

[bib49] Rhie A., Walenz B.P., Koren S., Phillippy A.M. (2020). Merqury: reference-free quality, completeness, and phasing assessment for genome assemblies. Genome Biol..

[bib50] Rieseberg L., Burke J.M. (2008). Molecular evidence and the origin of the domesticated sunflower. Proc. Natl. Acad. Sci. USA.

[bib51] Servant N., Varoquaux N., Lajoie B.R., Viara E., Chen C.-J., Vert J.-P., Heard E., Dekker J., Barillot E. (2015). HiC-Pro: an optimized and flexible pipeline for Hi-C data processing. Advance Access.

[bib52] Shannon P., Markiel A., Ozier O., Baliga N.S., Wang J.T., Ramage D., Amin N., Schwikowski B., Ideker T. (2003). Cytoscape: A Software Environment for Integrated Models of Biomolecular Interaction Networks. Genome Res..

[bib53] Slater G.S.C., Birney E. (2005). Automated generation of heuristics for biological sequence comparison. BMC Bioinf..

[bib54] Stanke M., Schöffmann O., Morgenstern B., Waack S. (2006). Gene prediction in eukaryotes with a generalized hidden Markov model that uses hints from external sources. BMC Bioinf..

[bib55] Tamura K., Battistuzzi F.U., Billing-Ross P., Murillo O., Filipski A., Kumar S. (2012). Estimating divergence times in large molecular phylogenies. Proc. Natl. Acad. Sci. USA.

[bib56] Tamura K., Stecher G., Kumar S. (2021). MEGA11: Molecular Evolutionary Genetics Analysis Version 11. Mol. Biol. Evol..

[bib57] Tanjor S., Judprasong K., Suagpuag P., Puwastien P., Jogloy S. (2009). Annals of Nutrition and Metabolism.

[bib58] Tian F., Yang D.-C., Meng Y.-Q., Jin J., Gao G. (2020). PlantRegMap: charting functional regulatory maps in plants. Nucleic Acids Res..

[bib59] Valluru R. (2015). Fructan and hormone connections. Front. Plant Sci..

[bib60] Valluru R., Van Den Ende W. (2008). Plant fructans in stress environments: emerging concepts and future prospects. J. Exp. Bot..

[bib61] Van De Peer Y., Ashman T.-L., Soltis P.S., Soltis D.E. (2021). Polyploidy: an evolutionary and ecological force in stressful times. Plant Cell.

[bib62] Van Der Meer I.M., Koops A.J., Hakkert J.C., Van Tunen A.J. (1998). Cloning of the fructan biosynthesis pathway of Jerusalem artichoke. Plant J..

[bib63] Vijn I., Smeekens S. (1999). Fructan: More Than a Reserve Carbohydrate?1. Plant Physiol..

[bib64] Wang D., Zhang Y., Zhang Z., Zhu J., Yu J. (2010). KaKs_Calculator 2.0: A Toolkit Incorporating Gamma-Series Methods and Sliding Window Strategies. Dev. Reprod. Biol..

[bib65] Wang Y., Tang H., DeBarry J.D., Tan X., Li J., Wang X., Lee T. -h., Jin H., Marler B., Guo H. (2012). MCScanX: a toolkit for detection and evolutionary analysis of gene synteny and collinearity. Nucleic Acids Res..

[bib66] Wei H., Bausewein A., Greiner S., Dauchot N., Harms K., Rausch T. (2017). Ci MYB 17, a stress-induced chicory R2R3- MYB transcription factor, activates promoters of genes involved in fructan synthesis and degradation. New Phytol..

[bib67] Wei H., Zhao H., Su T., Bausewein A., Greiner S., Harms K., Rausch T. (2017). Chicory R2R3-MYB transcription factors CiMYB5 and CiMYB3 regulate fructan 1-exohydrolase expression in response to abiotic stress and hormonal cues. J. Exp. Bot..

[bib68] Wu T.D., Watanabe C.K. (2005). GMAP: a genomic mapping and alignment program for mRNA and EST sequences. Bioinformatics.

[bib69] Wu S., Han B., Jiao Y. (2020). Genetic Contribution of Paleopolyploidy to Adaptive Evolution in Angiosperms. Mol. Plant.

[bib70] Xu H., Liang M., Xu L., Li H., Zhang X., Kang J., Zhao Q., Zhao H. (2015). Cloning and functional characterization of two abiotic stress-responsive Jerusalem artichoke (Helianthus tuberosus) fructan 1-exohydrolases (1-FEHs). Plant Mol. Biol..

[bib71] Yang S., Zhong Q., Tian J., Wang L., Zhao M., Li L., Sun X. (2018). Characterization and development of EST-SSR markers to study the genetic diversity and populations analysis of Jerusalem artichoke (Helianthus tuberosus L.). Genes Genom.

[bib72] Zhang X., Zhang S., Zhao Q., Ming R., Tang H. (2019). Assembly of allele-aware, chromosomal-scale autopolyploid genomes based on Hi-C data. Nat. Plants.

[bib73] Zhang C., Huang C.-H., Liu M., Hu Y., Panero J.L., Luebert F., Gao T., Ma H. (2021). Phylotranscriptomic insights into Asteraceae diversity, polyploidy, and morphological innovation. J. Integr. Plant Biol..

[bib74] Zhang Q., Qi Y., Pan H., Tang H., Wang G., Hua X., Wang Y., Lin L., Li Z., Li Y. (2022). Genomic insights into the recent chromosome reduction of autopolyploid sugarcane Saccharum spontaneum. Nat. Genet..

[bib75] Zhao M., Zhong Q., Tian M., Han R., Ren Y. (2020). Comparative transcriptome analysis reveals differentially expressed genes associated with the development of Jerusalem artichoke tuber (Helianthus tuberosus L.). Ind. Crop. Prod..

[bib76] Zhou C., McCarthy S.A., Durbin R. (2023). YaHS: yet another Hi-C scaffolding tool. Bioinformatics.

