# Supplementary material for: Haplotype-resolved chromosome-level genome of hexaploid Jerusalem artichoke provides insights into its origin, evolution, and inulin metabolism
Source: Plant Commun. 2023 Nov 17;5(3):100767. doi: 10.1016/j.xplc.2023.100767 (PMC10943552; doi:10.1016/j.xplc.2023.100767)
Supplement: Document S1. Supplemental Figures 1–17 and Supplemental Tables 1–16 [file mmc1.pdf]

Plant Communications, Volume *5*

Supplemental information

Haplotype-resolved chromosome-level genome of hexaploid Jerusalem artichoke provides insights into its origin, evolution, and inulin metabolism

Sen Wang, Anqi Wang, Rong Chen, Dong Xu, Hengchao Wang, Fan Jiang, Hangwei Liu, Wanqiang Qian, and Wei Fan

Supplementary tables and figures for:

A haplotype-resolved chromosome-level genome assembly for the hexaploid Jerusalem artichoke provides insights into its origin, evolution and inulin metabolism

Sen Wang[1,2,4], Anqi Wang[1,4], Rong Chen[1,3,4], Dong Xu[1], Hengchao Wang[1], Fan Jiang[1], Hangwei Liu[1], Wanqiang Qian[1], Wei Fan[1,*]

[1]Guangdong Laboratory for Lingnan Modern Agriculture (Shenzhen Branch), Genome Analysis Laboratory of the Ministry of Agriculture and Rural Affairs, Agricultural Genomics Institute at Shenzhen, Chinese Academy of Agricultural Sciences, Shenzhen, Guangdong, 518120, China.

[2]Guangdong Provincial Key Laboratory for Crop Germplasm Resources Preservation and Utilization, Agro-Biological Gene Research Center, Guangdong Academy of Agricultural Sciences, Guangzhou 510640, China.

[3]College of Agronomy, Qingdao Agricultural University, Qingdao, 266109, China

[4]These authors contributed equally: Sen Wang, Anqi Wang, Rong Chen.

[*]Correspondence author: Wei Fan, fanwei@caas.cn.

This file includes:

Supplemental Figure 1-17

Supplemental Table 1-16

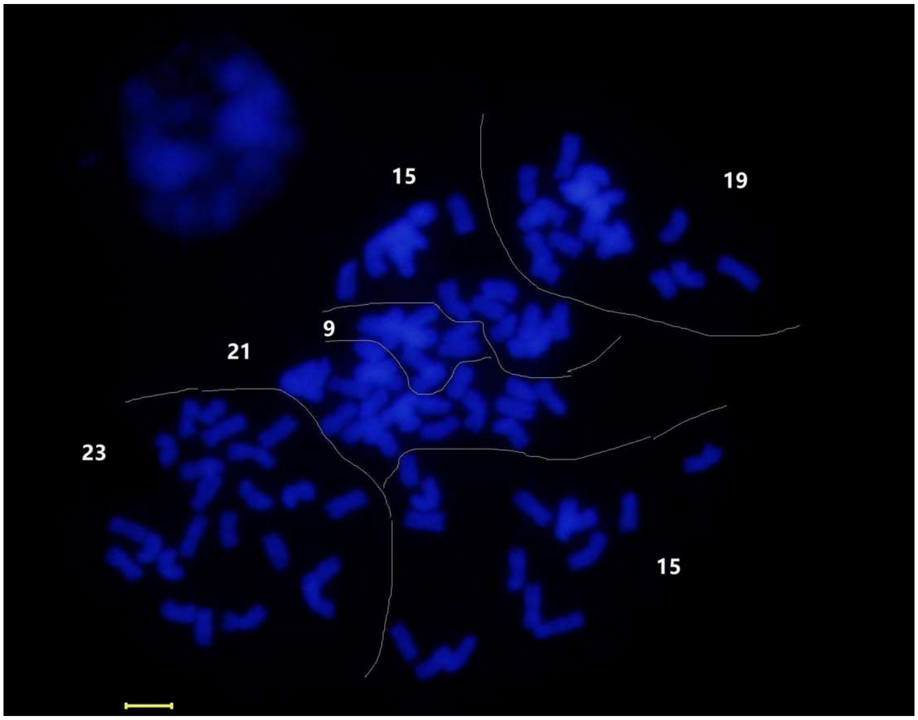

Supplemental Figure 1 Karyotype of the sequenced cultivar of *Helianthus tuberosus* in this study. Chromosomes are stained to blue by the fluorescence dye DAPI. Numbers in white indicate the number of chromosomes in the corresponding outlined region and the bar on the bottom left indicates 5 μm.

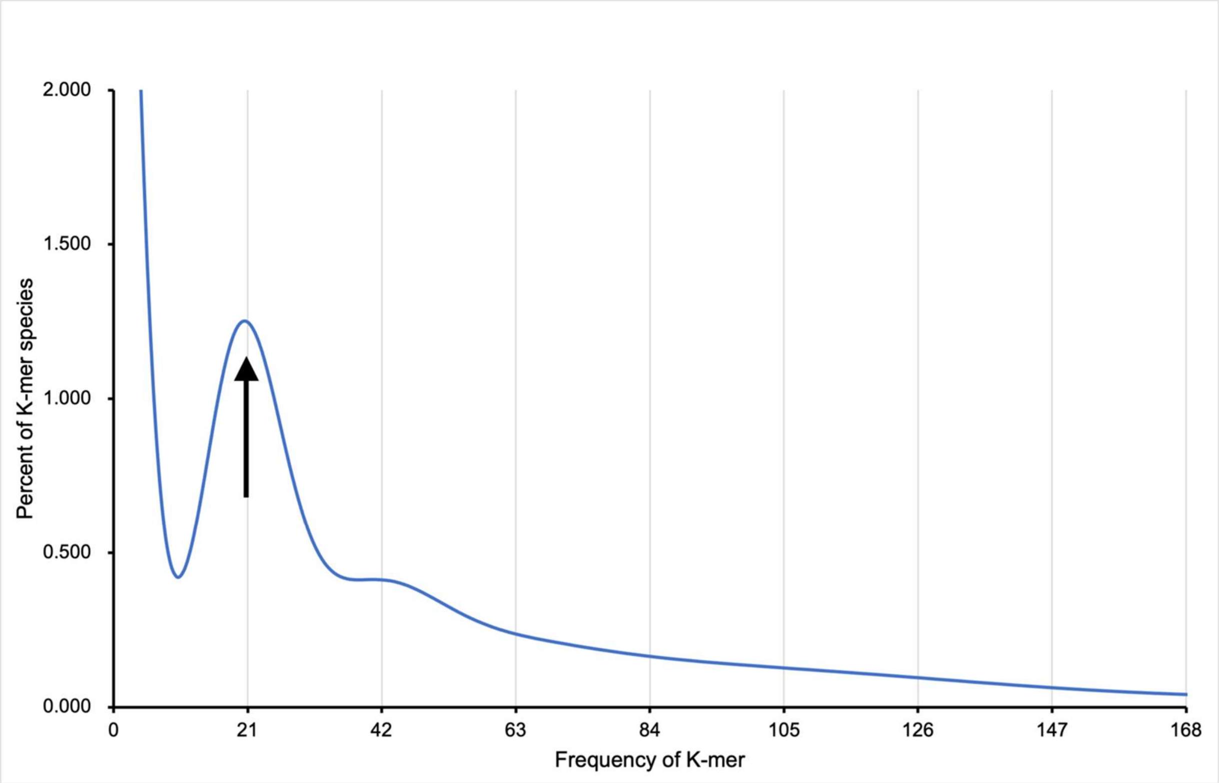

Supplemental Figure 2 Estimation of genomic characteristics of hexaploid *H. tuberosus* by K-mer (K = 19) analysis. The Illumina short reads of genome sequencing were used for 19-mer counting and frequency distribution analysis. X-axis: frequency of K-mer; Y-axis: percent of K-mer species. The black arrow indicates the major peak generated by K-mers from the unique genomic regions, the long tail (too high to be shown) on the left of the major peak indicates K-mers from sequencing errors, and the peaks on the right of the major peak indicate K-mers from the similar homologous regions and repetitive regions. Use the formula (total K-mer number / Major peak value), the hexaploid genome size was estimated to be about 20.8 Gb.

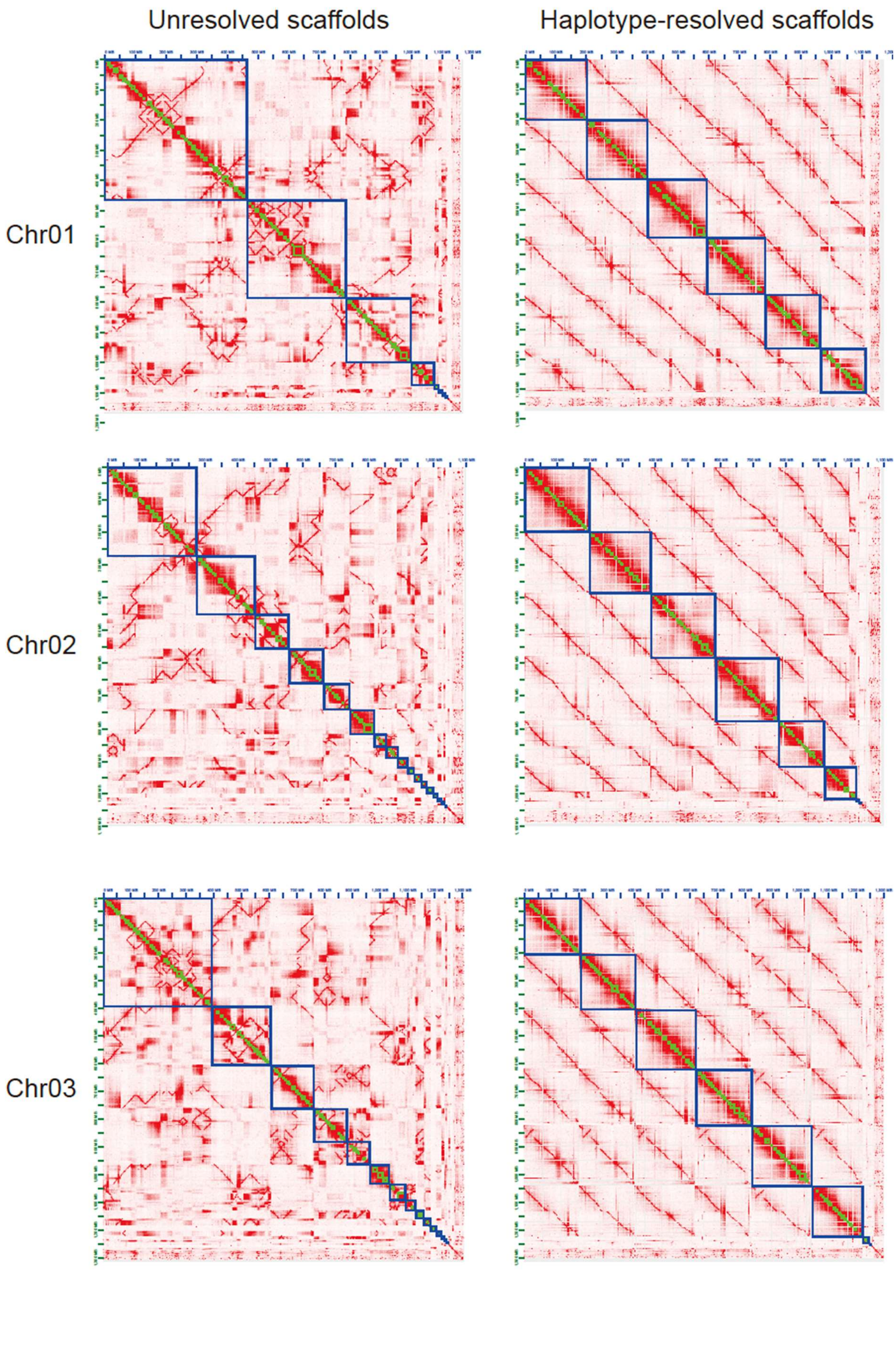
Unresolved scaffolds
Haplotype-resolved scaffolds
Chr01
Chr02
Chr03

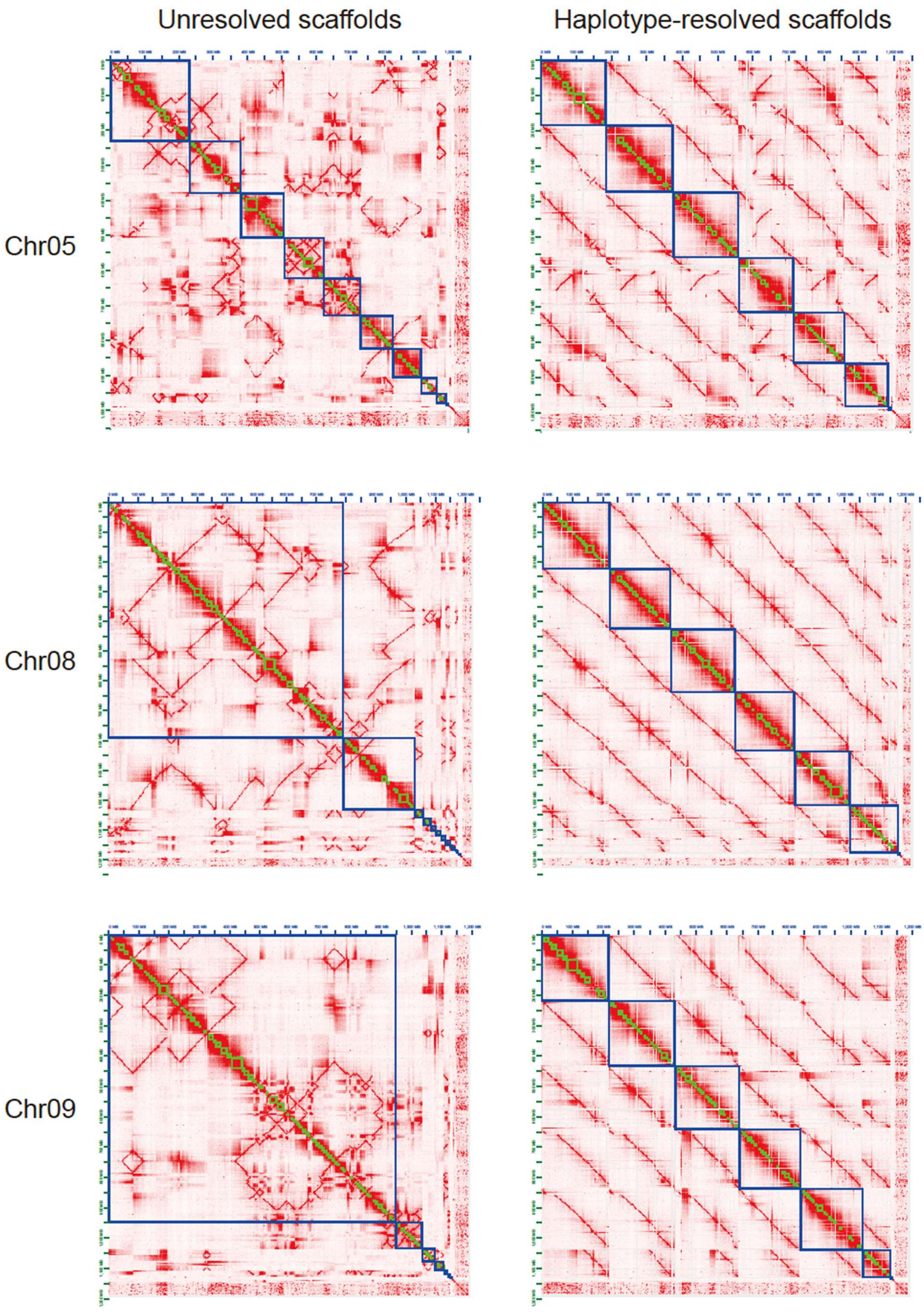
Unresolved scaffolds
Haplotype-resolved scaffolds
Chr05
Chr08
Chr09

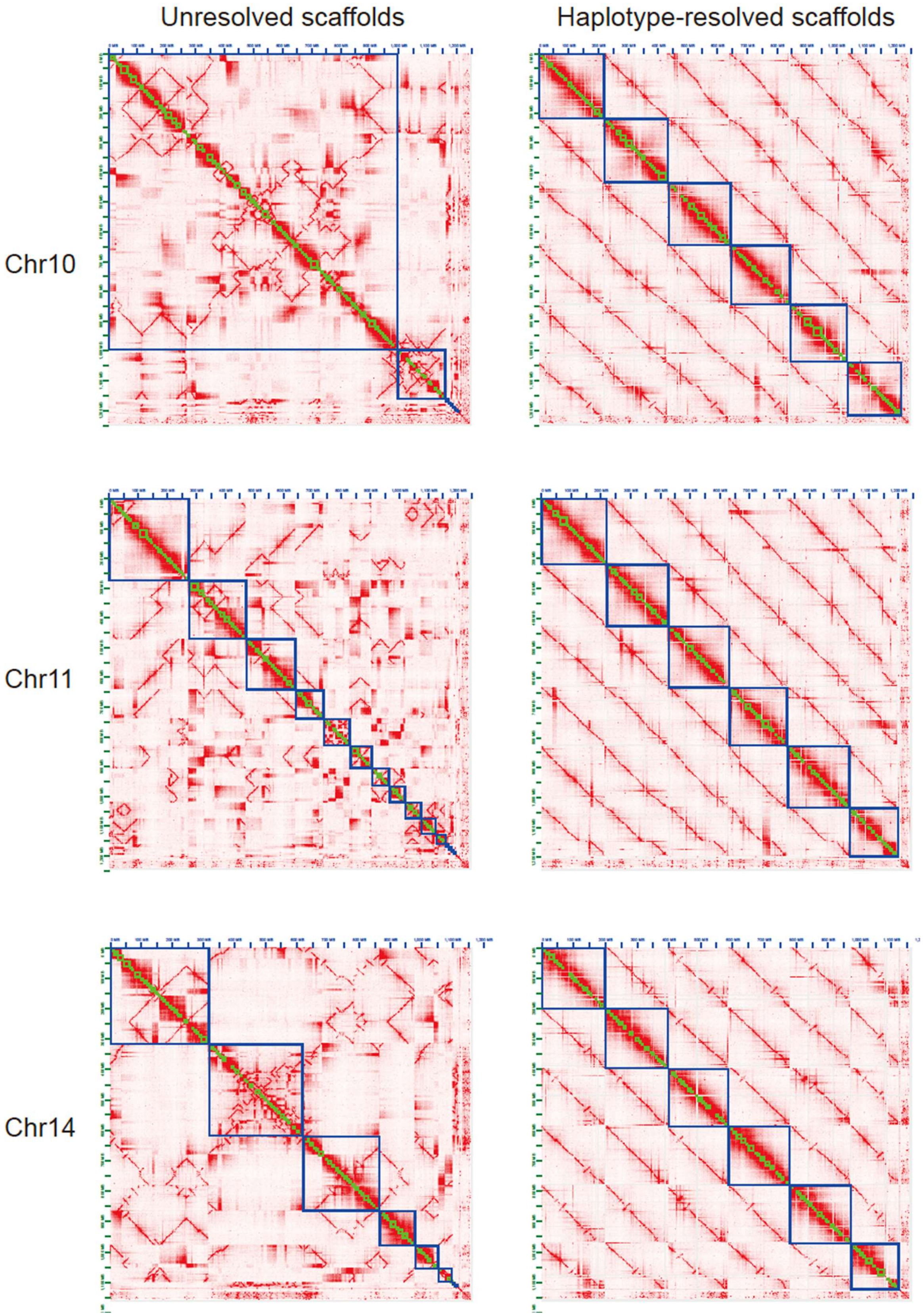
Unresolved scaffolds
Haplotype-resolved scaffolds
Chr10
Chr11
Chr14

Supplemental Figure 3 First-round Hi-C scaffolding of 9 groups of homologous pseudochromosomes for hexaploid *H. tuberosus*. The contigs and mapped Hi-C reads of each homologous group were generated according to the contig alignments to the chromosome-level reference genome of *H. annuus*, and for each group, the unresolved scaffolding results (on the left) were produced using YaHS and the haplotype-resolved scaffolding results (on the right) were generated by manual curation in JuiceBox. In the Hi-C contact heatmap of unresolved and haplotype-resolved scaffolds, green and blue squares indicate contigs and scaffolds, respectively, and the color darkness of each pixel is proportional to the Hi-C contact density between the two corresponding genomic regions.

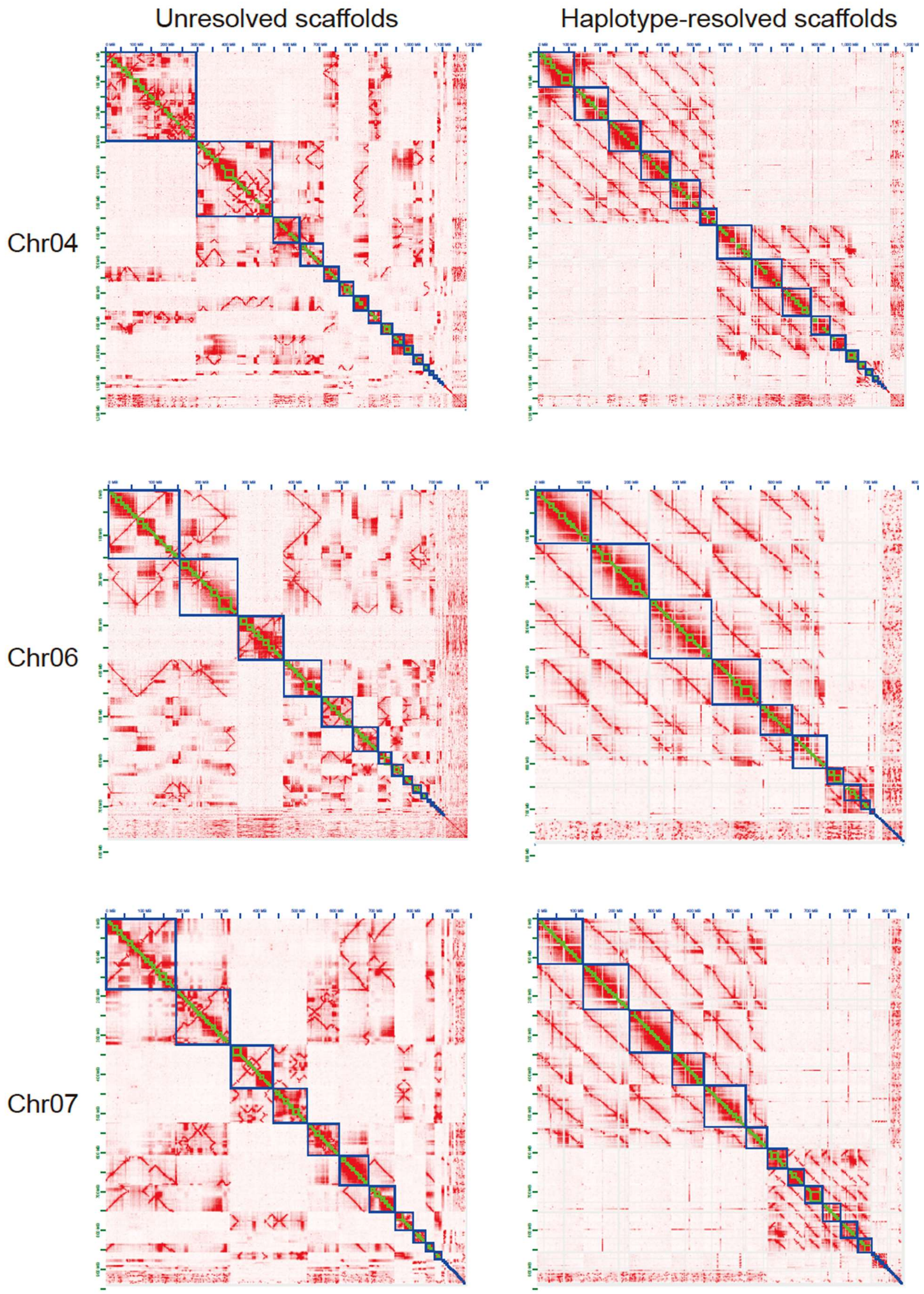
Unresolved scaffolds
Haplotype-resolved scaffolds
Chr04
Chr06
Chr07

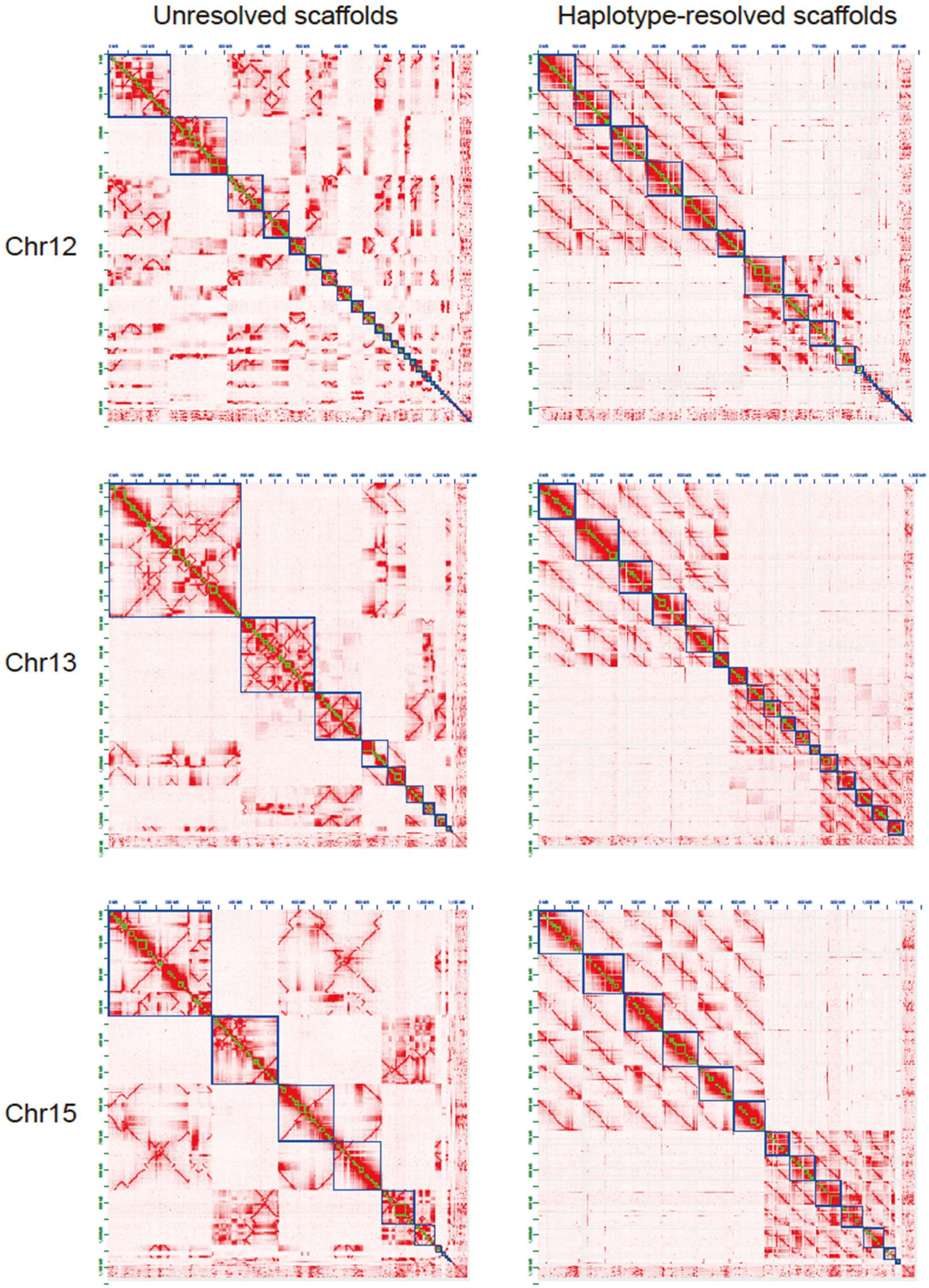
Unresolved scaffolds
Haplotype-resolved scaffolds
Chr12
Chr13
Chr15

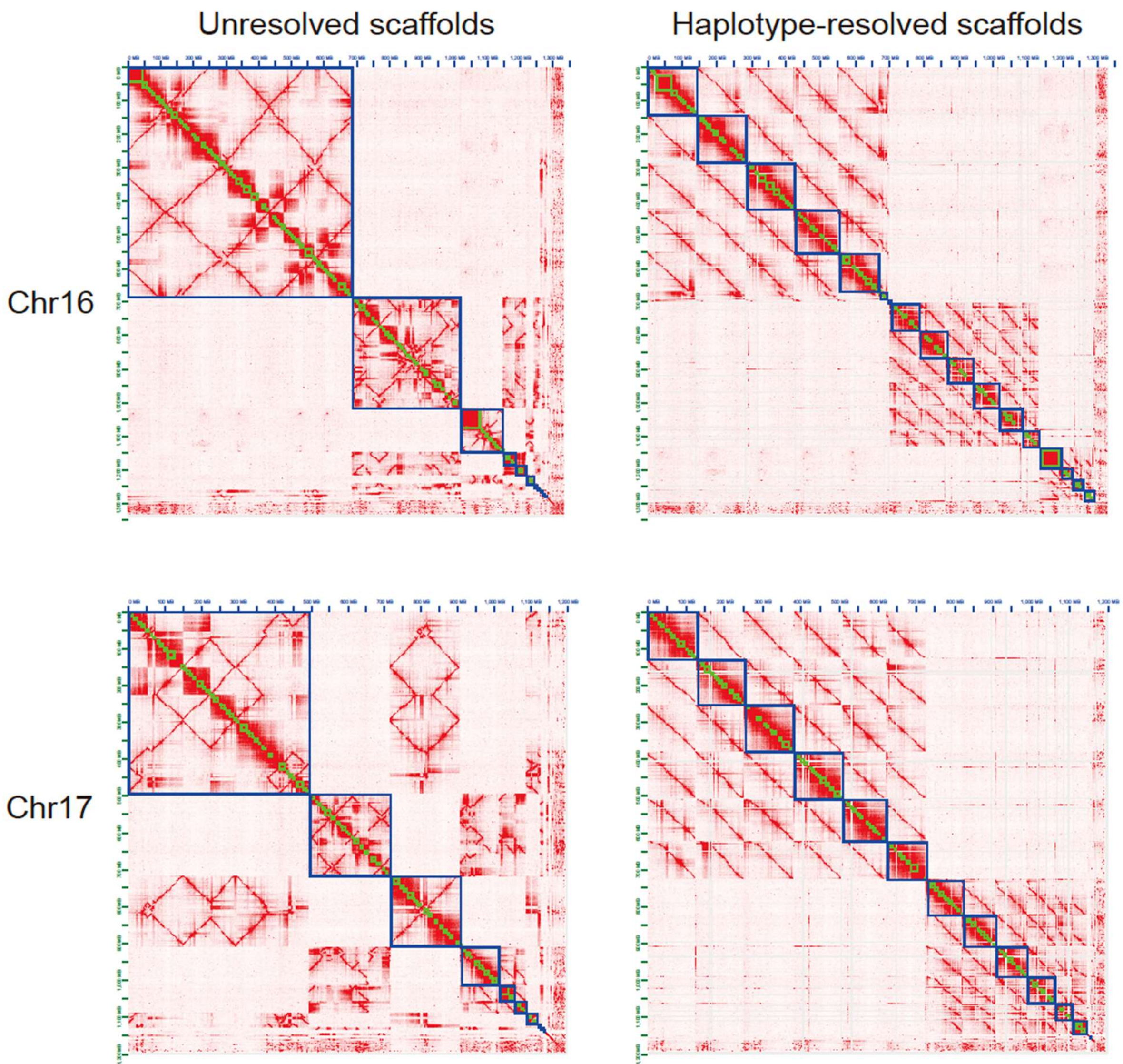

Supplemental Figure 4 First-round Hi-C scaffolding of 8 groups of homologous chromosome-fragments for hexaploid *H. tuberosus*. The contigs and mapped Hi-C reads of each homologous group were generated according to the contig alignments to the chromosome-level reference genome of *H. annuus*, and for each group, the unresolved scaffolding results (on the left) were produced using YaHS and the haplotype-resolved scaffolding results (on the right) were generated by manual curation in JuiceBox. In the Hi-C contact heatmap of unresolved and haplotype-resolved scaffolds, green and blue squares indicate contigs and scaffolds, respectively, and the color darkness of each pixel is proportional to the Hi-C contact density between the two corresponding genomic regions.

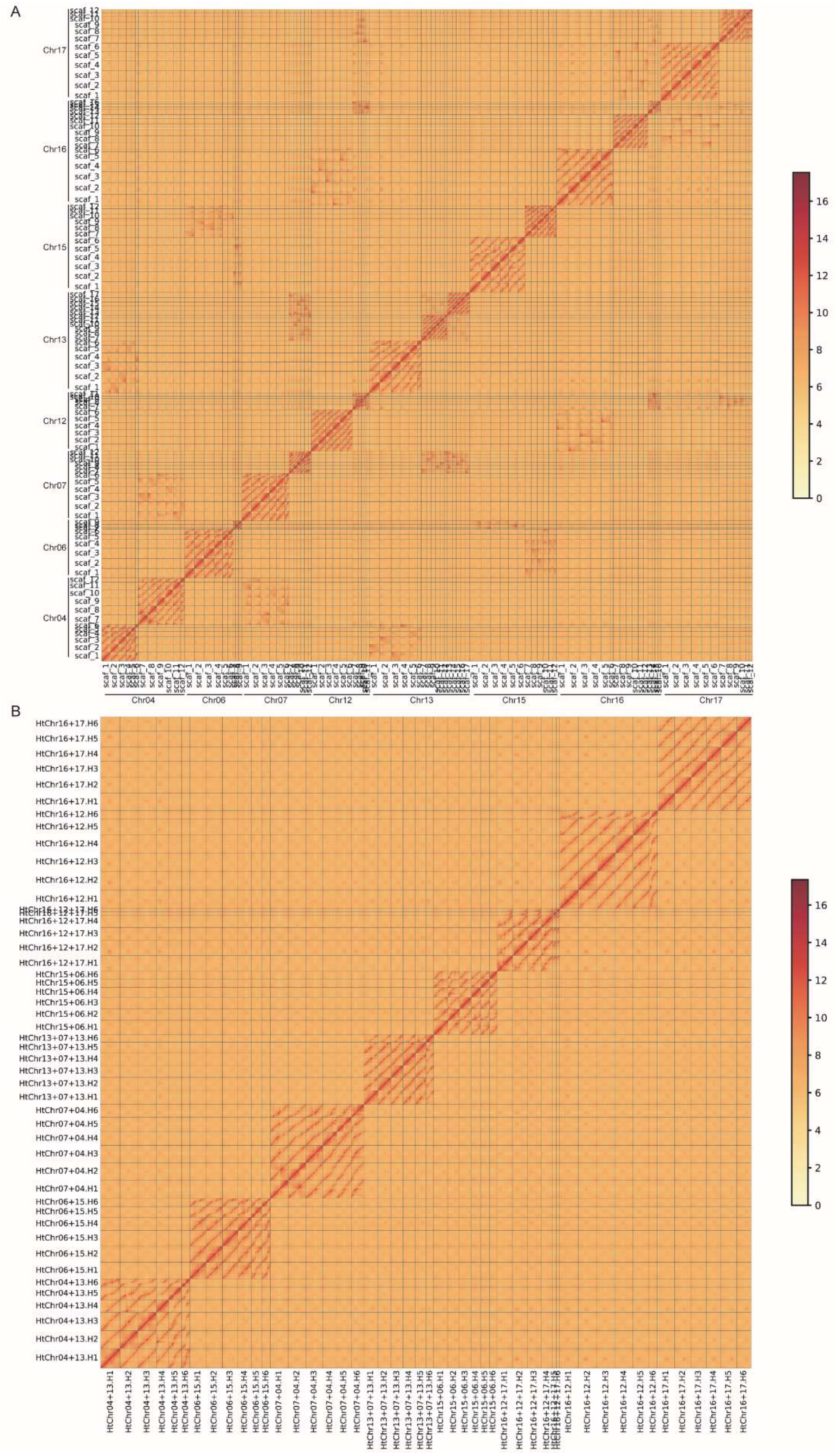

Supplemental Figure 5 First-round Hi-C contact heatmap of 8 groups of homologous chromosome-fragments (a) and re-joined pseudochromosomes (b) for hexaploid *H. tuberosus*. The chromosome-fragment-level scaffolds were obtained by manual curation as shown in Supplemental Figure 4, and whole-genome Hi-C reads were mapped

to these scaffolds to find the missing Hi-C links among the scaffolds from different homologous groups. Based on the reliable Hi-C links among non-homologous scaffolds, we used in-house scripts to generate the 8 groups of pseudochromosomes. Each bin in the heatmap represents 5-Mb genomic region and its color is proportional to the Log2-transformated Hi-C links between two 5-Mb bins or within one 5-Mb bin.

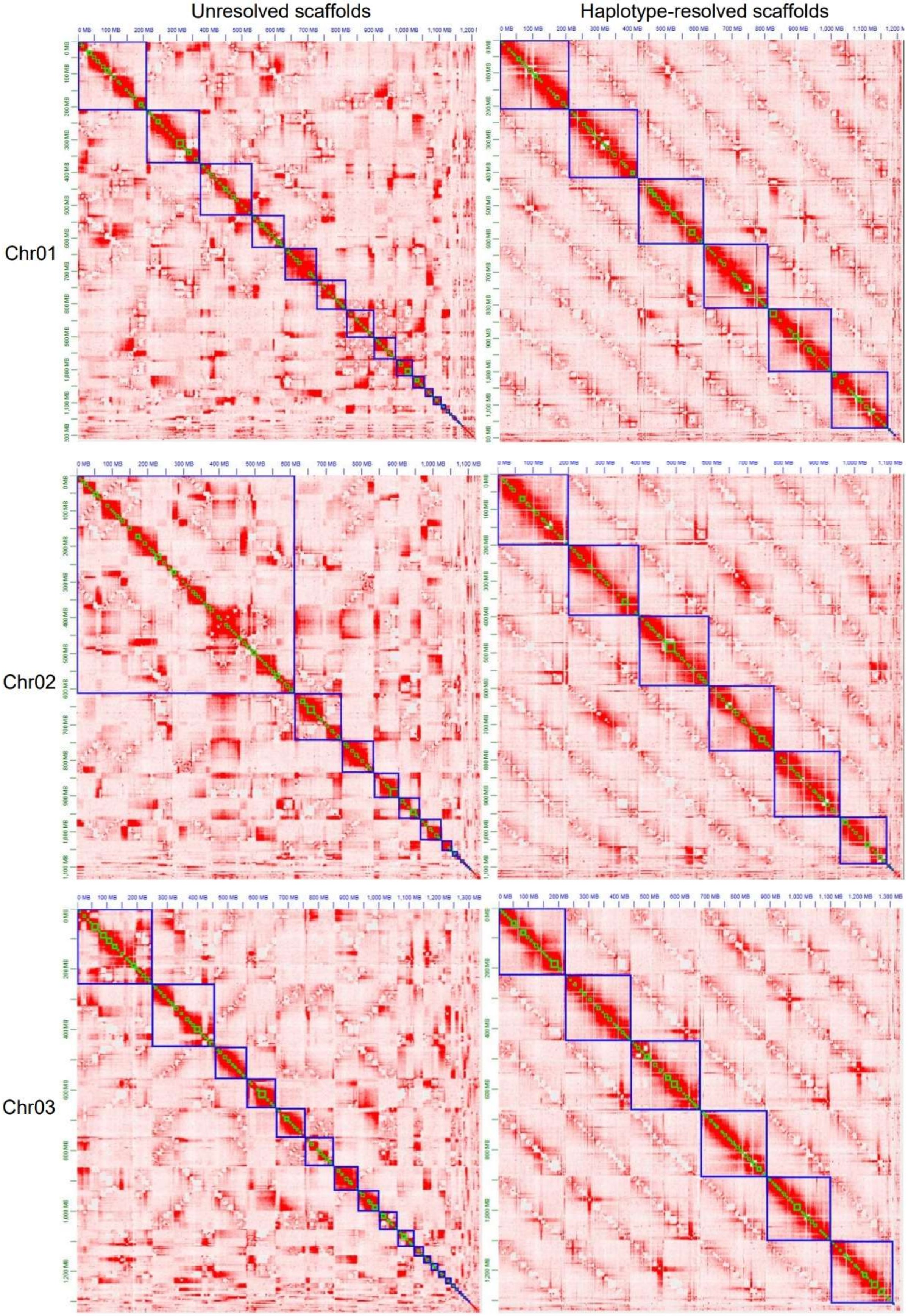

Unresolved scaffolds
Haplotype-resolved scaffolds
Chr01
Chr02
Chr03

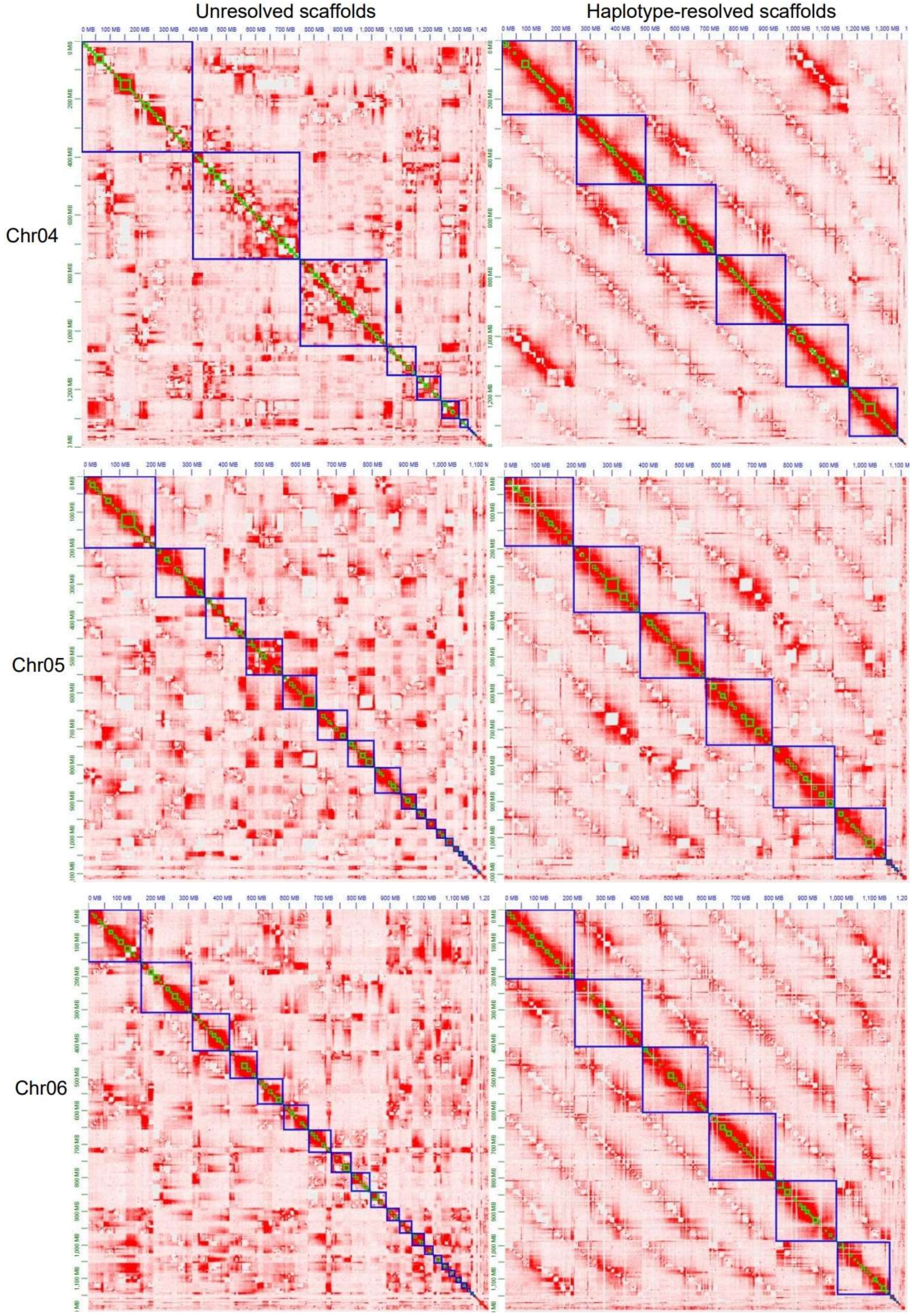
Unresolved scaffolds
Haplotype-resolved scaffolds
Chr04
Chr05
Chr06

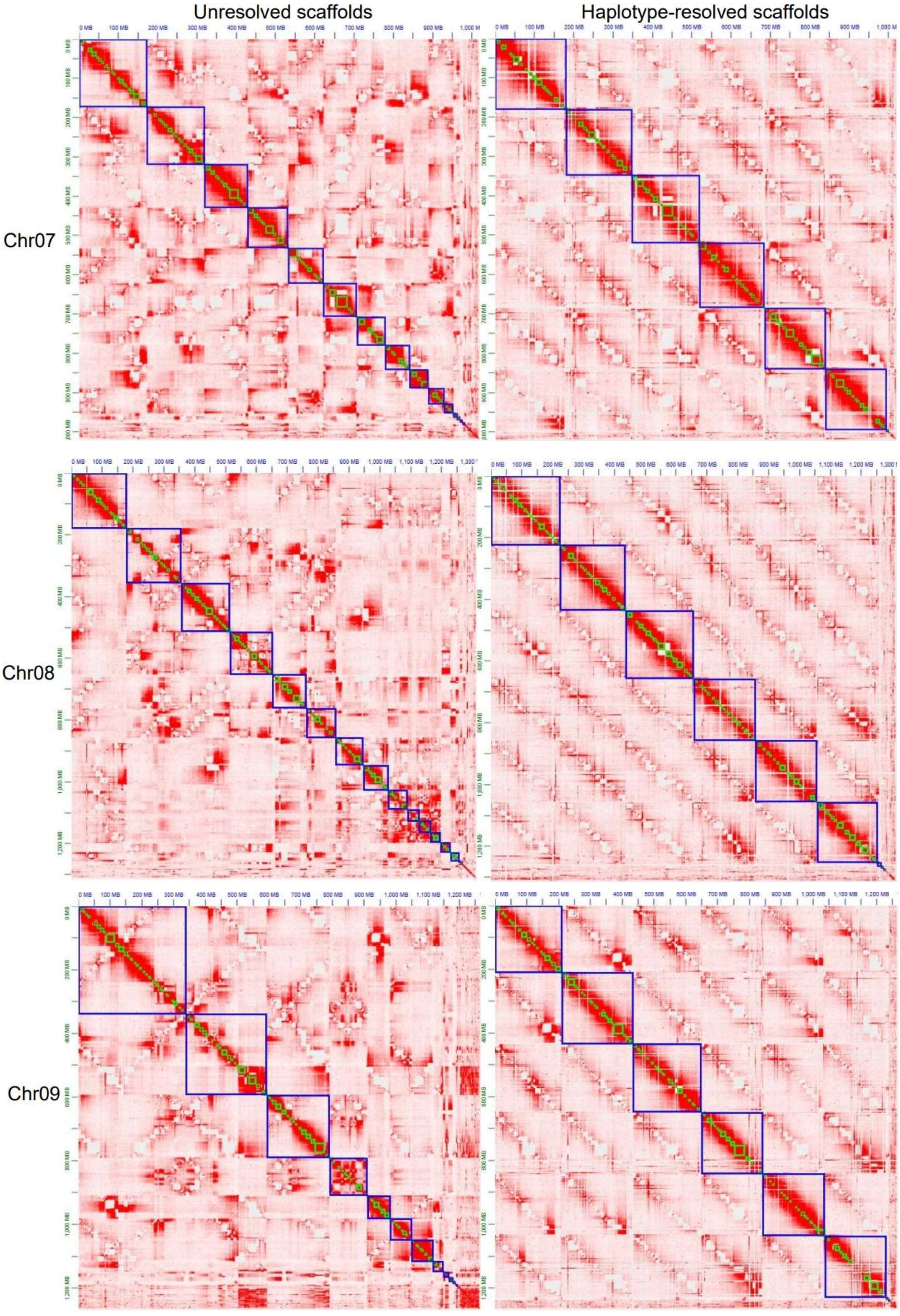
Unresolved scaffolds
Haplotype-resolved scaffolds
Chr07
Chr08
Chr09

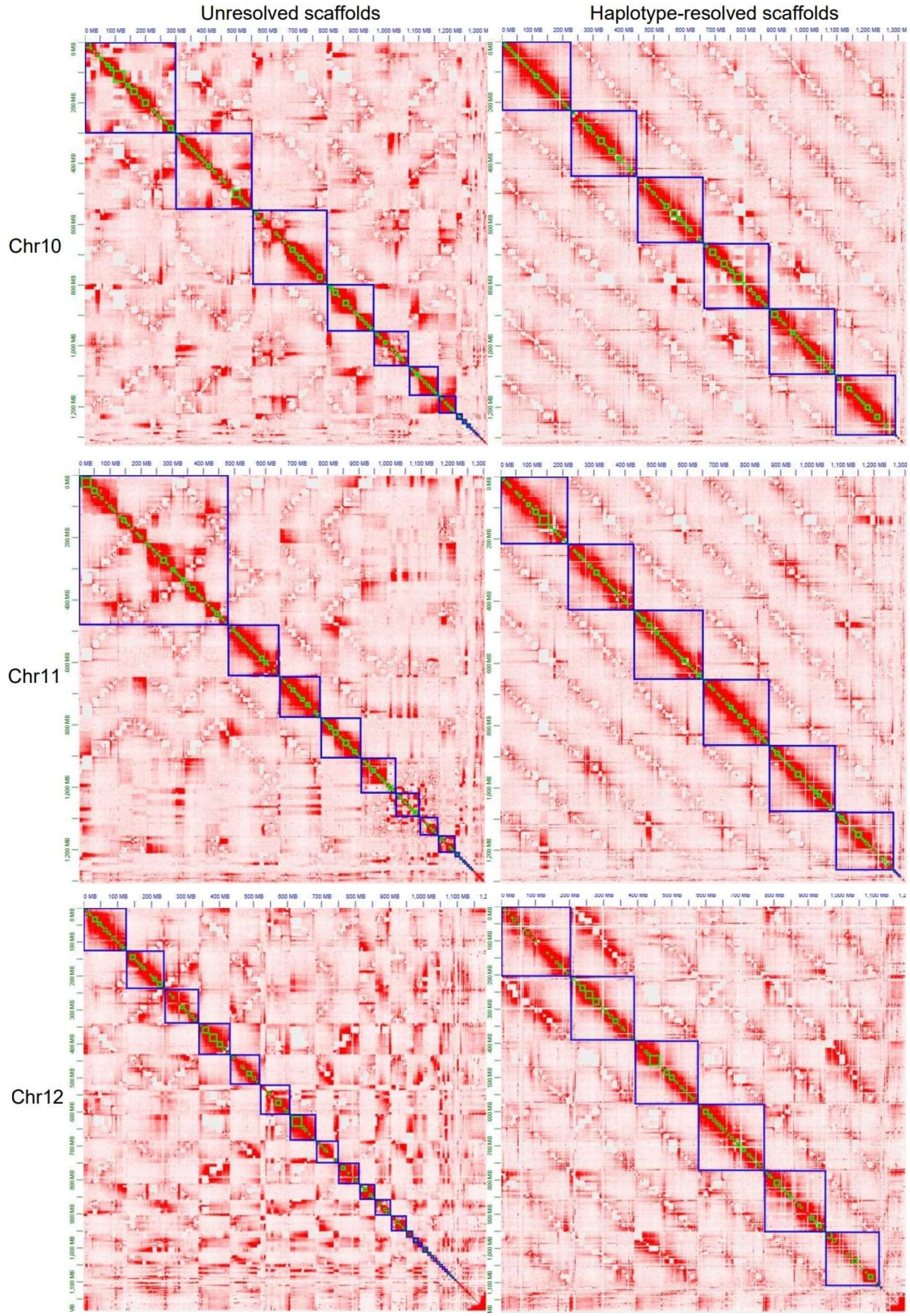
Unresolved scaffolds
Haplotype-resolved scaffolds
Chr10
Chr11
Chr12

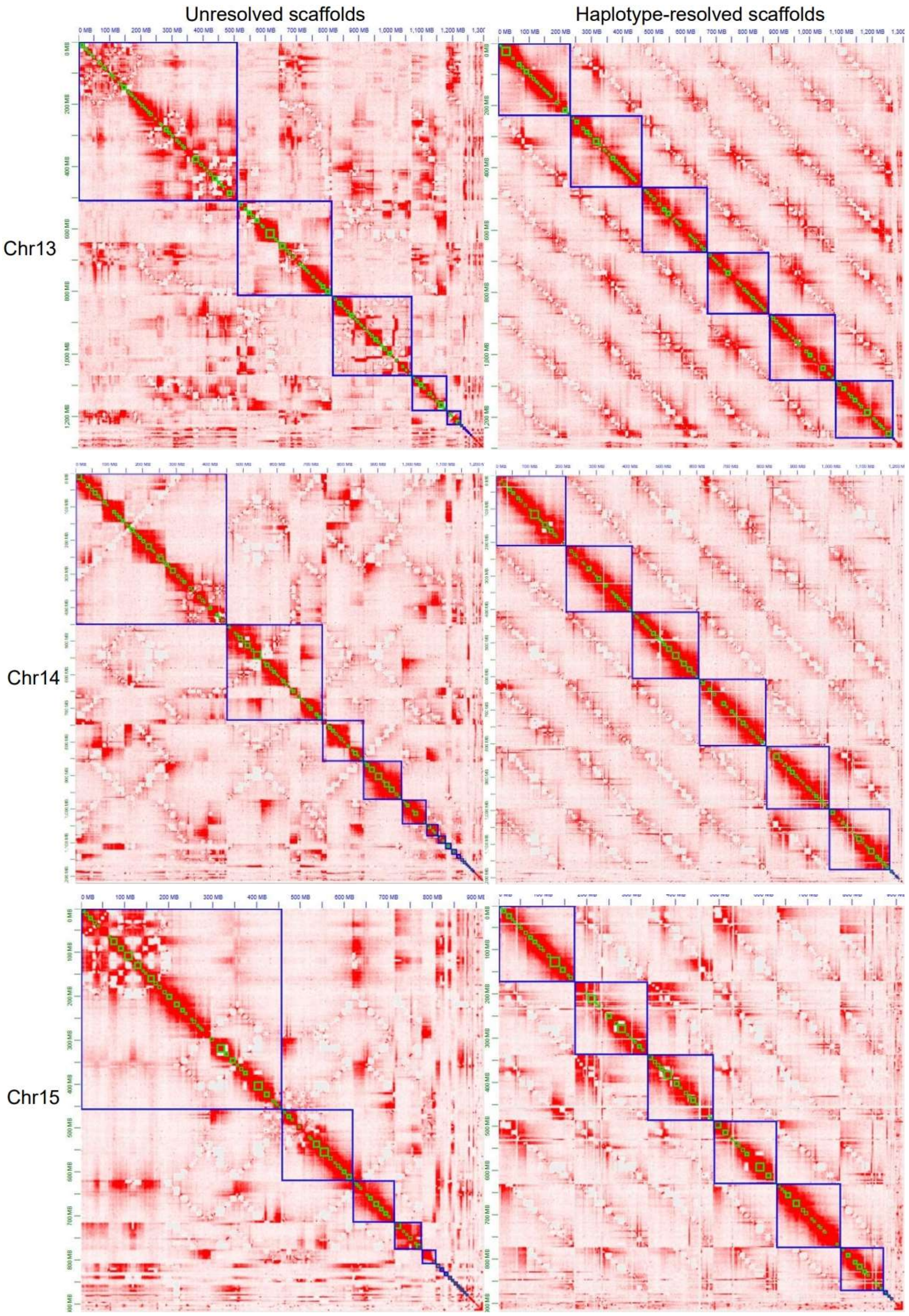

Unresolved scaffolds
Haplotype-resolved scaffolds
Chr13
Chr14
Chr15

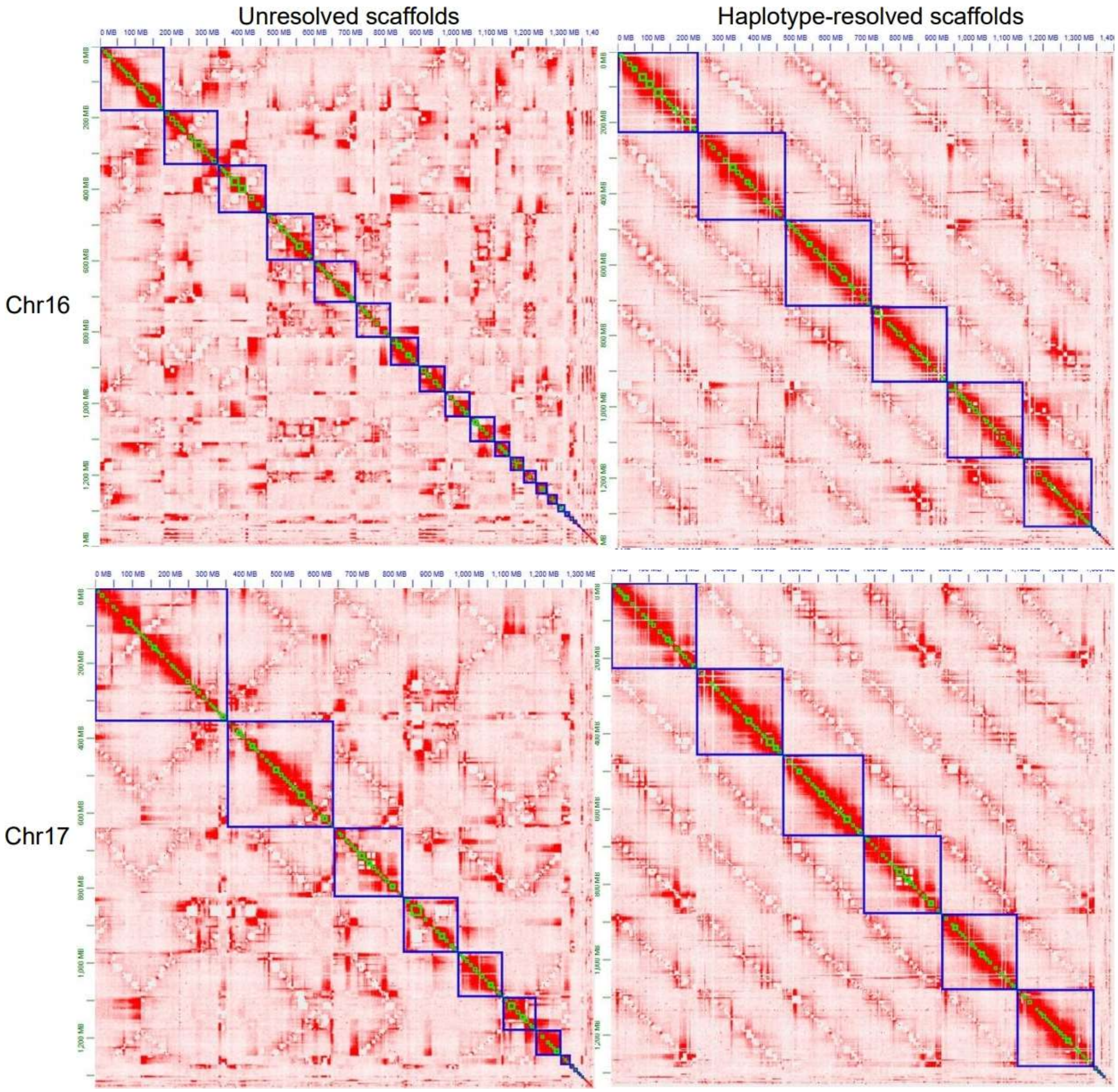

Supplemental Figure 6 Second-round Hi-C scaffolding of 17 groups of homologous chromosomes for hexaploid *H. tuberosus*. The contigs and mapped Hi-C reads of each homologous group were generated according to the contig alignments to the 17 chromosomes of pseudo-monoploid genome of *H. tuberosus*. For each group, the unresolved scaffolding results (on the left) were produced using YaHS and the haplotype-resolved scaffolding results (on the right) were generated by manual curation in JuiceBox. In the Hi-C contact heatmap of unresolved and haplotype-resolved scaffolds, green and blue squares indicate contigs and scaffolds, respectively, and the color darkness of each pixel is proportional to the Hi-C contact density between the two corresponding genomic regions.

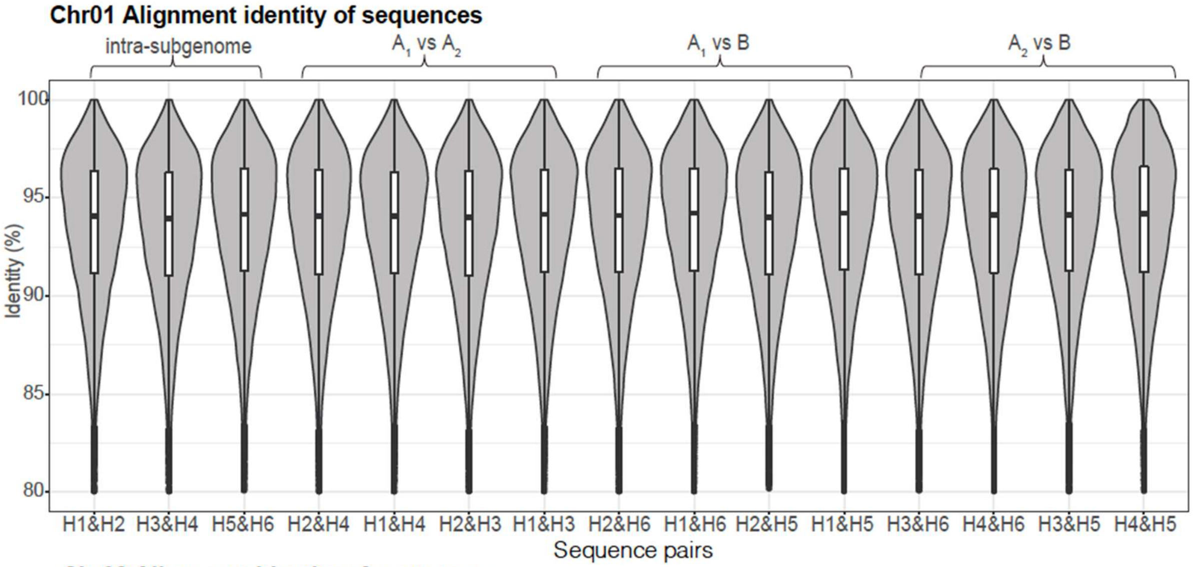
Chr01 Alignment identity of sequences
intra-subgenome
A_1 vs A_2
A_1 vs B
A_2 vs B
Identity (%)
100
95
90
85
80
H1&H2 H3&H4 H5&H6 H2&H4 H1&H4 H2&H3 H1&H3 H2&H6 H1&H6 H2&H5 H1&H5 H3&H6 H4&H6 H3&H5 H4&H5
Sequence pairs

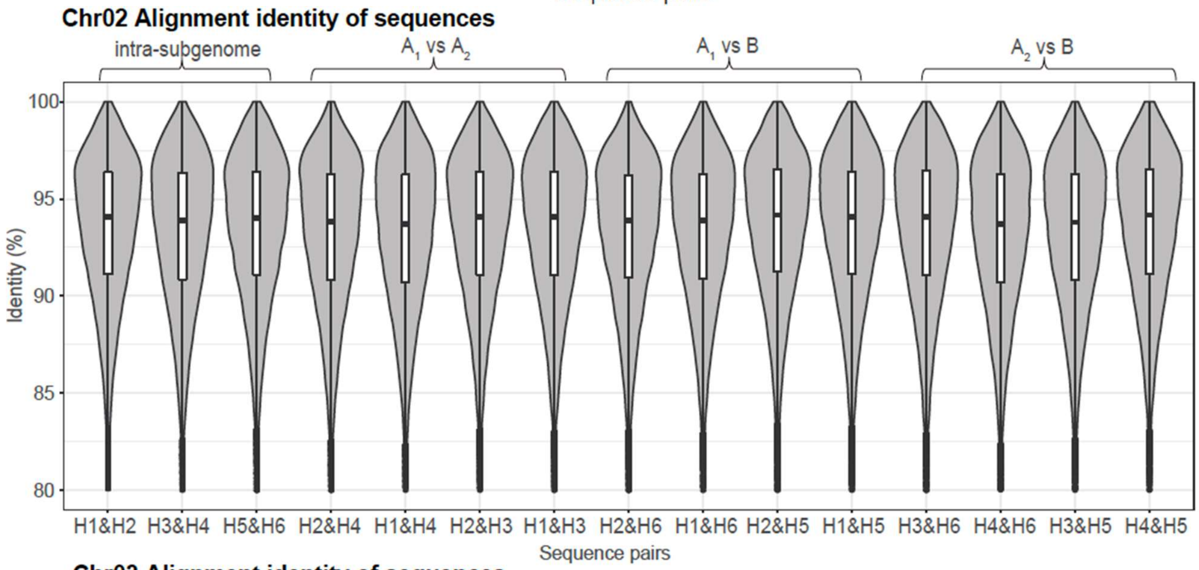
Chr02 Alignment identity of sequences
intra-subgenome
A_1 vs A_2
A_1 vs B
A_2 vs B
Identity (%)
100
95
90
85
80
H1&H2 H3&H4 H5&H6 H2&H4 H1&H4 H2&H3 H1&H3 H2&H6 H1&H6 H2&H5 H1&H5 H3&H6 H4&H6 H3&H5 H4&H5
Sequence pairs

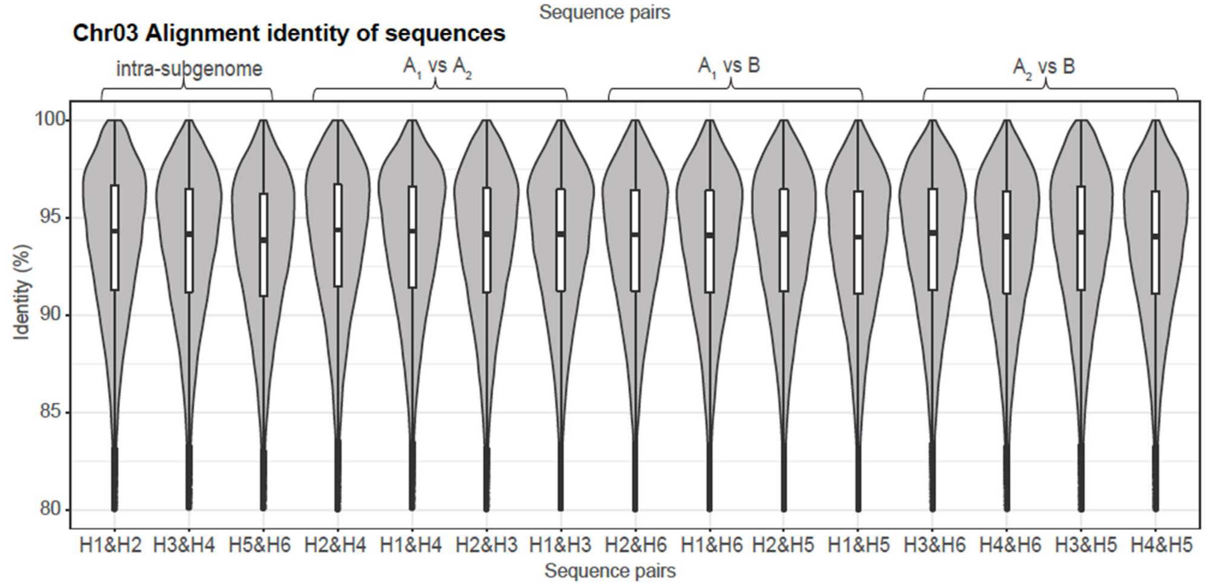
Chr03 Alignment identity of sequences
intra-subgenome
A_1 vs A_2
A_1 vs B
A_2 vs B
Identity (%)
100
95
90
85
80
H1&H2 H3&H4 H5&H6 H2&H4 H1&H4 H2&H3 H1&H3 H2&H6 H1&H6 H2&H5 H1&H5 H3&H6 H4&H6 H3&H5 H4&H5
Sequence pairs

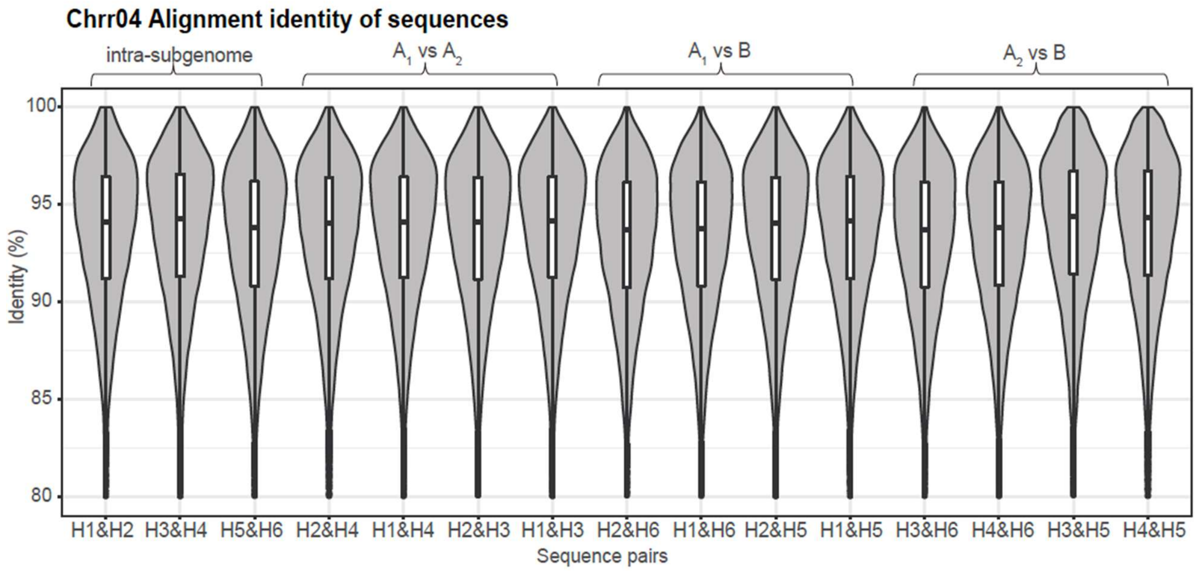
Chrr04 Alignment identity of sequences
intra-subgenome
A1 vs A2
A1 vs B
A2 vs B
Identity (%)
100
95
90
85
80
H1&H2 H3&H4 H5&H6 H2&H4 H1&H4 H2&H3 H1&H3 H2&H6 H1&H6 H2&H5 H1&H5 H3&H6 H4&H6 H3&H5 H4&H5
Sequence pairs

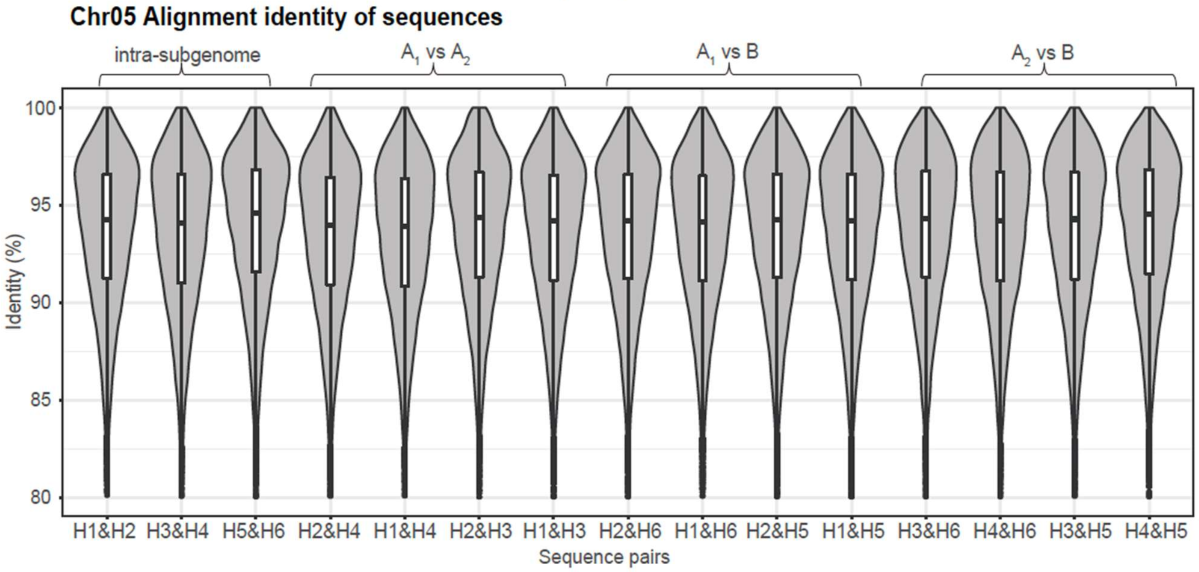
Chr05 Alignment identity of sequences
intra-subgenome
A1 vs A2
A1 vs B
A2 vs B
Identity (%)
100
95
90
85
80
H1&H2 H3&H4 H5&H6 H2&H4 H1&H4 H2&H3 H1&H3 H2&H6 H1&H6 H2&H5 H1&H5 H3&H6 H4&H6 H3&H5 H4&H5
Sequence pairs

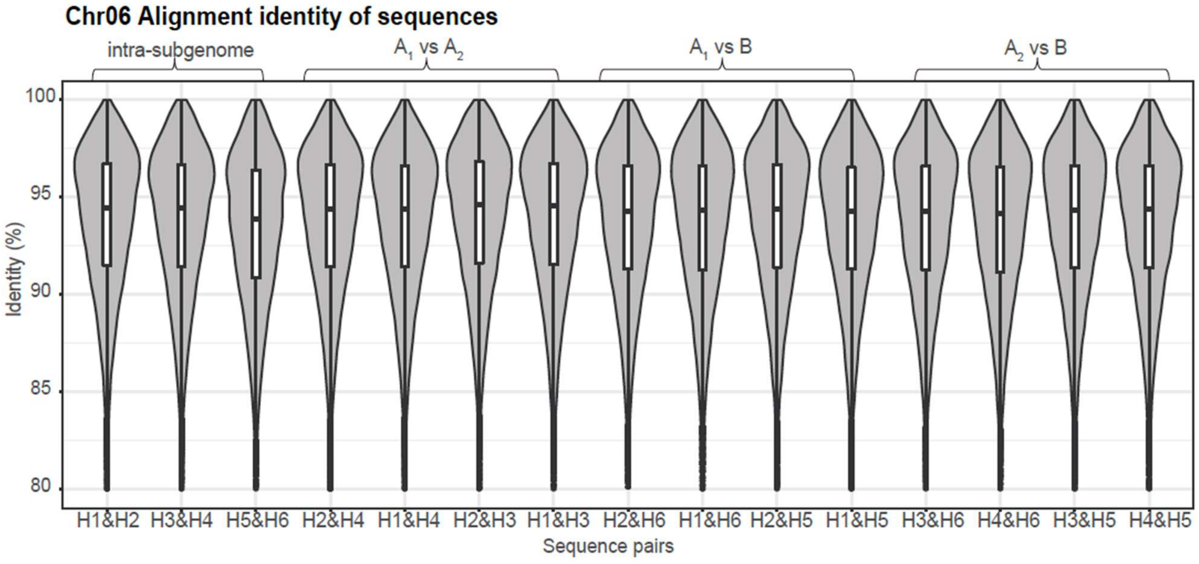
Chr06 Alignment identity of sequences
intra-subgenome
A1 vs A2
A1 vs B
A2 vs B
Identity (%)
100
95
90
85
80
H1&H2 H3&H4 H5&H6 H2&H4 H1&H4 H2&H3 H1&H3 H2&H6 H1&H6 H2&H5 H1&H5 H3&H6 H4&H6 H3&H5 H4&H5
Sequence pairs

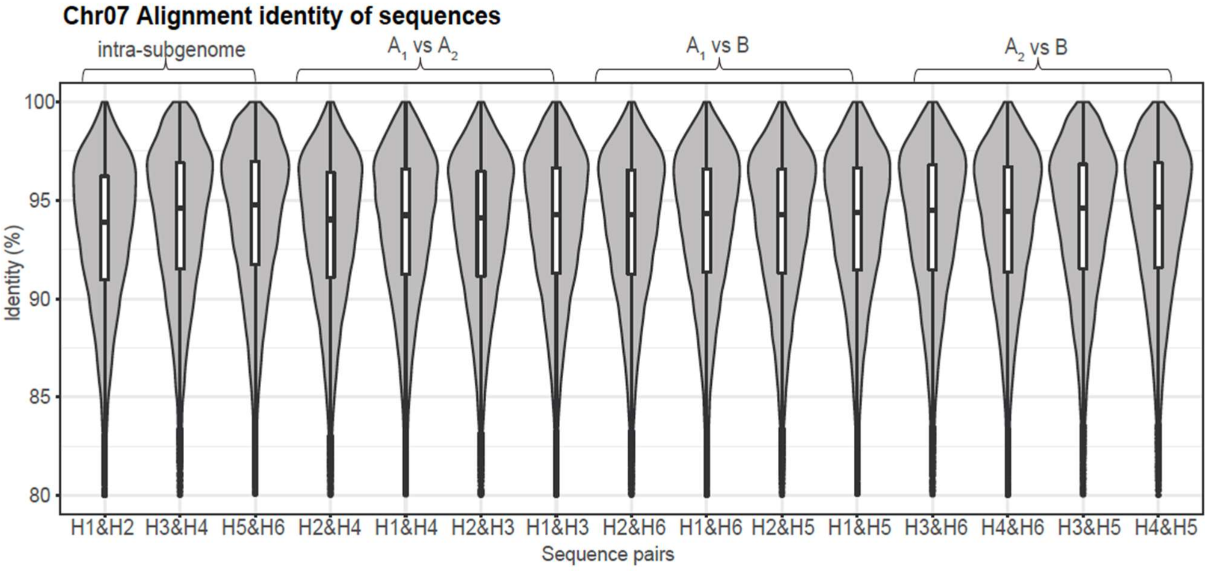
Chr07 Alignment identity of sequences
intra-subgenome
A1 vs A2
A1 vs B
A2 vs B
Identity (%)
100
95
90
85
80
H1&H2 H3&H4 H5&H6 H2&H4 H1&H4 H2&H3 H1&H3 H2&H6 H1&H6 H2&H5 H1&H5 H3&H6 H4&H6 H3&H5 H4&H5
Sequence pairs

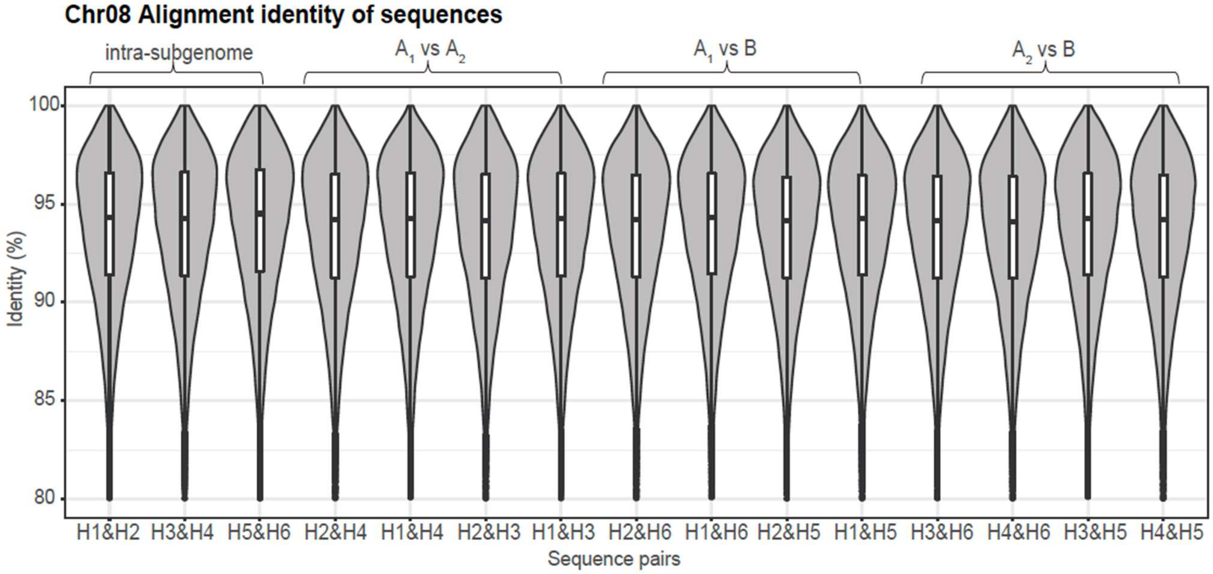
Chr08 Alignment identity of sequences
intra-subgenome
A1 vs A2
A1 vs B
A2 vs B
Identity (%)
100
95
90
85
80
H1&H2 H3&H4 H5&H6 H2&H4 H1&H4 H2&H3 H1&H3 H2&H6 H1&H6 H2&H5 H1&H5 H3&H6 H4&H6 H3&H5 H4&H5
Sequence pairs

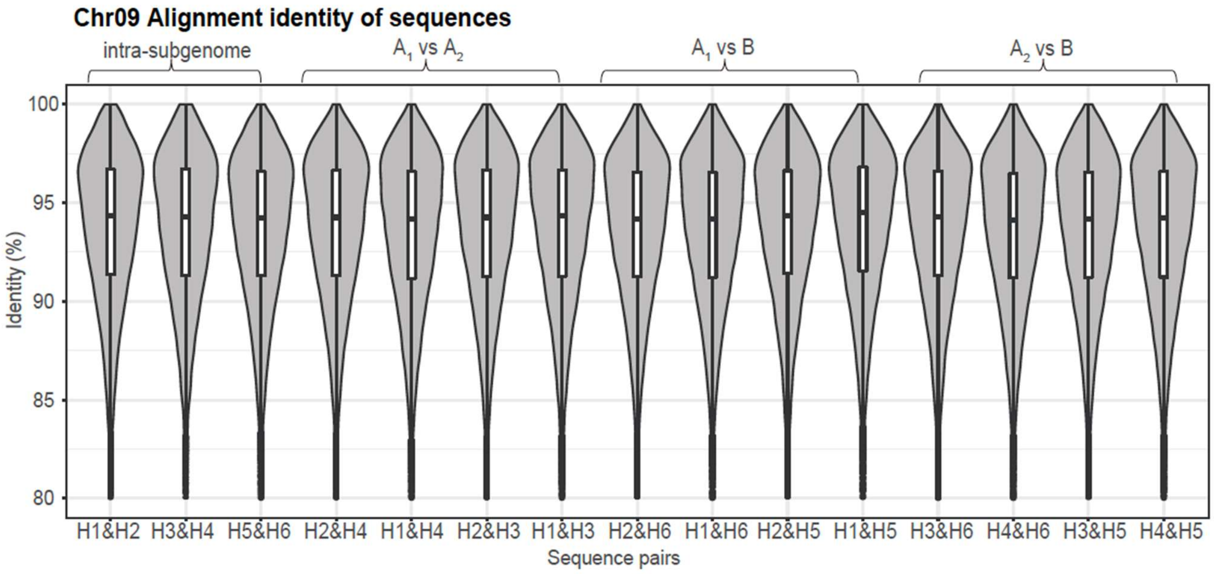
Chr09 Alignment identity of sequences
intra-subgenome
A1 vs A2
A1 vs B
A2 vs B
Identity (%)
100
95
90
85
80
H1&H2 H3&H4 H5&H6 H2&H4 H1&H4 H2&H3 H1&H3 H2&H6 H1&H6 H2&H5 H1&H5 H3&H6 H4&H6 H3&H5 H4&H5
Sequence pairs

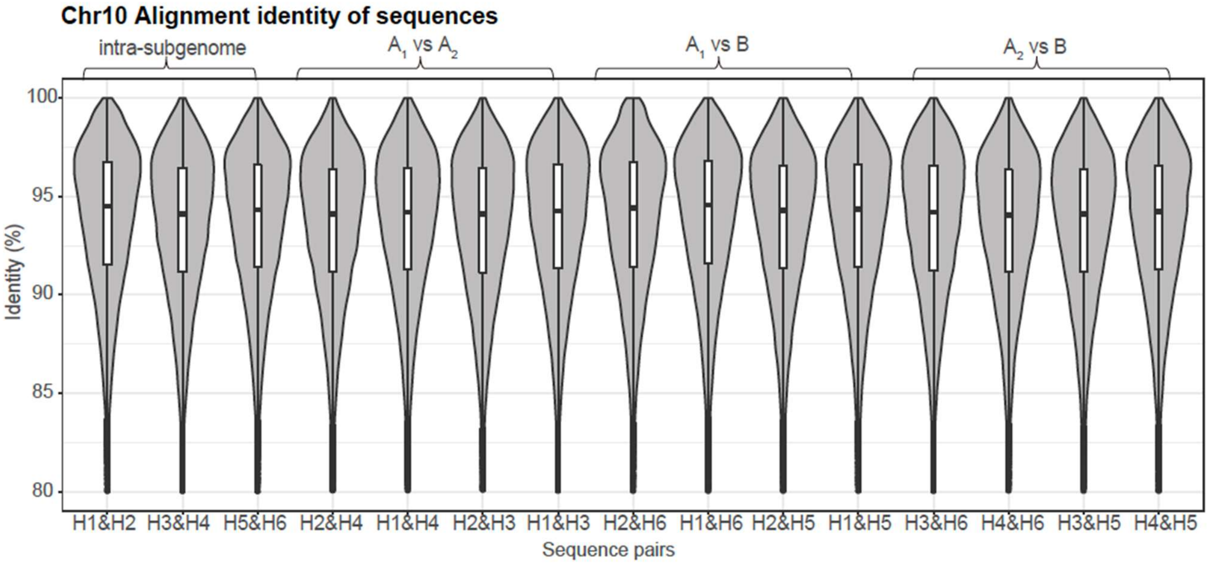
Chr10 Alignment identity of sequences
intra-subgenome
A_1 vs A_2
A_1 vs B
A_2 vs B
Identity (%)
100
95
90
85
80
H1&H2 H3&H4 H5&H6 H2&H4 H1&H4 H2&H3 H1&H3 H2&H6 H1&H6 H2&H5 H1&H5 H3&H6 H4&H6 H3&H5 H4&H5
Sequence pairs

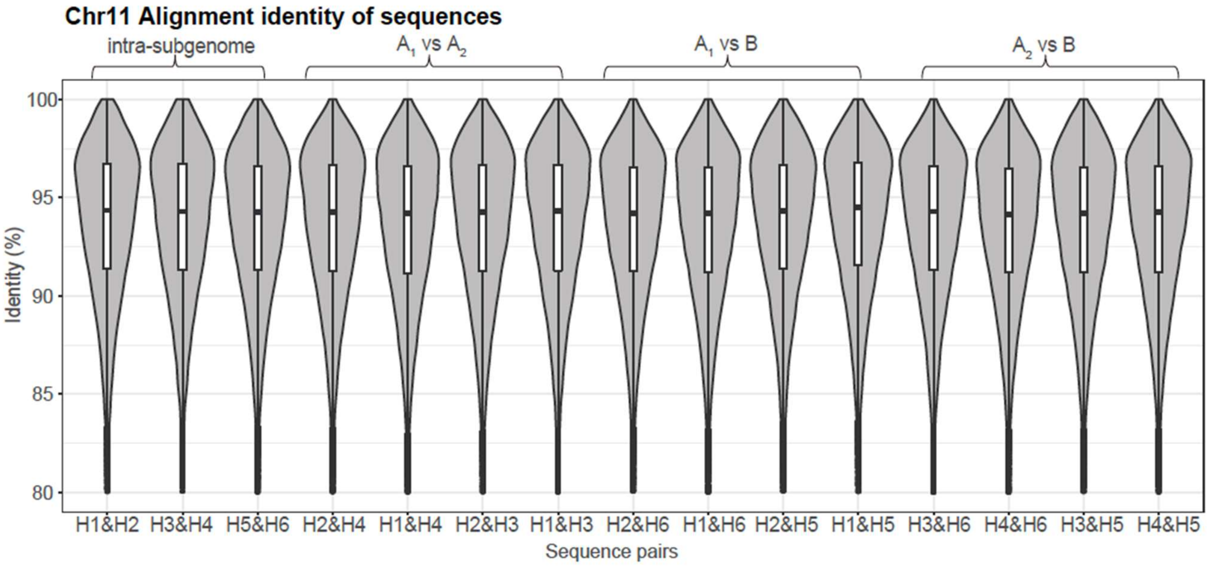
Chr11 Alignment identity of sequences
intra-subgenome
A_1 vs A_2
A_1 vs B
A_2 vs B
Identity (%)
100
95
90
85
80
H1&H2 H3&H4 H5&H6 H2&H4 H1&H4 H2&H3 H1&H3 H2&H6 H1&H6 H2&H5 H1&H5 H3&H6 H4&H6 H3&H5 H4&H5
Sequence pairs

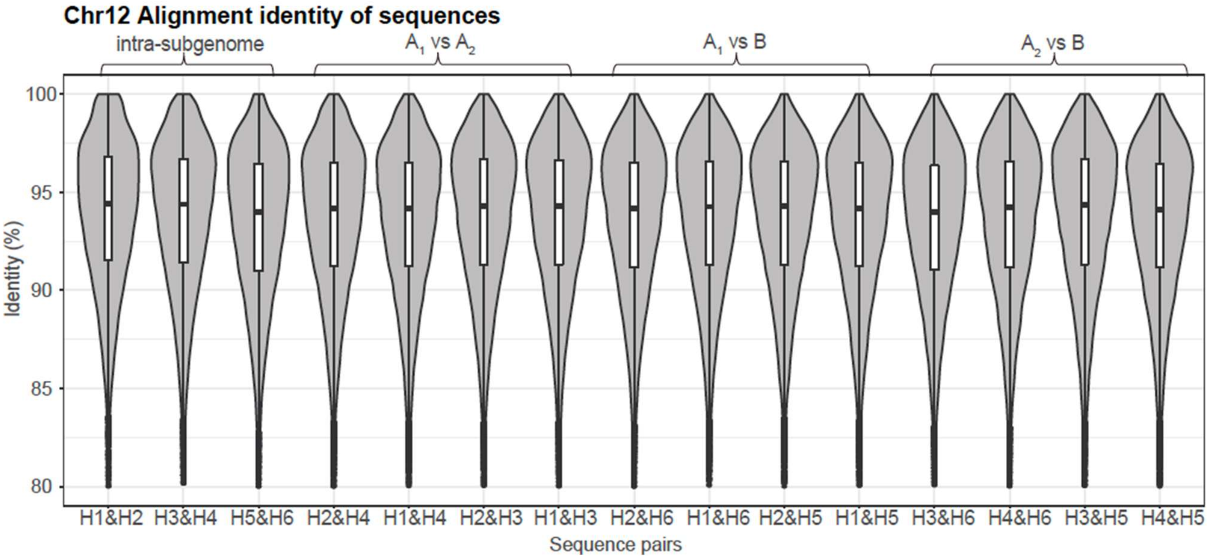
Chr12 Alignment identity of sequences
intra-subgenome
A_1 vs A_2
A_1 vs B
A_2 vs B
Identity (%)
100
95
90
85
80
H1&H2 H3&H4 H5&H6 H2&H4 H1&H4 H2&H3 H1&H3 H2&H6 H1&H6 H2&H5 H1&H5 H3&H6 H4&H6 H3&H5 H4&H5
Sequence pairs

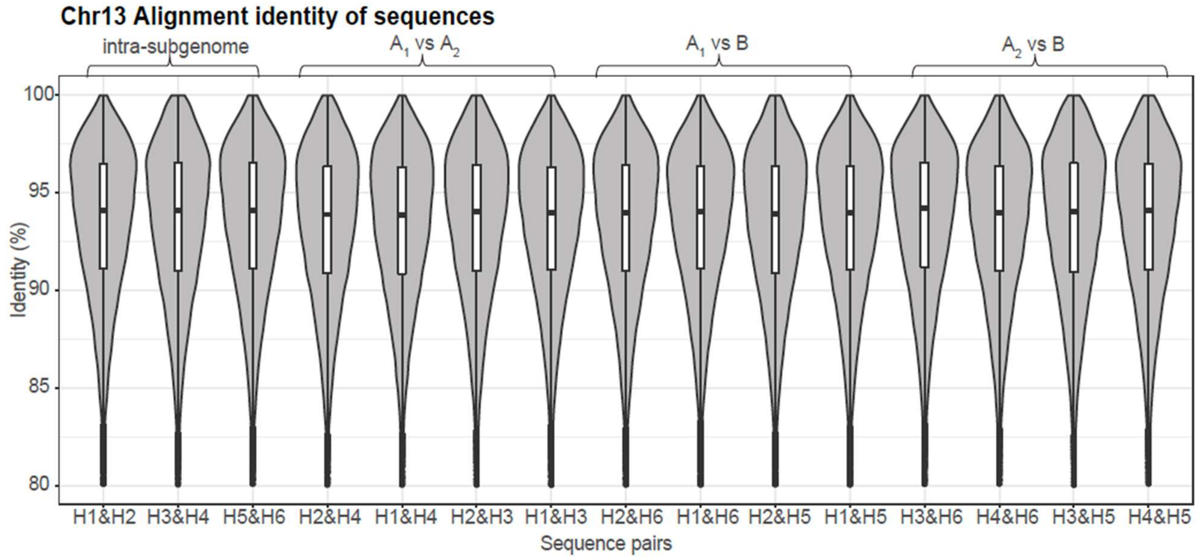
Chr13 Alignment identity of sequences
intra-subgenome
A1 vs A2
A1 vs B
A2 vs B
Identity (%)
100
95
90
85
80
H1&H2 H3&H4 H5&H6 H2&H4 H1&H4 H2&H3 H1&H3 H2&H6 H1&H6 H2&H5 H1&H5 H3&H6 H4&H6 H3&H5 H4&H5
Sequence pairs

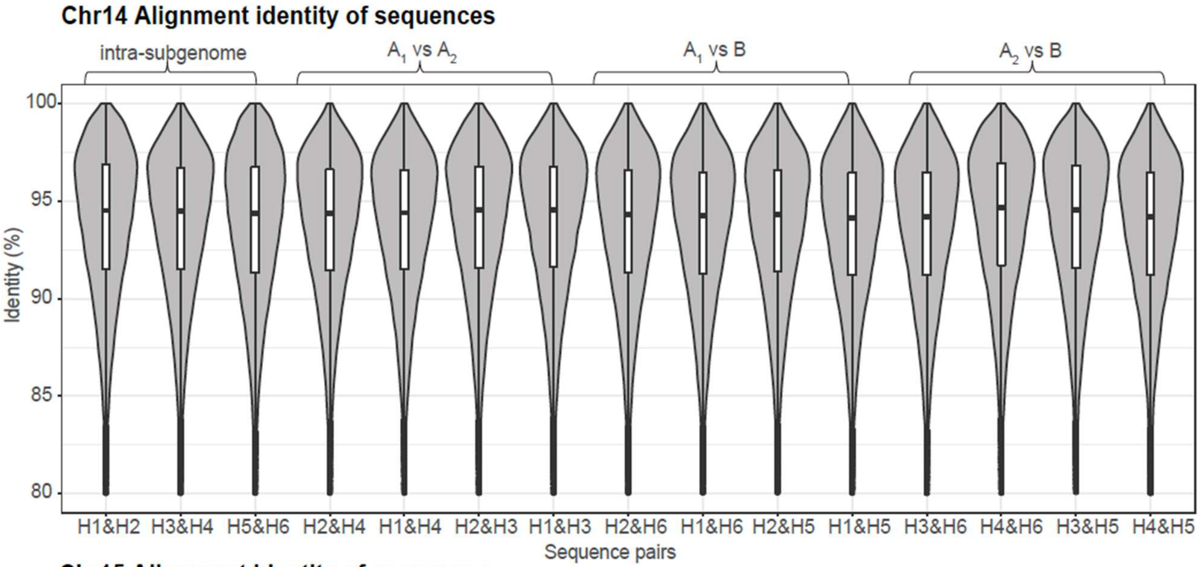
Chr14 Alignment identity of sequences
intra-subgenome
A1 vs A2
A1 vs B
A2 vs B
Identity (%)
100
95
90
85
80
H1&H2 H3&H4 H5&H6 H2&H4 H1&H4 H2&H3 H1&H3 H2&H6 H1&H6 H2&H5 H1&H5 H3&H6 H4&H6 H3&H5 H4&H5
Sequence pairs

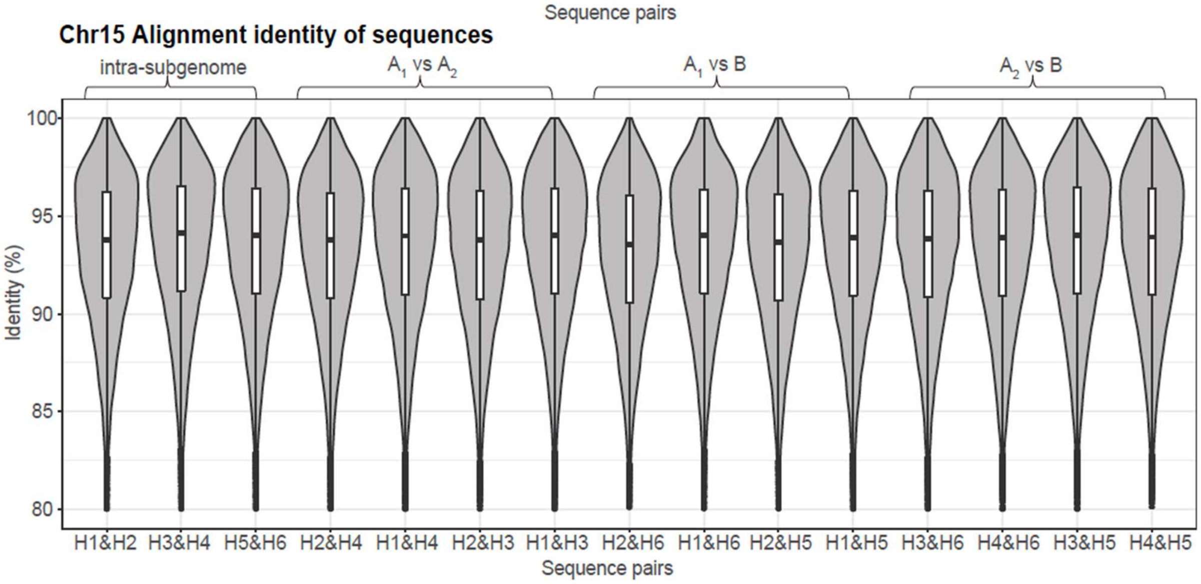
Chr15 Alignment identity of sequences
intra-subgenome
A1 vs A2
A1 vs B
A2 vs B
Identity (%)
100
95
90
85
80
H1&H2 H3&H4 H5&H6 H2&H4 H1&H4 H2&H3 H1&H3 H2&H6 H1&H6 H2&H5 H1&H5 H3&H6 H4&H6 H3&H5 H4&H5
Sequence pairs

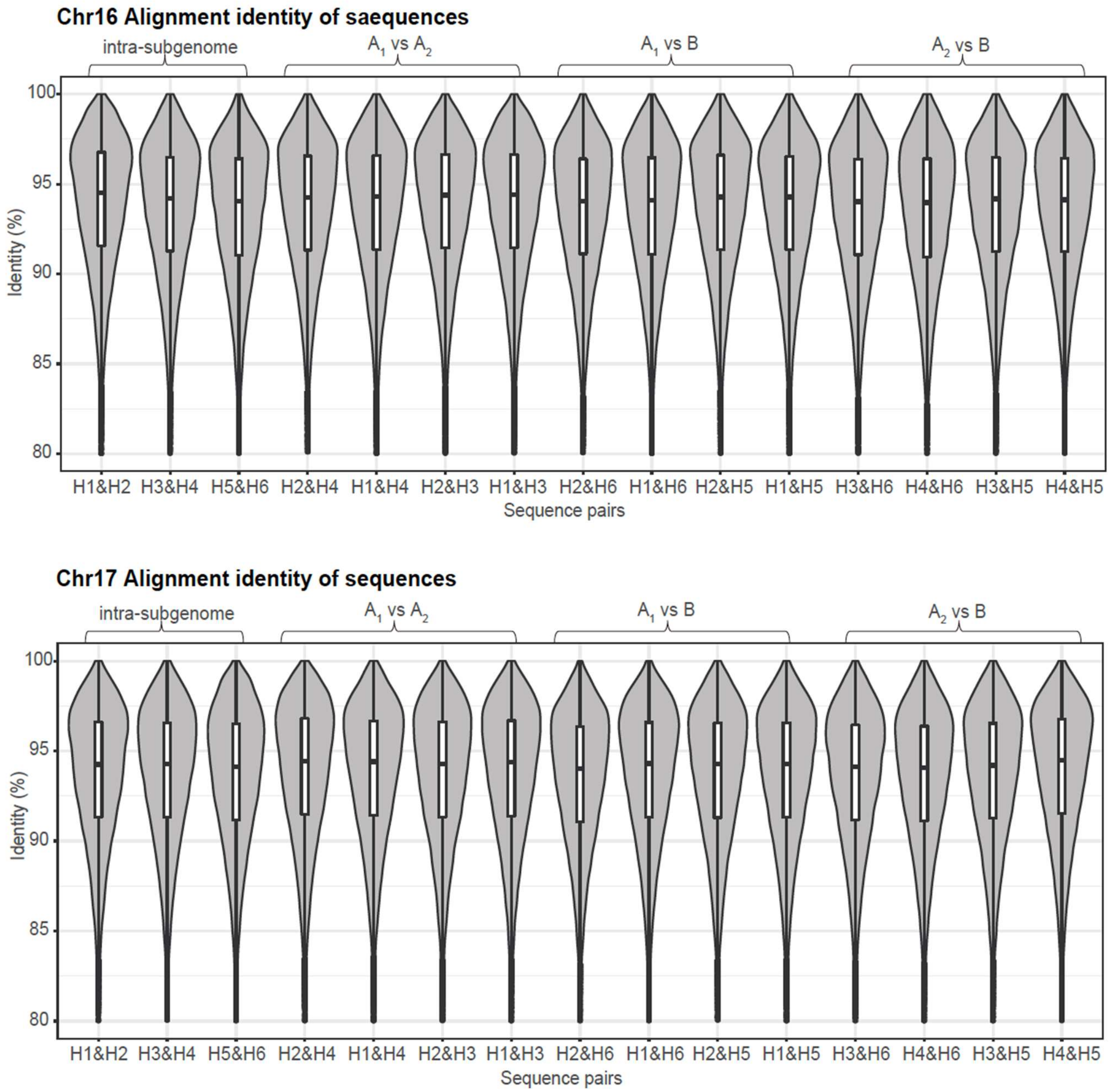

Supplemental Figure 7 Violin and box plot of alignment identities of homologous sequences from 17 homologous chromosomes for hexaploid *H. tuberosus*. For each homologous group, the 1-to-1 DNA sequence alignment blocks between any two homologous chromosomes were identified using MUMmer4, and their identities were used to infer whether these two chromosomes come from the same or different sub-genomes. Solid circles refer to outliers and horizontal bold lines in box plots indicate medians.

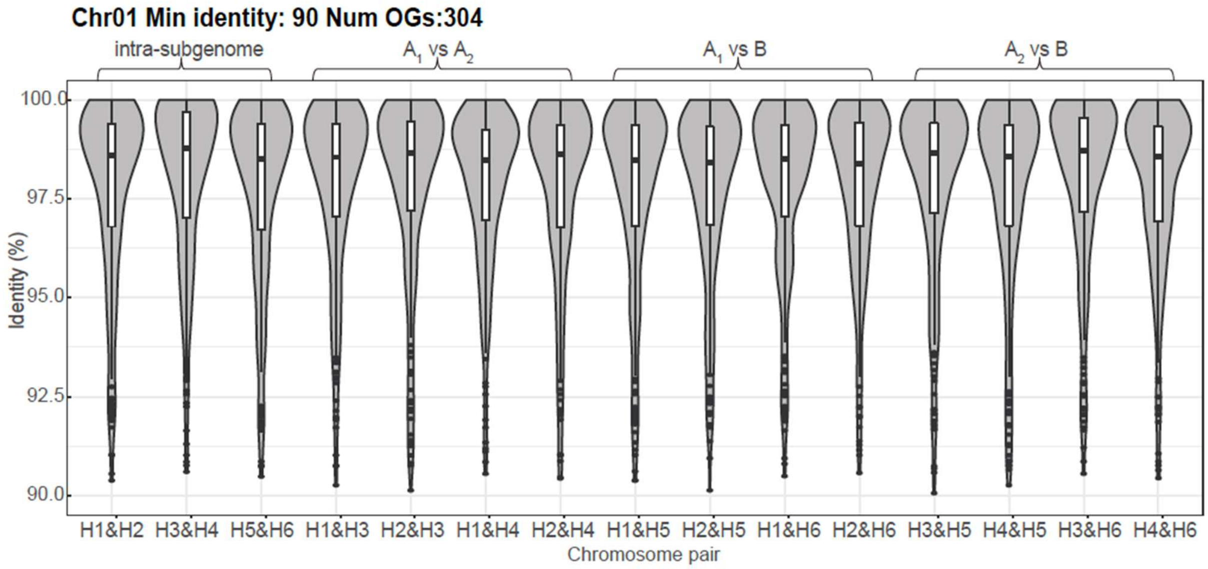
Chr01 Min identity: 90 Num OGs:304
intra-subgenome
A1 vs A2
A1 vs B
A2 vs B
Identity (%)
100.0
97.5
95.0
92.5
90.0
H1&H2 H3&H4 H5&H6 H1&H3 H2&H3 H1&H4 H2&H4 H1&H5 H2&H5 H1&H6 H2&H6 H3&H5 H4&H5 H3&H6 H4&H6
Chromosome pair

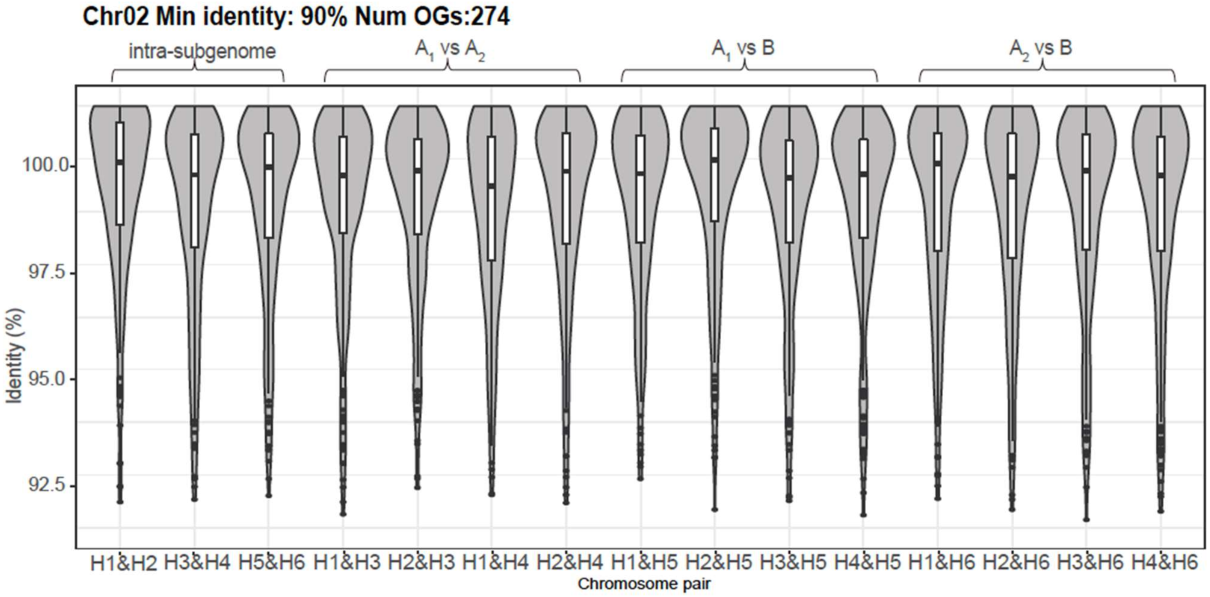
Chr02 Min identity: 90% Num OGs:274
intra-subgenome
A1 vs A2
A1 vs B
A2 vs B
Identity (%)
100.0
97.5
95.0
92.5
H1&H2 H3&H4 H5&H6 H1&H3 H2&H3 H1&H4 H2&H4 H1&H5 H2&H5 H3&H5 H4&H5 H1&H6 H2&H6 H3&H6 H4&H6
Chromosome pair

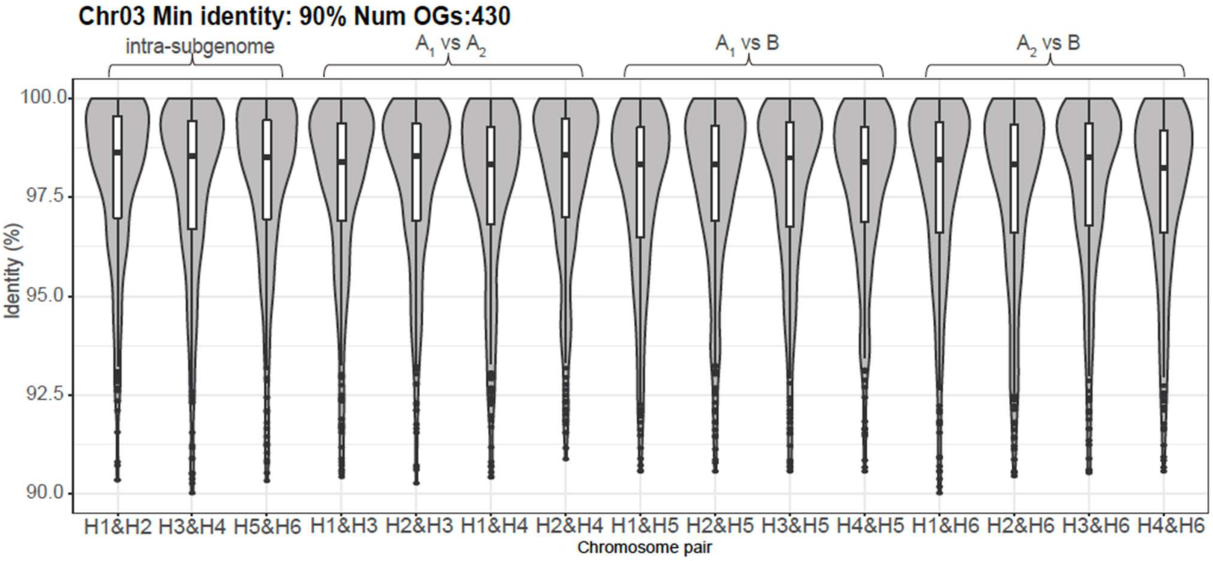
Chr03 Min identity: 90% Num OGs:430
intra-subgenome
A1 vs A2
A1 vs B
A2 vs B
Identity (%)
100.0
97.5
95.0
92.5
90.0
H1&H2 H3&H4 H5&H6 H1&H3 H2&H3 H1&H4 H2&H4 H1&H5 H2&H5 H3&H5 H4&H5 H1&H6 H2&H6 H3&H6 H4&H6
Chromosome pair

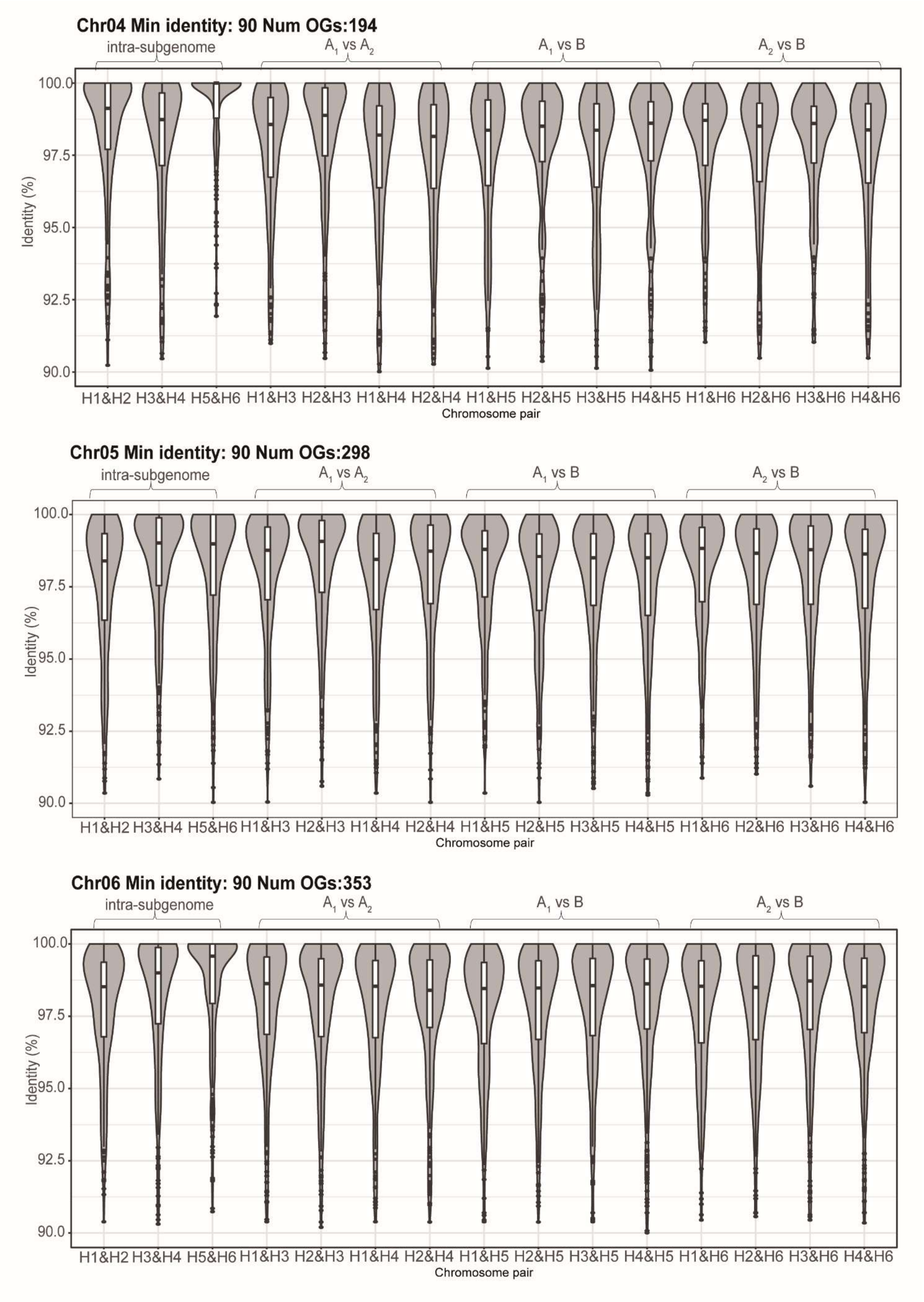
Chr04 Min identity: 90 Num OGs:194
intra-subgenome
A_1 vs A_2
A_1 vs B
A_2 vs B
Identity (%)
100.0
97.5
95.0
92.5
90.0
H1&H2 H3&H4 H5&H6 H1&H3 H2&H3 H1&H4 H2&H4 H1&H5 H2&H5 H3&H5 H4&H5 H1&H6 H2&H6 H3&H6 H4&H6
Chromosome pair
Chr05 Min identity: 90 Num OGs:298
intra-subgenome
A_1 vs A_2
A_1 vs B
A_2 vs B
Identity (%)
100.0
97.5
95.0
92.5
90.0
H1&H2 H3&H4 H5&H6 H1&H3 H2&H3 H1&H4 H2&H4 H1&H5 H2&H5 H3&H5 H4&H5 H1&H6 H2&H6 H3&H6 H4&H6
Chromosome pair
Chr06 Min identity: 90 Num OGs:353
intra-subgenome
A_1 vs A_2
A_1 vs B
A_2 vs B
Identity (%)
100.0
97.5
95.0
92.5
90.0
H1&H2 H3&H4 H5&H6 H1&H3 H2&H3 H1&H4 H2&H4 H1&H5 H2&H5 H3&H5 H4&H5 H1&H6 H2&H6 H3&H6 H4&H6
Chromosome pair

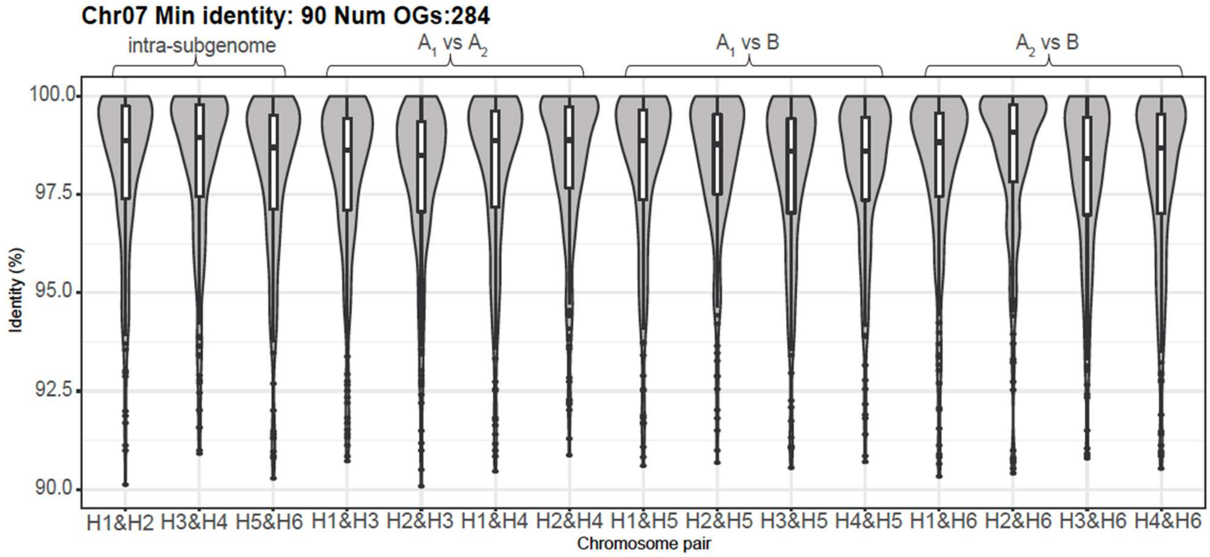
Chr07 Min identity: 90 Num OGs:284
intra-subgenome
A1 vs A2
A1 vs B
A2 vs B
Identity (%)
100.0
97.5
95.0
92.5
90.0
H1&H2 H3&H4 H5&H6 H1&H3 H2&H3 H1&H4 H2&H4 H1&H5 H2&H5 H3&H5 H4&H5 H1&H6 H2&H6 H3&H6 H4&H6
Chromosome pair

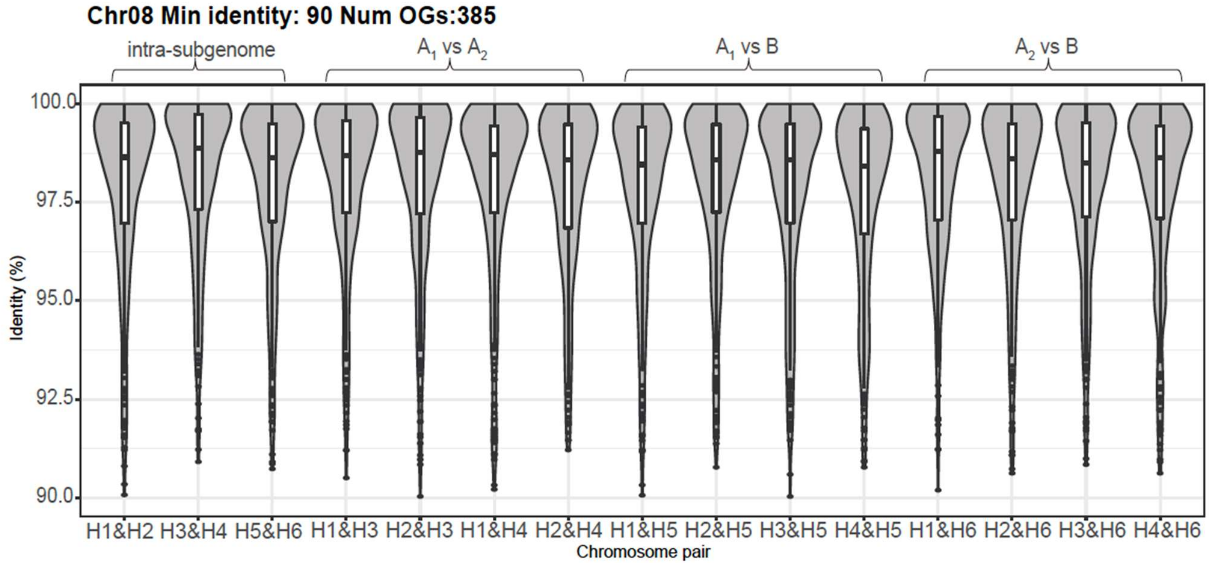
Chr08 Min identity: 90 Num OGs:385
intra-subgenome
A1 vs A2
A1 vs B
A2 vs B
Identity (%)
100.0
97.5
95.0
92.5
90.0
H1&H2 H3&H4 H5&H6 H1&H3 H2&H3 H1&H4 H2&H4 H1&H5 H2&H5 H3&H5 H4&H5 H1&H6 H2&H6 H3&H6 H4&H6
Chromosome pair

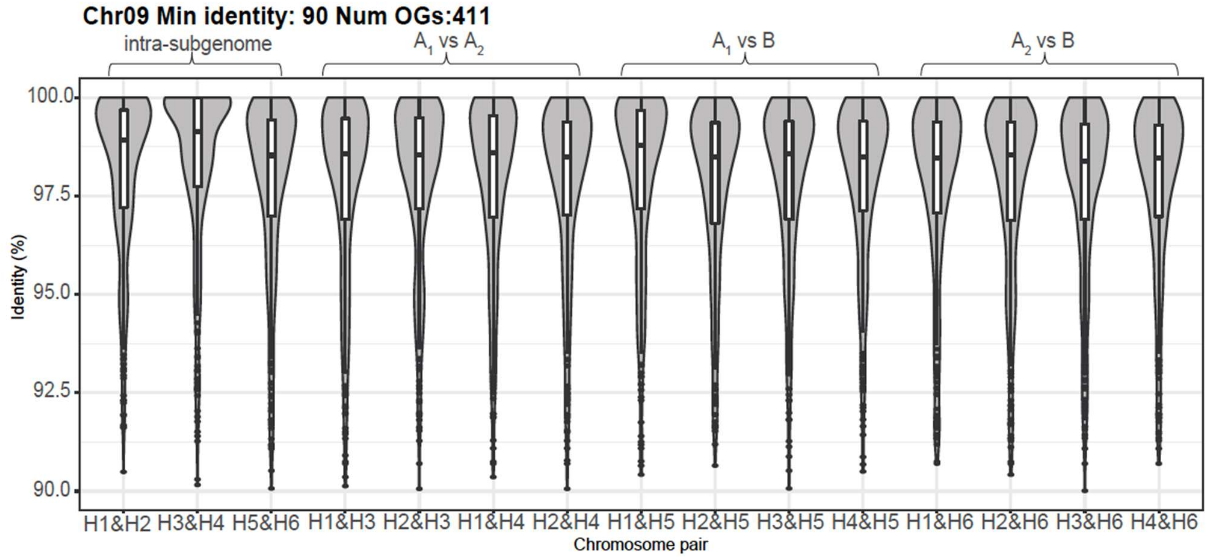
Chr09 Min identity: 90 Num OGs:411
intra-subgenome
A1 vs A2
A1 vs B
A2 vs B
Identity (%)
100.0
97.5
95.0
92.5
90.0
H1&H2 H3&H4 H5&H6 H1&H3 H2&H3 H1&H4 H2&H4 H1&H5 H2&H5 H3&H5 H4&H5 H1&H6 H2&H6 H3&H6 H4&H6
Chromosome pair

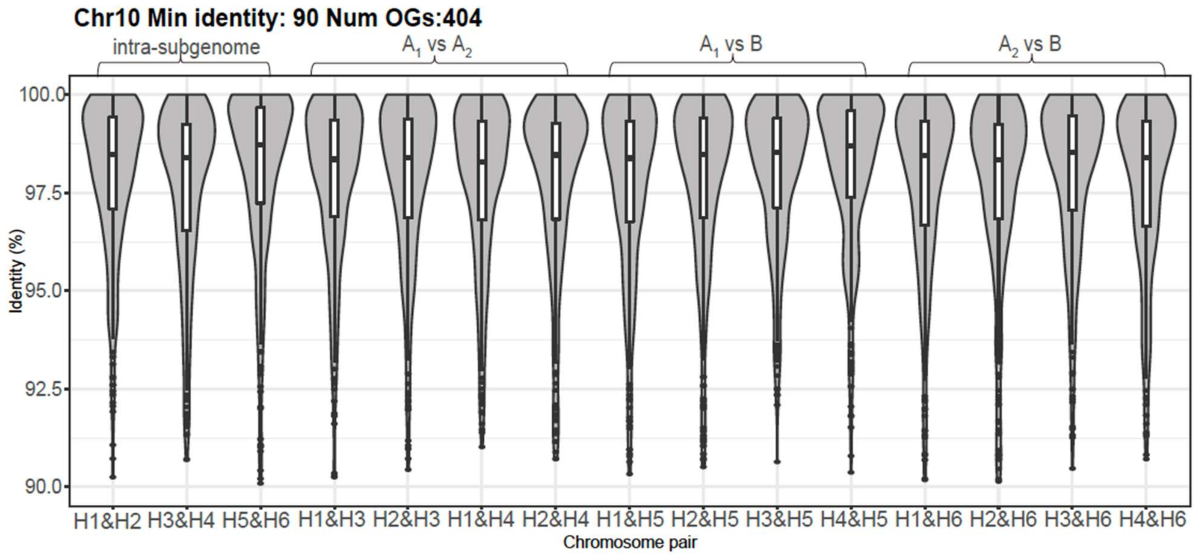
Chr10 Min identity: 90 Num OGs:404
intra-subgenome
A1 vs A2
A1 vs B
A2 vs B
Identity (%)
100.0
97.5
95.0
92.5
90.0
H1&H2 H3&H4 H5&H6 H1&H3 H2&H3 H1&H4 H2&H4 H1&H5 H2&H5 H3&H5 H4&H5 H1&H6 H2&H6 H3&H6 H4&H6
Chromosome pair

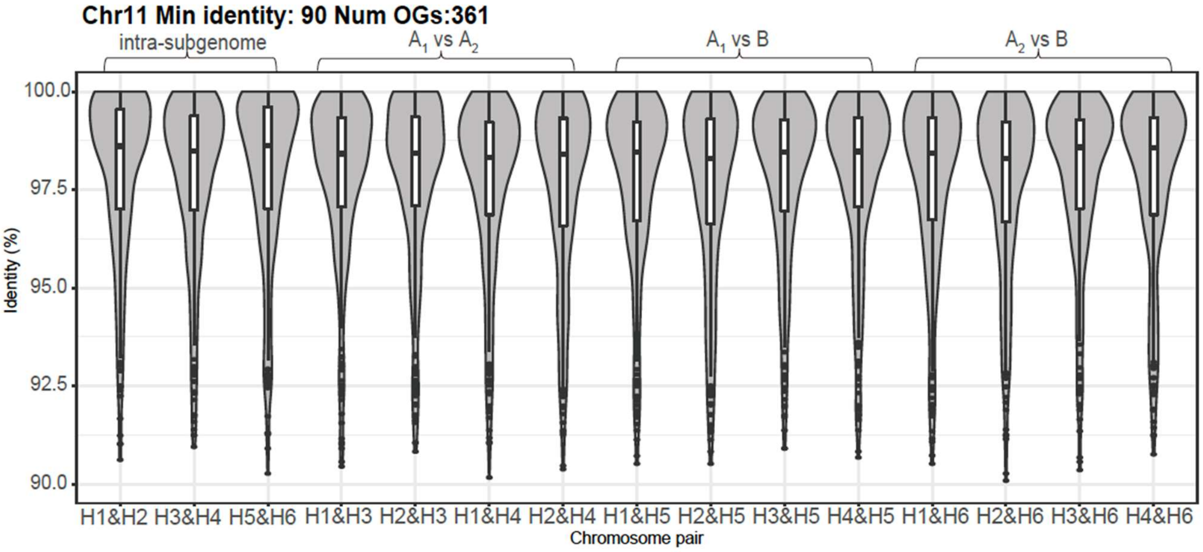
Chr11 Min identity: 90 Num OGs:361
intra-subgenome
A1 vs A2
A1 vs B
A2 vs B
Identity (%)
100.0
97.5
95.0
92.5
90.0
H1&H2 H3&H4 H5&H6 H1&H3 H2&H3 H1&H4 H2&H4 H1&H5 H2&H5 H3&H5 H4&H5 H1&H6 H2&H6 H3&H6 H4&H6
Chromosome pair

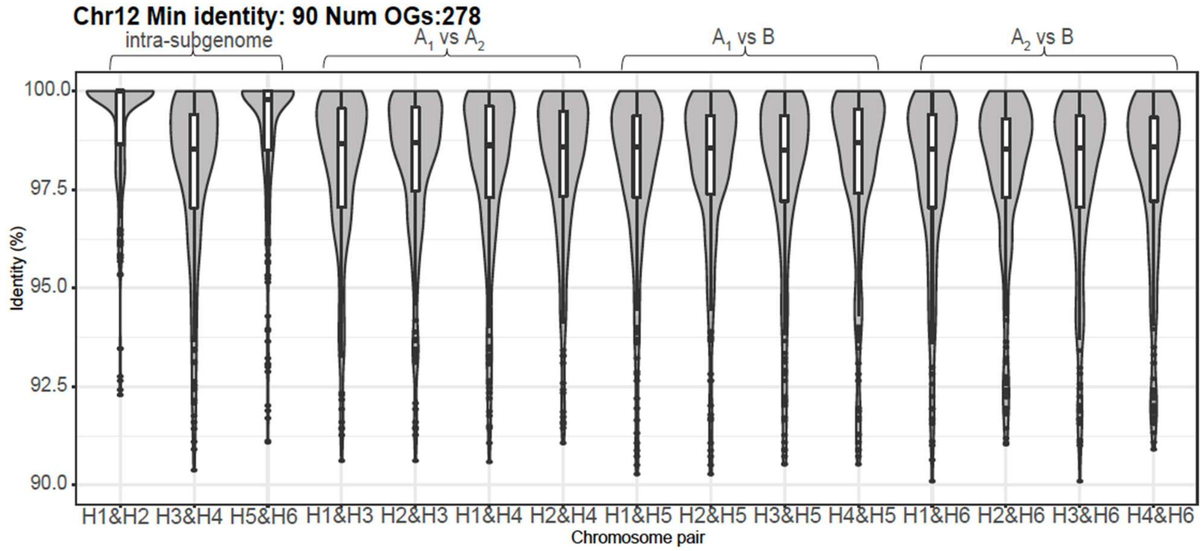
Chr12 Min identity: 90 Num OGs:278
intra-subgenome
A1 vs A2
A1 vs B
A2 vs B
Identity (%)
100.0
97.5
95.0
92.5
90.0
H1&H2 H3&H4 H5&H6 H1&H3 H2&H3 H1&H4 H2&H4 H1&H5 H2&H5 H3&H5 H4&H5 H1&H6 H2&H6 H3&H6 H4&H6
Chromosome pair

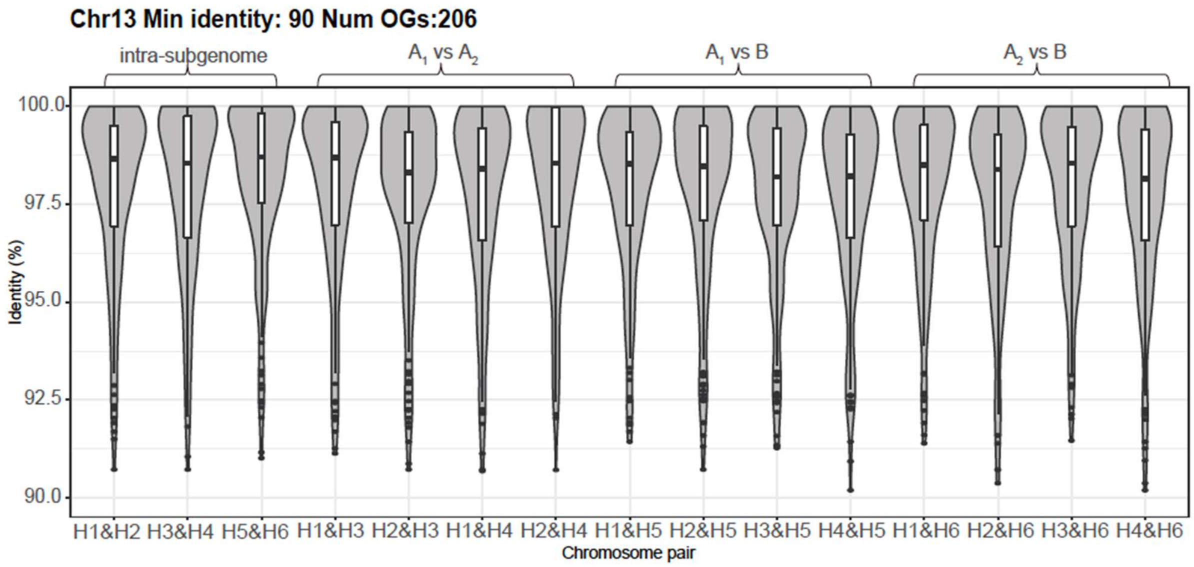
Chr13 Min identity: 90 Num OGs:206
intra-subgenome
A_1 vs A_2
A_1 vs B
A_2 vs B
Identity (%)
100.0
97.5
95.0
92.5
90.0
H1&H2 H3&H4 H5&H6 H1&H3 H2&H3 H1&H4 H2&H4 H1&H5 H2&H5 H3&H5 H4&H5 H1&H6 H2&H6 H3&H6 H4&H6
Chromosome pair

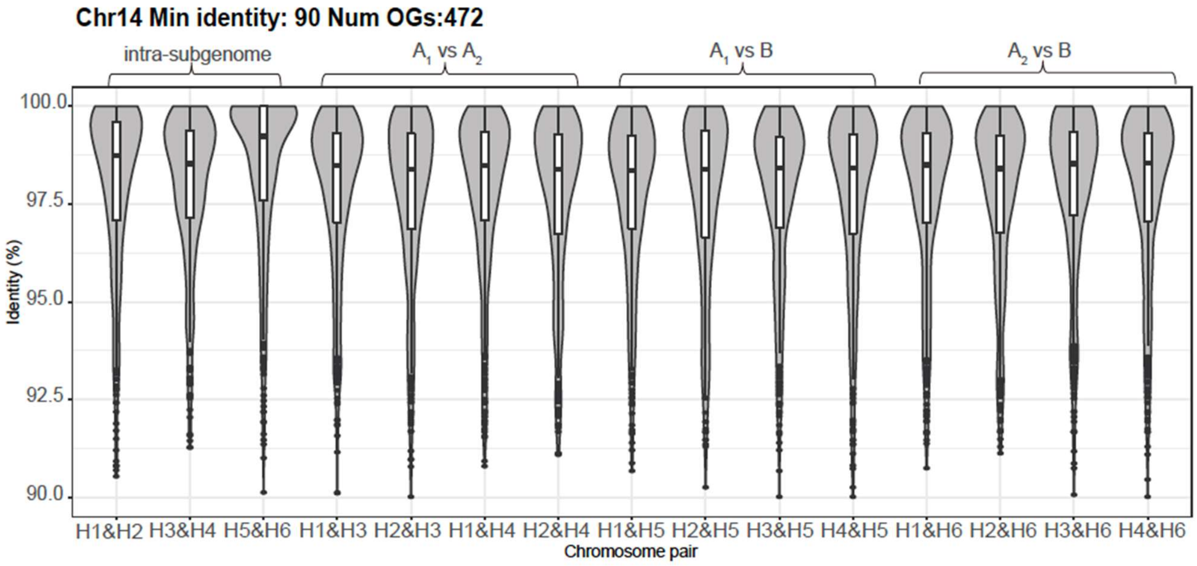
Chr14 Min identity: 90 Num OGs:472
intra-subgenome
A_1 vs A_2
A_1 vs B
A_2 vs B
Identity (%)
100.0
97.5
95.0
92.5
90.0
H1&H2 H3&H4 H5&H6 H1&H3 H2&H3 H1&H4 H2&H4 H1&H5 H2&H5 H3&H5 H4&H5 H1&H6 H2&H6 H3&H6 H4&H6
Chromosome pair

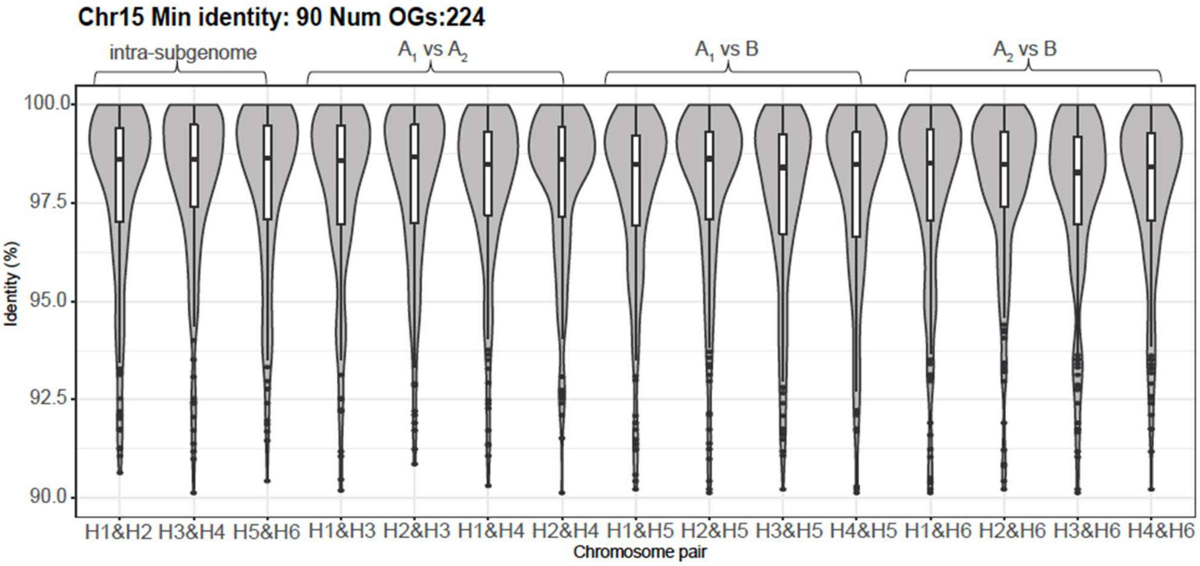
Chr15 Min identity: 90 Num OGs:224
intra-subgenome
A_1 vs A_2
A_1 vs B
A_2 vs B
Identity (%)
100.0
97.5
95.0
92.5
90.0
H1&H2 H3&H4 H5&H6 H1&H3 H2&H3 H1&H4 H2&H4 H1&H5 H2&H5 H3&H5 H4&H5 H1&H6 H2&H6 H3&H6 H4&H6
Chromosome pair

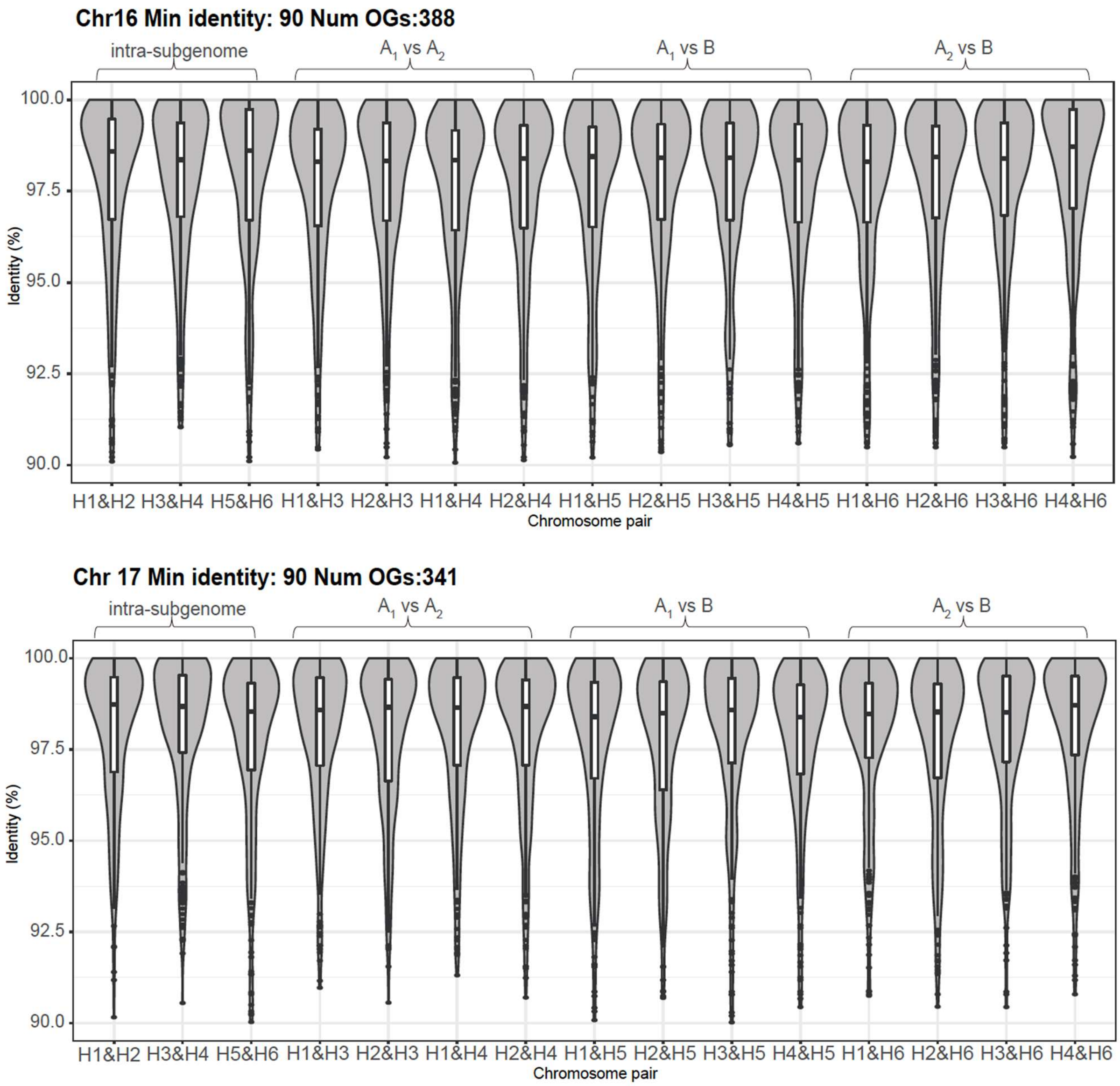

Supplemental Figure 8 Violin and box plot of alignment identities of single-copy genes (OGs) between homologous chromosomes from the same sub-genome and different sub-genomes for hexaploid *H. tuberosus*. For each homologous group, the single-copy genes among 6 homologous chromosomes were identified using OrthoFinder. For each pair of homologous chromosomes, the multiple sequence alignments of single-copy genes with identities higher than 90% were plotted. Solid circles refer to outliers and horizontal bold lines in box plots indicate medians.

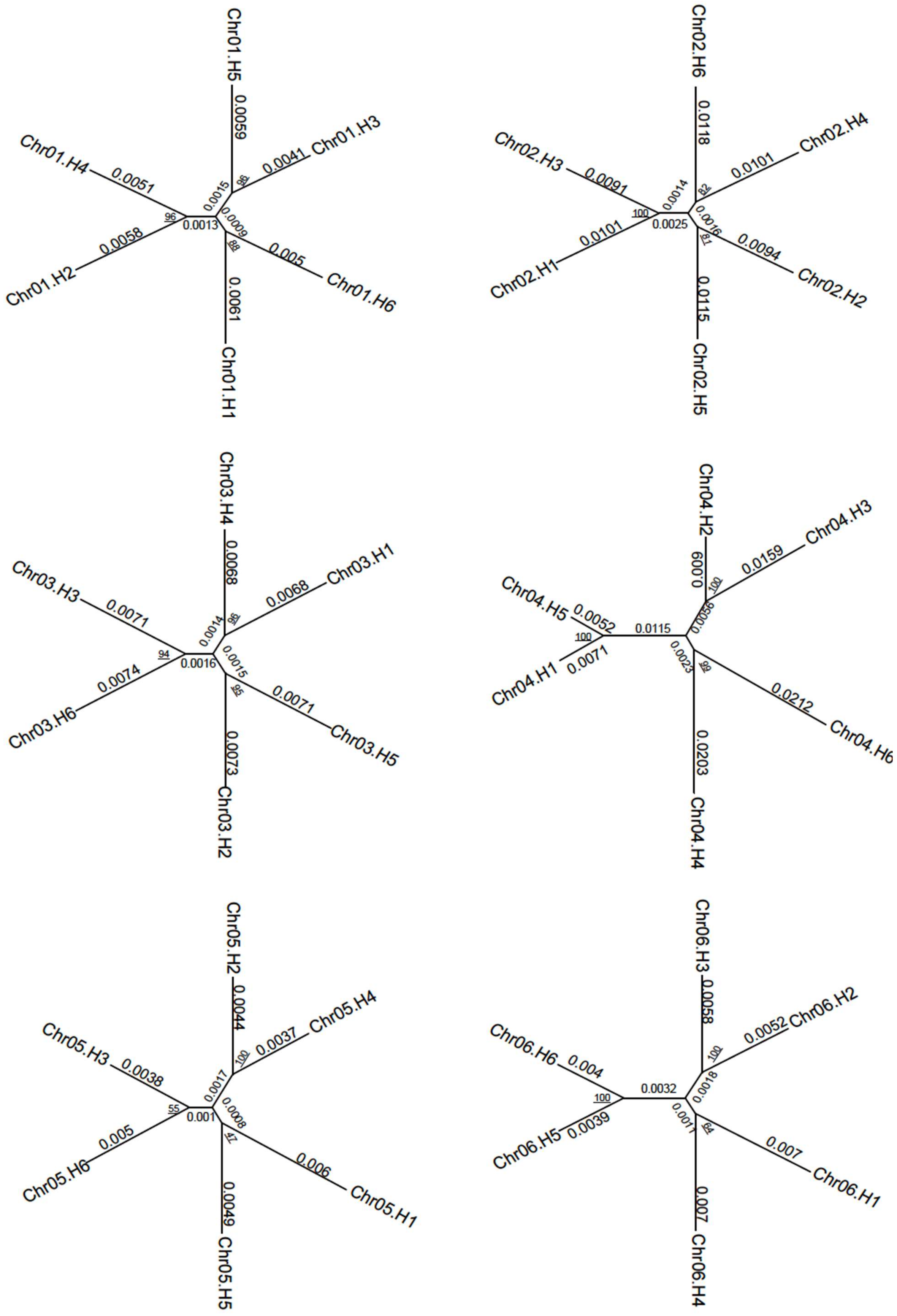
Chr01.H5
Chr01.H3
Chr01.H4
Chr01.H2
Chr01.H6
Chr01.H1
0.0059
0.0041
0.0051
0.0015
96
0.0013
0.0009
0.0058
0.005
0.0061
Chr02.H6
Chr02.H4
Chr02.H3
Chr02.H1
Chr02.H2
Chr02.H5
0.0118
0.0101
0.0091
0.0014
82
100
0.0025
0.0016
81
0.0101
0.0094
0.0115
Chr03.H4
Chr03.H1
Chr03.H3
Chr03.H6
Chr03.H5
Chr03.H2
0.0068
0.0068
0.0071
0.0014
94
0.0016
0.0015
0.0074
0.0071
0.0073
Chr04.H2
Chr04.H3
Chr04.H5
Chr04.H1
Chr04.H6
Chr04.H4
0.009
0.0159
100
0.0052
0.0115
0.0056
100
0.0071
0.0023
0.0212
0.0203
Chr05.H2
Chr05.H4
Chr05.H3
Chr05.H6
Chr05.H1
Chr05.H5
0.0044
0.0037
100
0.0038
0.0017
55
0.001
0.0008
47
0.005
0.006
0.0049
Chr06.H3
Chr06.H2
Chr06.H6
Chr06.H5
Chr06.H1
Chr06.H4
0.0058
0.0052
100
0.004
0.0032
0.0018
100
0.0039
0.0011
0.007
0.007

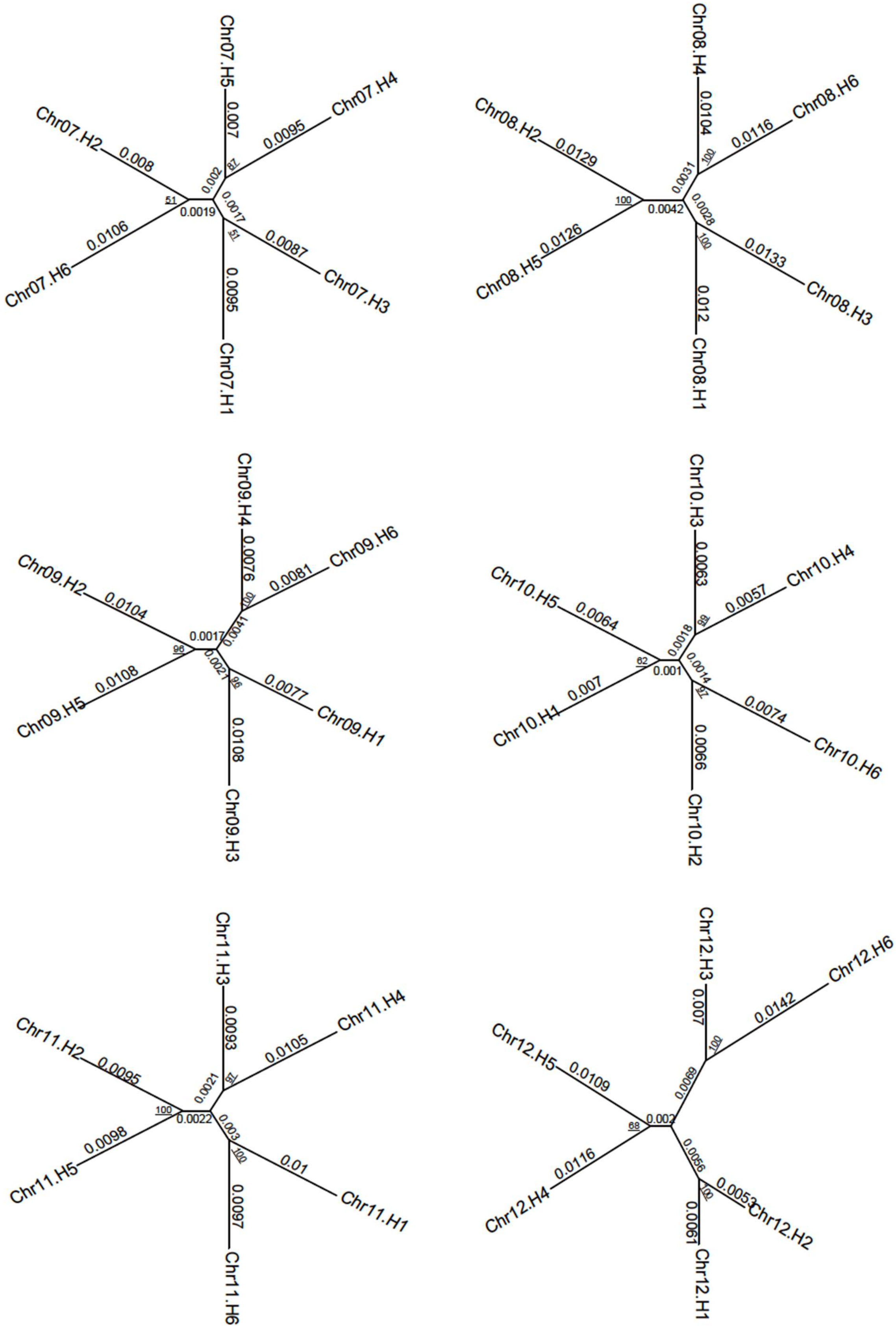
Chr07.H5
Chr07.H4
Chr07.H2
Chr07.H6
Chr07.H3
Chr07.H1
0.007
0.0095
0.008
0.002
87
51
0.0019
0.0017
0.0106
51
0.0087
0.0095
Chr08.H4
Chr08.H6
Chr08.H2
Chr08.H5
Chr08.H3
Chr08.H1
0.0104
0.0116
0.0129
0.0031
100
100
0.0042
0.0028
0.0126
100
0.0133
0.012
Chr09.H4
Chr09.H6
Chr09.H2
Chr09.H5
Chr09.H1
Chr09.H3
0.0076
0.0081
0.0104
0.0017
0.0041
96
0.0021
0.0108
0.0077
0.0108
Chr10.H3
Chr10.H4
Chr10.H5
Chr10.H1
Chr10.H6
Chr10.H2
0.0063
0.0057
0.0064
0.0018
62
0.001
0.0014
0.007
0.0074
0.0066
Chr11.H3
Chr11.H4
Chr11.H2
Chr11.H5
Chr11.H1
Chr11.H6
0.0093
0.0105
0.0095
0.0021
87
100
0.0022
0.003
0.0098
100
0.01
0.0097
Chr12.H3
Chr12.H6
Chr12.H5
Chr12.H4
Chr12.H2
Chr12.H1
0.007
0.0142
100
0.0109
0.0069
68
0.002
0.0116
0.0056
100
0.0053
0.0061

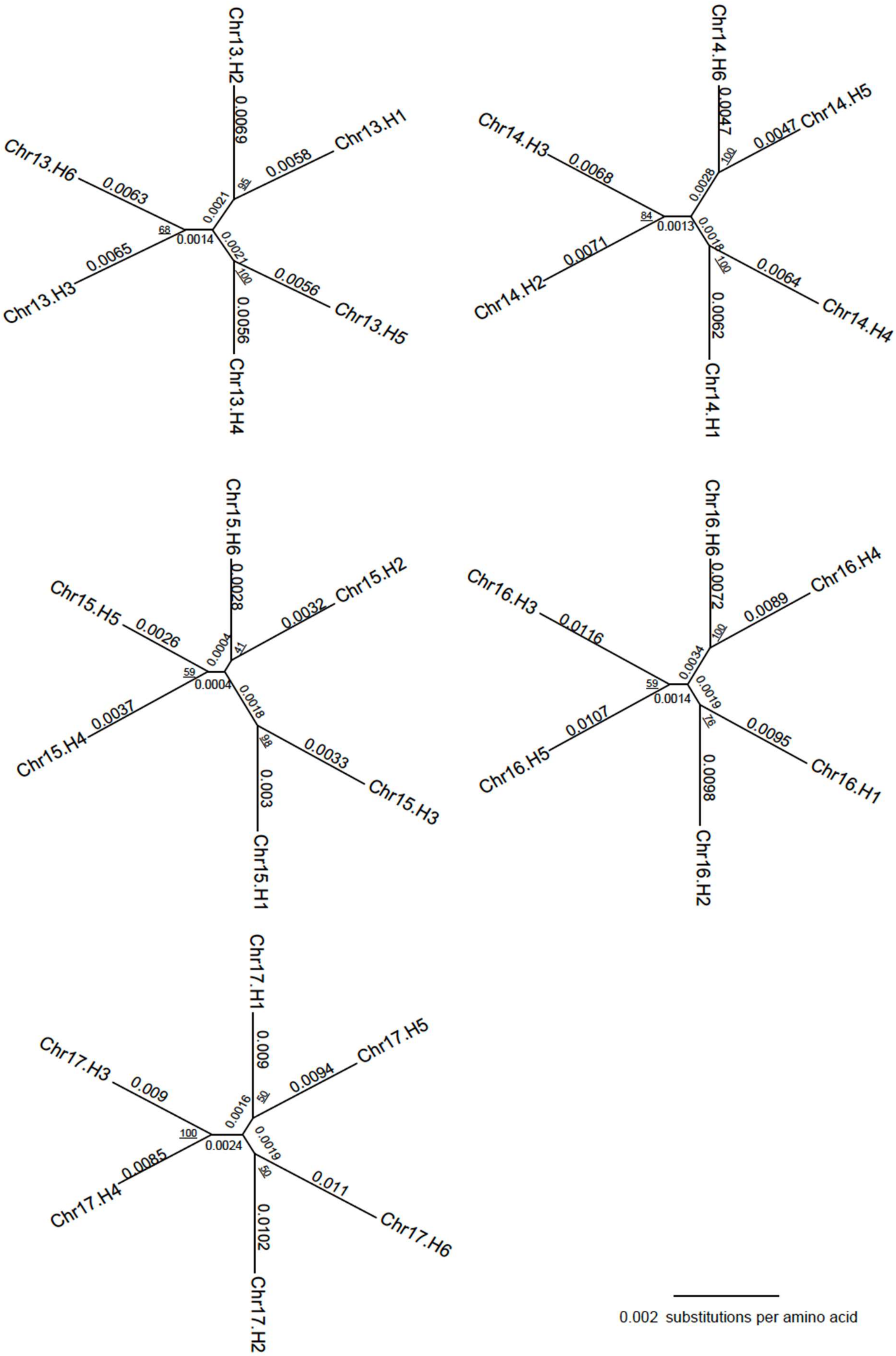
Chr13.H2
0.0069
Chr13.H1
0.0058
95
0.0021
Chr13.H6
0.0063
68
0.0014
0.0021
100
0.0056
Chr13.H5
Chr13.H3
0.0065
0.0056
Chr13.H4
Chr14.H6
0.0047
Chr14.H5
0.0047
100
0.0028
Chr14.H3
0.0068
84
0.0013
0.0018
100
0.0064
Chr14.H4
Chr14.H2
0.0071
0.0062
Chr14.H1
Chr15.H6
0.0028
Chr15.H2
0.0032
41
0.0004
Chr15.H5
0.0026
59
0.0004
0.0018
98
0.0033
Chr15.H3
Chr15.H4
0.0037
0.003
Chr15.H1
Chr16.H6
0.0072
Chr16.H4
0.0089
100
0.0034
Chr16.H3
0.0116
59
0.0014
0.0019
76
0.0095
Chr16.H1
Chr16.H5
0.0107
0.0098
Chr16.H2
Chr17.H1
0.009
Chr17.H5
0.0094
50
0.0016
Chr17.H3
0.009
100
0.0024
0.0019
50
0.011
Chr17.H6
Chr17.H4
0.0085
0.0102
Chr17.H2
0.002 substitutions per amino acid

Supplemental Figure 9 Unrooted phylogeny trees of 17 groups of homologous chromosomes for hexaploid *H. tuberosus*. For each homologous group, the phylogeny tree was constructed using RAxMl-NG (ML method, LG+G8+F model) on the gap-trimmed concatenated multiple sequence alignment of single-copy genes for the 6 homologous chromosomes (numbers of used genes given in Table 2 in the main text). Branch lengths indicate number of substitutions per amino acid, and underlined integers refer to bootstrap supports of the corresponding nodes.

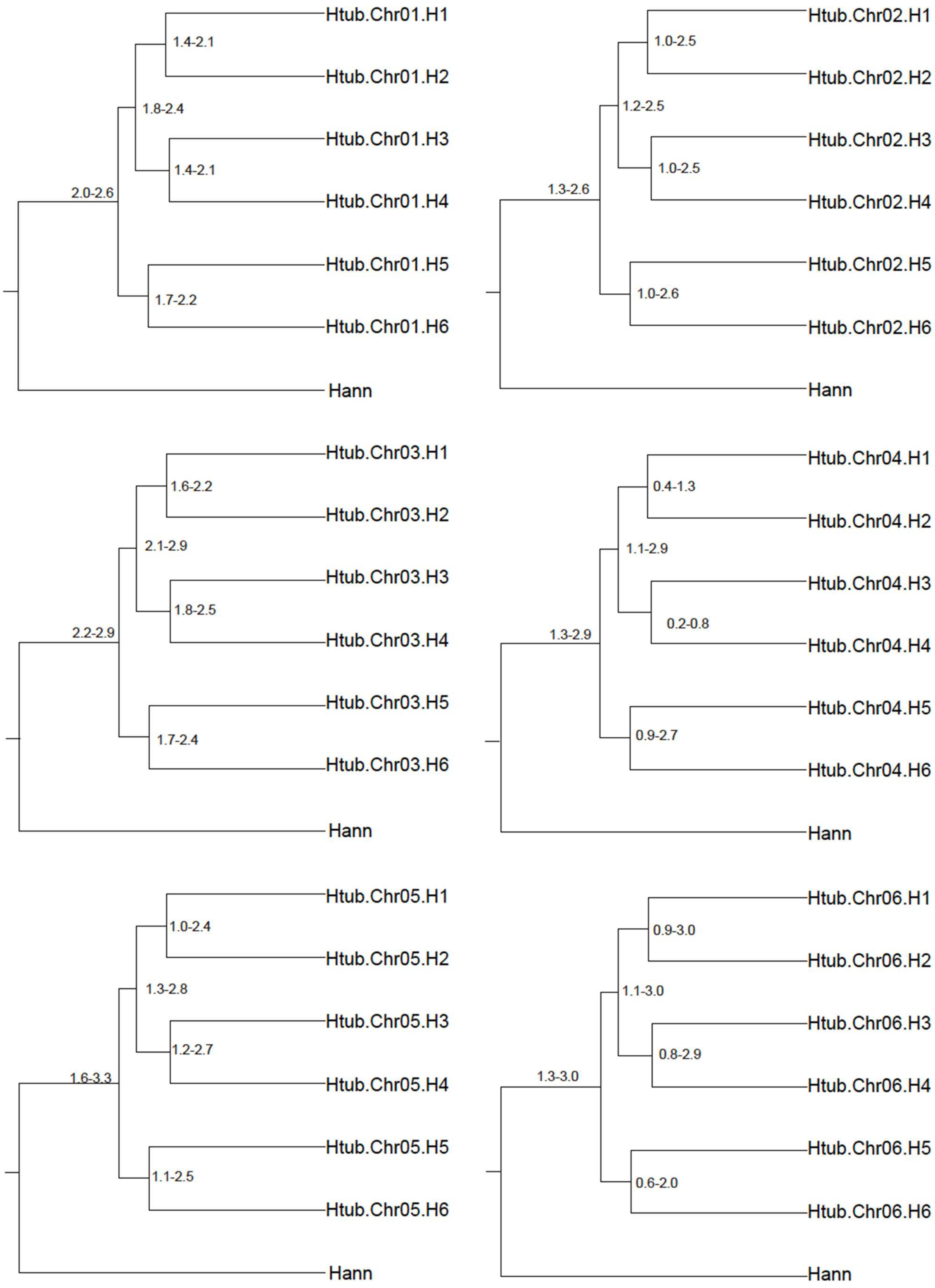

Htub.Chr01.H1
1.4-2.1
Htub.Chr01.H2
1.8-2.4
Htub.Chr01.H3
1.4-2.1
2.0-2.6
Htub.Chr01.H4
Htub.Chr01.H5
1.7-2.2
Htub.Chr01.H6
Hann
Htub.Chr02.H1
1.0-2.5
Htub.Chr02.H2
1.2-2.5
Htub.Chr02.H3
1.0-2.5
1.3-2.6
Htub.Chr02.H4
Htub.Chr02.H5
1.0-2.6
Htub.Chr02.H6
Hann
Htub.Chr03.H1
1.6-2.2
Htub.Chr03.H2
2.1-2.9
Htub.Chr03.H3
1.8-2.5
2.2-2.9
Htub.Chr03.H4
Htub.Chr03.H5
1.7-2.4
Htub.Chr03.H6
Hann
Htub.Chr04.H1
0.4-1.3
Htub.Chr04.H2
1.1-2.9
Htub.Chr04.H3
0.2-0.8
1.3-2.9
Htub.Chr04.H4
Htub.Chr04.H5
0.9-2.7
Htub.Chr04.H6
Hann
Htub.Chr05.H1
1.0-2.4
Htub.Chr05.H2
1.3-2.8
Htub.Chr05.H3
1.2-2.7
1.6-3.3
Htub.Chr05.H4
Htub.Chr05.H5
1.1-2.5
Htub.Chr05.H6
Hann
Htub.Chr06.H1
0.9-3.0
Htub.Chr06.H2
1.1-3.0
Htub.Chr06.H3
0.8-2.9
1.3-3.0
Htub.Chr06.H4
Htub.Chr06.H5
0.6-2.0
Htub.Chr06.H6
Hann

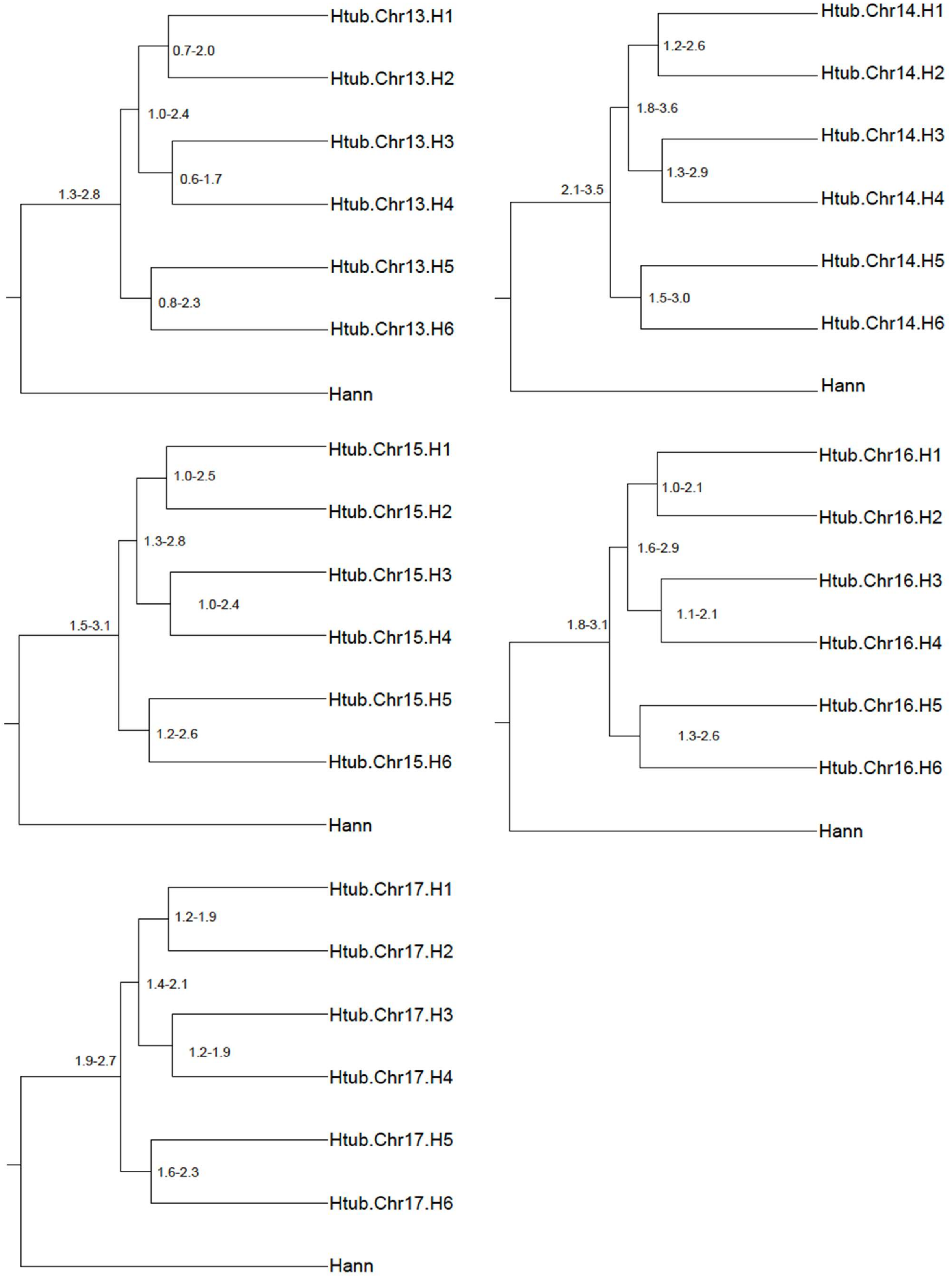

Htub.Chr13.H1
0.7-2.0
Htub.Chr13.H2
1.0-2.4
Htub.Chr13.H3
0.6-1.7
1.3-2.8
Htub.Chr13.H4
Htub.Chr13.H5
0.8-2.3
Htub.Chr13.H6
Hann
Htub.Chr14.H1
1.2-2.6
Htub.Chr14.H2
1.8-3.6
Htub.Chr14.H3
1.3-2.9
2.1-3.5
Htub.Chr14.H4
Htub.Chr14.H5
1.5-3.0
Htub.Chr14.H6
Hann
Htub.Chr15.H1
1.0-2.5
Htub.Chr15.H2
1.3-2.8
Htub.Chr15.H3
1.0-2.4
1.5-3.1
Htub.Chr15.H4
Htub.Chr15.H5
1.2-2.6
Htub.Chr15.H6
Hann
Htub.Chr16.H1
1.0-2.1
Htub.Chr16.H2
1.6-2.9
Htub.Chr16.H3
1.1-2.1
1.8-3.1
Htub.Chr16.H4
Htub.Chr16.H5
1.3-2.6
Htub.Chr16.H6
Hann
Htub.Chr17.H1
1.2-1.9
Htub.Chr17.H2
1.4-2.1
Htub.Chr17.H3
1.2-1.9
1.9-2.7
Htub.Chr17.H4
Htub.Chr17.H5
1.6-2.3
Htub.Chr17.H6
Hann

Supplemental Figure 10 Estimated divergence times of 17 groups of homologous chromosomes for hexaploid *H. tuberosus (Htub)* and *H. annuus (Hann)*. For each homologous group, the divergence time tree was estimated using MEGA RelTime-ML with the phylogeny tree constructed on the gap-trimmed concatenated multiple sequence alignment of single-copy genes among the 6 homologous chromosomes and *Hann*, with one calibration of the divergence of *Hann* and *Mikania micrantha* 16-27 million years ago (MYA). For simplicity, the outgroup branches of *Mikania micrantha* were not shown.

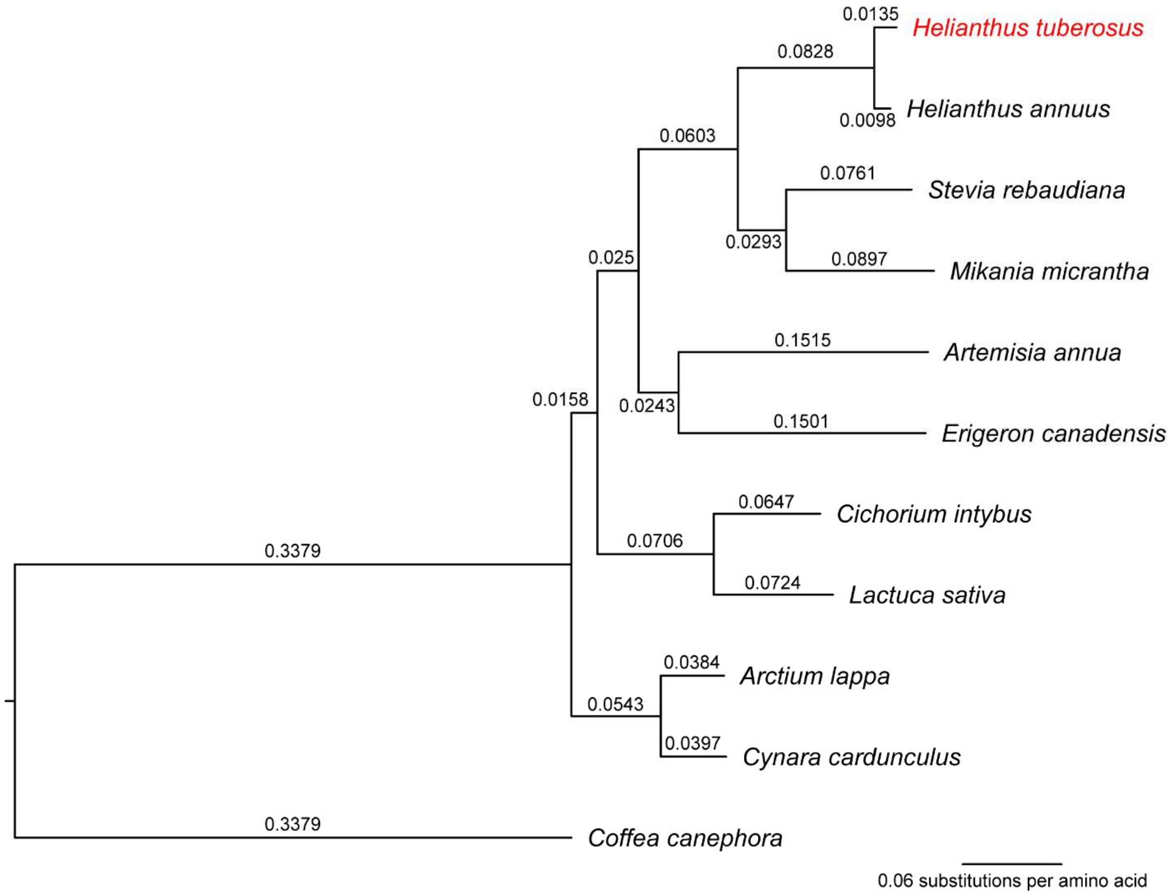

Supplemental Figure 11 Phylogenetic tree of 10 Asteraceae species and one outgroup *Coffea canephora*. The tree was constructed using RAxML-NG (ML method, LG+G8+F model) based on the concatenated multiple protein sequence alignment of 1,109 conserved ortholog groups (OGs) among these 11 species, and *H. tuberosus* is highlighted in red. Tree branch lengths indicate number of substitutions per amino acid.

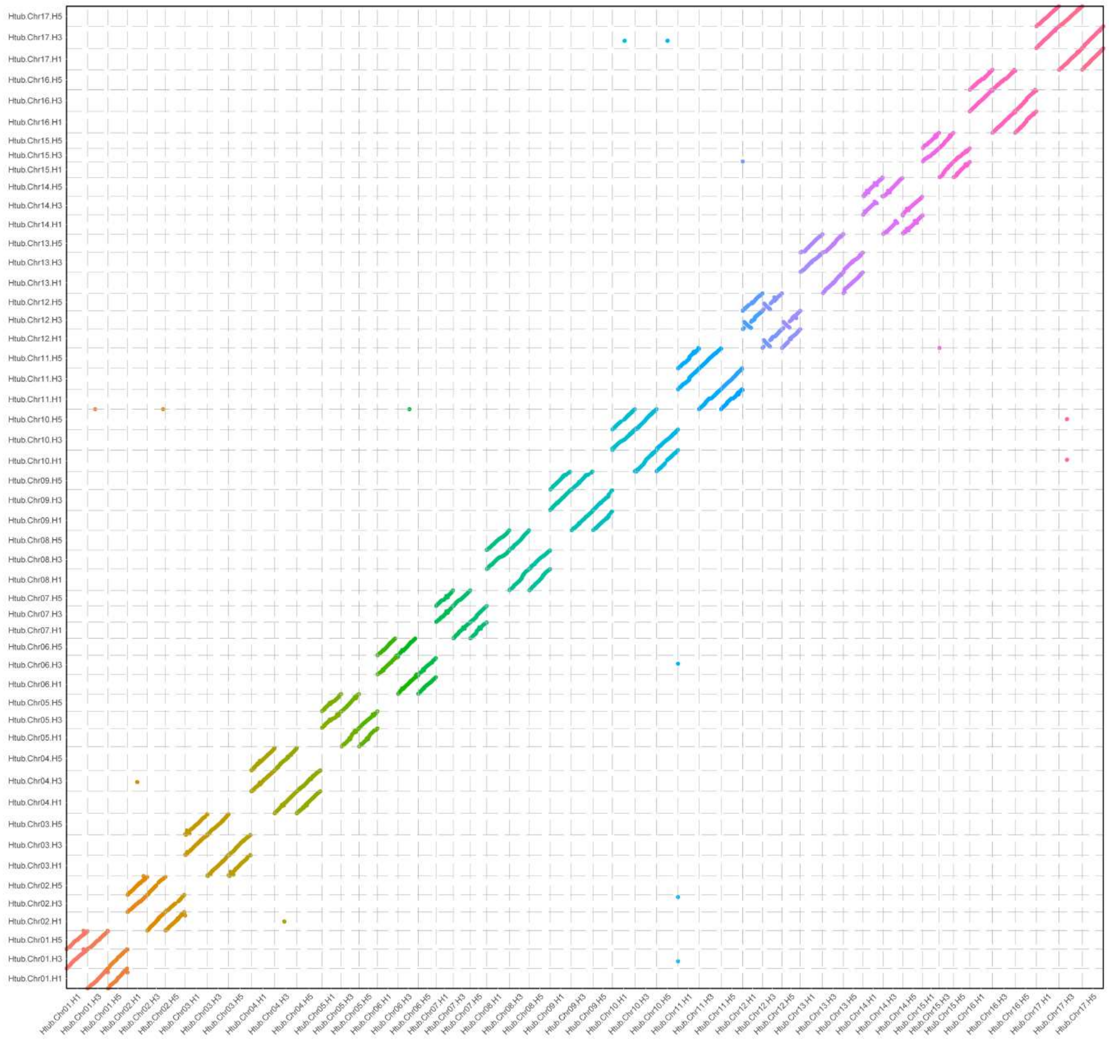

Supplemental Figure 12 Intra-species synteny dot plot of 51 chromosomes of reference genome for *H. tuberosus.* The synteny analysis was done using MCScanX with the all-vs-all alignment of protein-coding genes anchored to chromosomes as input. Each dot represents a gene pair of a syntenic block with more than 10 gene pairs and average synonymous mutation rate (Ks) lower than 0.2.

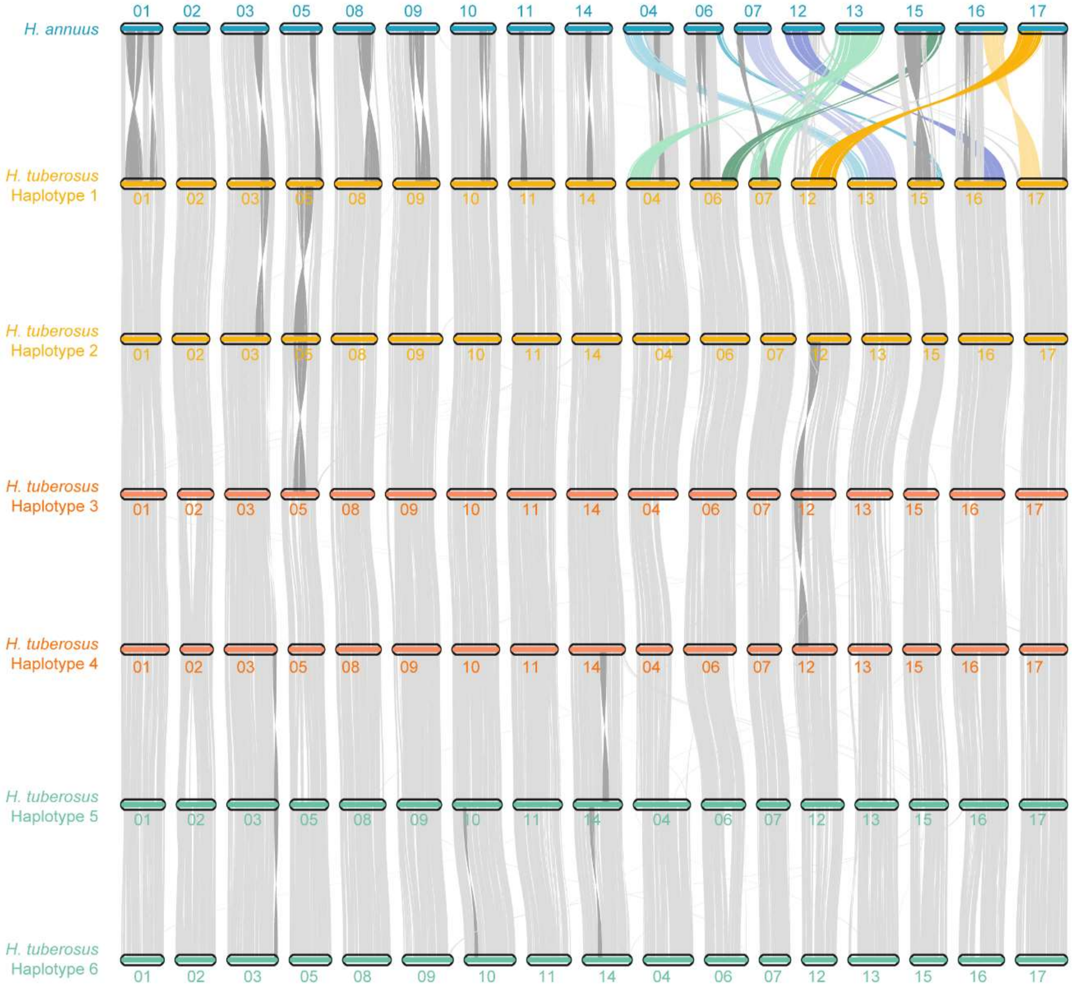

Supplemental Figure 13 Synteny plots showing the syntenic relationships among 6 haplotype chromosome sets of *H. tuberosus* and 17 chromosomes of *H. annuus*. The synteny analysis was done using MCScanX and JCVI with the all-vs-all alignment of protein-coding genes anchored to chromosomes as input. Each line represents a syntenic block with more than 10 gene pairs. The chromosome inversion and translocation blocks between chromosomes are marked in dark grey and different colors, respectively.

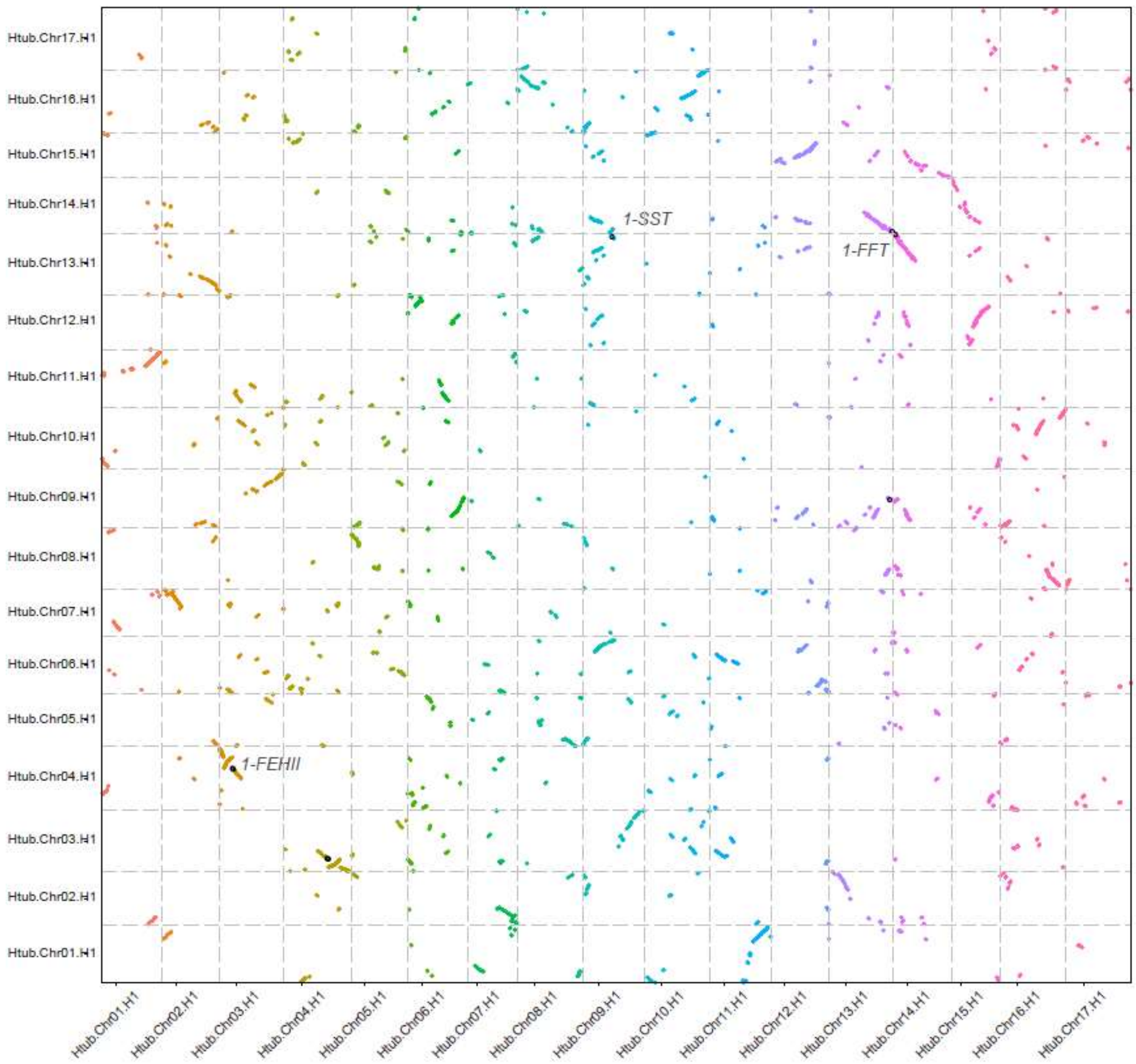

Supplemental Figure 14 Synteny dot plot showing the WGD2 on a haploid of reference genome for *H. tuberosus*. Each colored dot represents a WGD2-derived syntenic gene pair between two different chromosomes, which are the same on both sides of the diagonal. The black coil position represents the location of the genes *1-SST, 1-FFT, 1-FEHII*.

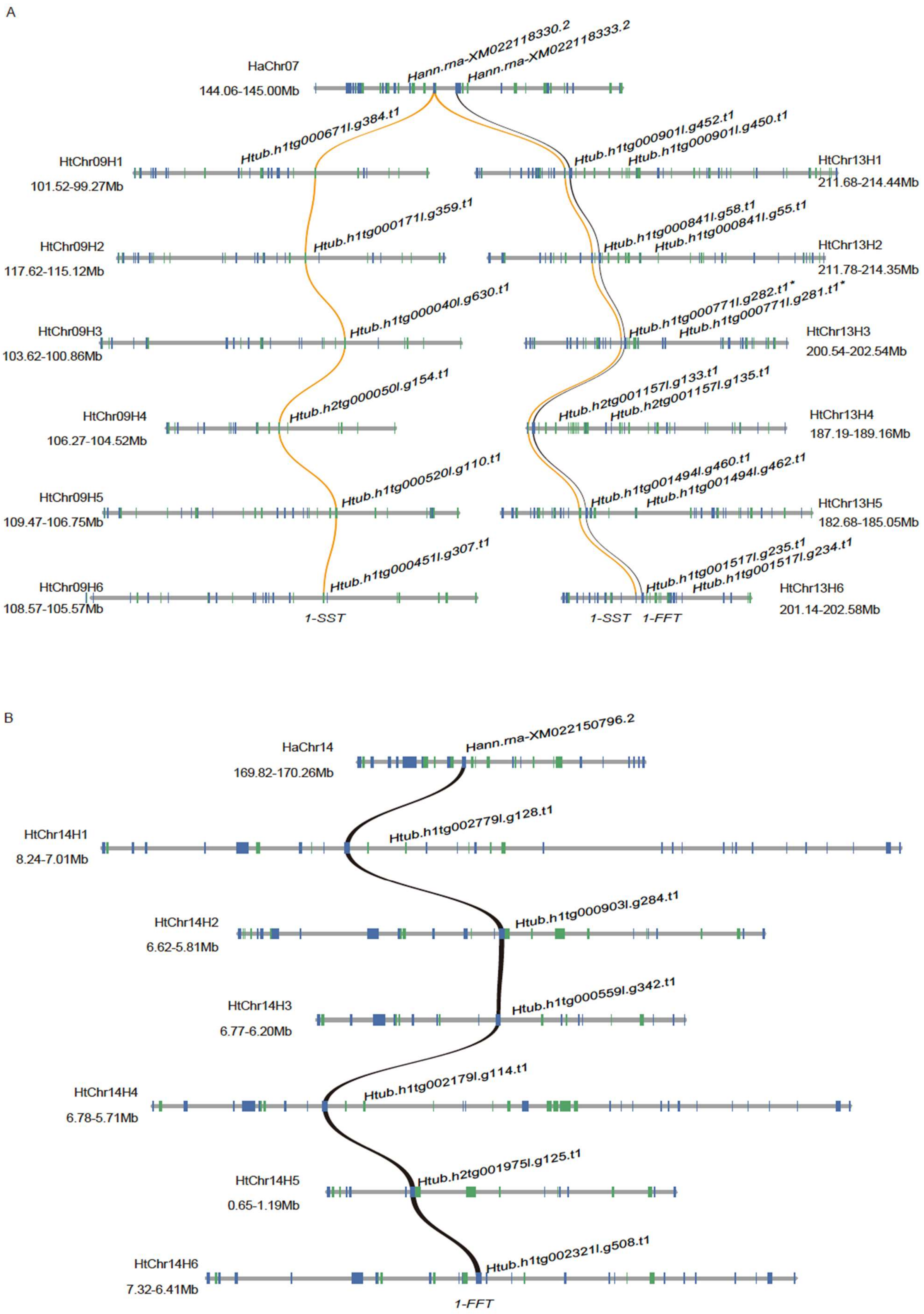
A
HaChr07
144.06-145.00Mb
Hann.rna-XM022118330.2
Hann.rna-XM022118333.2
HtChr09H1
101.52-99.27Mb
Htub.h1tg000671l.g384.t1
Htub.h1tg000901l.g452.t1
Htub.h1tg000901l.g450.t1
HtChr13H1
211.68-214.44Mb
HtChr09H2
117.62-115.12Mb
Htub.h1tg000171l.g359.t1
Htub.h1tg000841l.g58.t1
Htub.h1tg000841l.g55.t1
HtChr13H2
211.78-214.35Mb
HtChr09H3
103.62-100.86Mb
Htub.h1tg000040l.g630.t1
Htub.h1tg000771l.g282.t1*
Htub.h1tg000771l.g281.t1*
HtChr13H3
200.54-202.54Mb
HtChr09H4
106.27-104.52Mb
Htub.h2tg000050l.g154.t1
Htub.h2tg001157l.g133.t1
Htub.h2tg001157l.g135.t1
HtChr13H4
187.19-189.16Mb
HtChr09H5
109.47-106.75Mb
Htub.h1tg000520l.g110.t1
Htub.h1tg001494l.g460.t1
Htub.h1tg001494l.g462.t1
HtChr13H5
182.68-185.05Mb
HtChr09H6
108.57-105.57Mb
Htub.h1tg000451l.g307.t1
Htub.h1tg001517l.g235.t1
Htub.h1tg001517l.g234.t1
HtChr13H6
201.14-202.58Mb
1-SST
1-SST
1-FFT
B
HaChr14
169.82-170.26Mb
Hann.rna-XM022150796.2
HtChr14H1
8.24-7.01Mb
Htub.h1tg002779l.g128.t1
HtChr14H2
6.62-5.81Mb
Htub.h1tg000903l.g284.t1
HtChr14H3
6.77-6.20Mb
Htub.h1tg000559l.g342.t1
HtChr14H4
6.78-5.71Mb
Htub.h1tg002179l.g114.t1
HtChr14H5
0.65-1.19Mb
Htub.h2tg001975l.g125.t1
HtChr14H6
7.32-6.41Mb
Htub.h1tg002321l.g508.t1
1-FFT

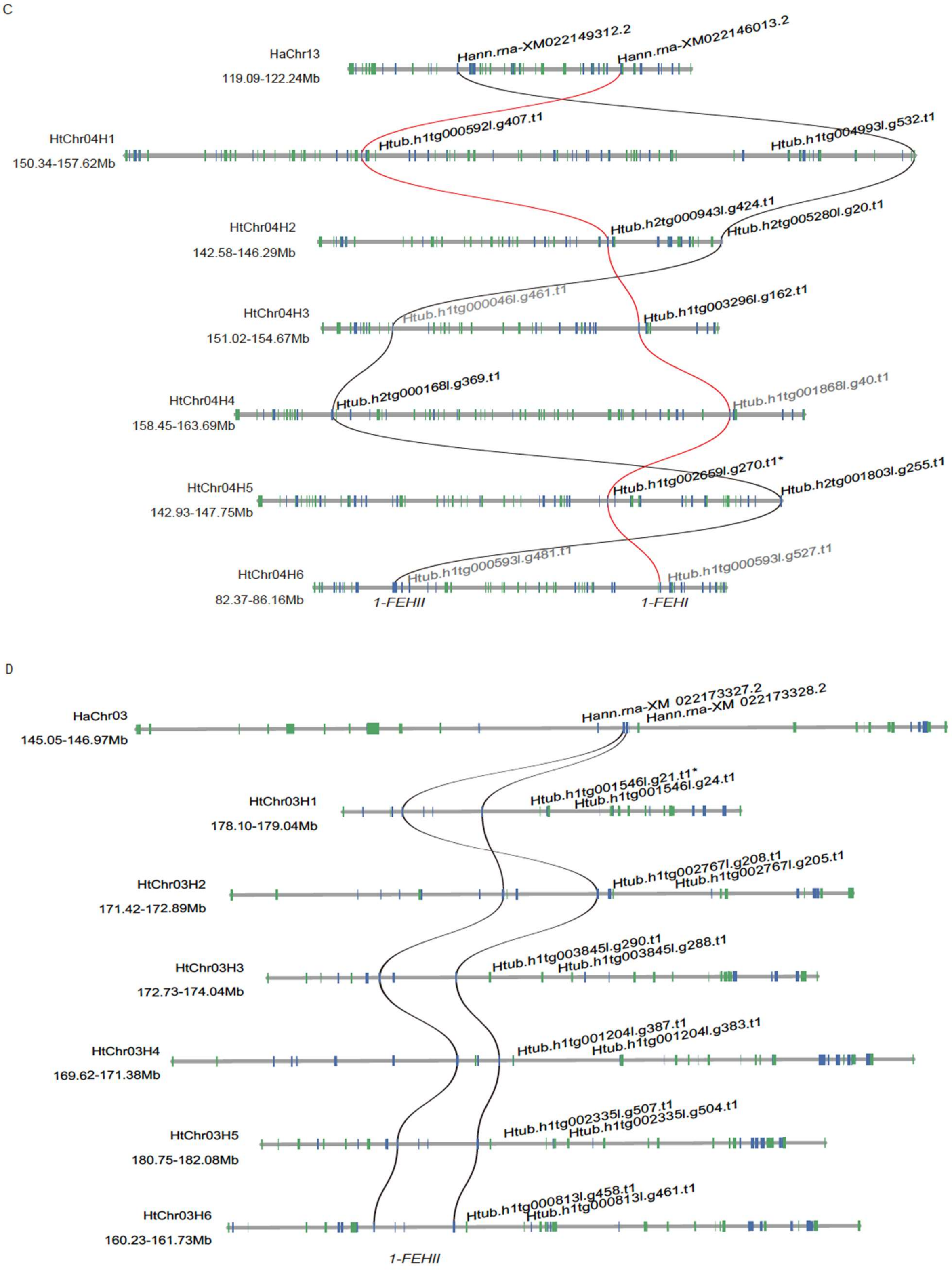

Supplemental Figure 15 Location of inulin metabolism genes on chromosomes of *H. tuberosus* (*Htub*) and *H. annuus* (*Hann*). **A** Location of *1-SST* and *1-FFT* genes. **B** Location of *1-FFT* genes. **C** Location of *1-FEHI* and *1-*

FEHII genes. **D** Location of *1-FEHII* genes. Gene names in black are true genes with complete structure, and asterisks indicate previously-cloned genes, while the gene names in gray are pseudogenes with internal stop codons, frameshifts or truncated exons.

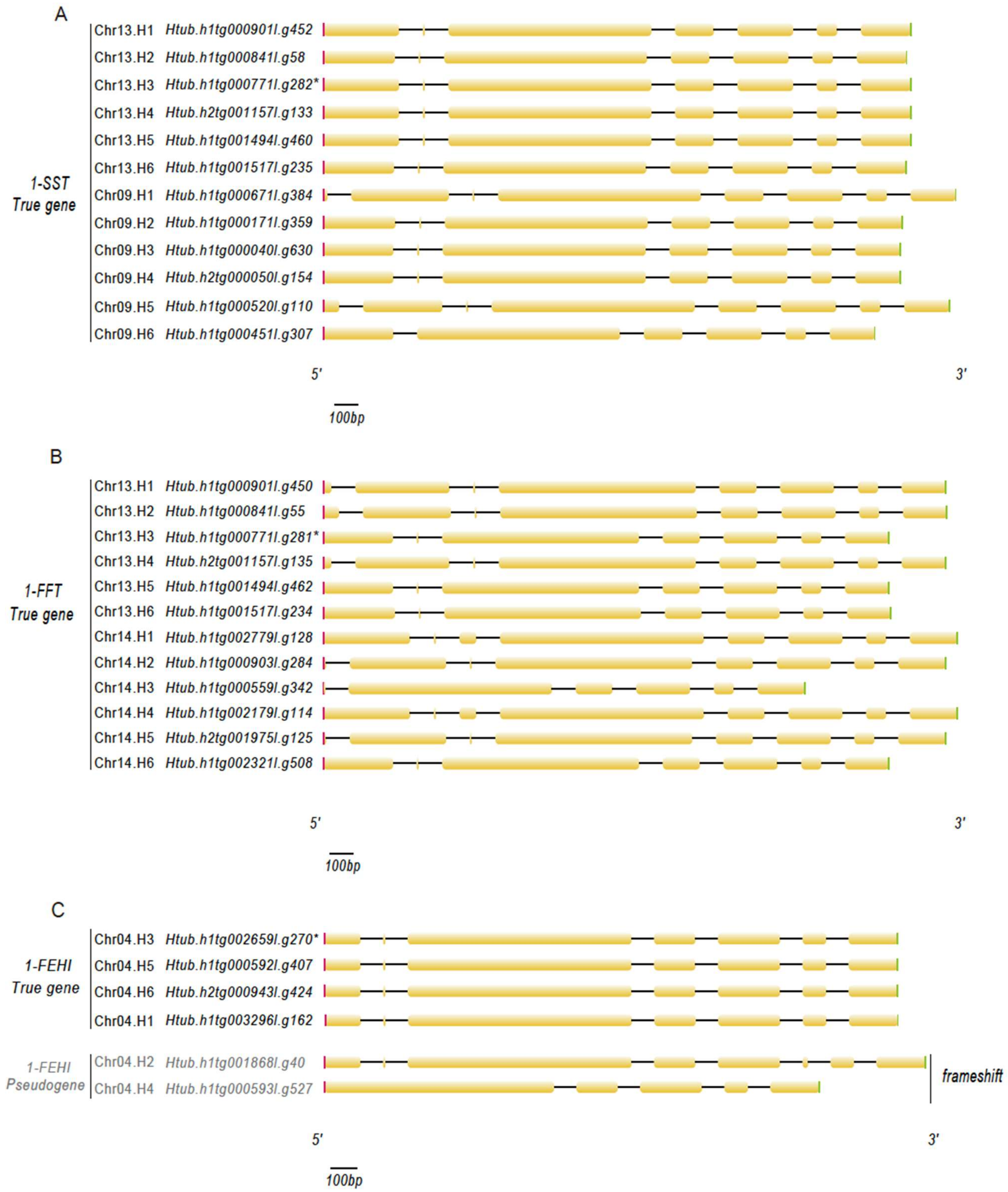
A
1-SST
True gene
Chr13.H1 Htub.h1tg000901l.g452
Chr13.H2 Htub.h1tg000841l.g58
Chr13.H3 Htub.h1tg000771l.g282*
Chr13.H4 Htub.h2tg001157l.g133
Chr13.H5 Htub.h1tg001494l.g460
Chr13.H6 Htub.h1tg001517l.g235
Chr09.H1 Htub.h1tg000671l.g384
Chr09.H2 Htub.h1tg000171l.g359
Chr09.H3 Htub.h1tg000040l.g630
Chr09.H4 Htub.h2tg000050l.g154
Chr09.H5 Htub.h1tg000520l.g110
Chr09.H6 Htub.h1tg000451l.g307
5′
3′
100bp
B
1-FFT
True gene
Chr13.H1 Htub.h1tg000901l.g450
Chr13.H2 Htub.h1tg000841l.g55
Chr13.H3 Htub.h1tg000771l.g281*
Chr13.H4 Htub.h2tg001157l.g135
Chr13.H5 Htub.h1tg001494l.g462
Chr13.H6 Htub.h1tg001517l.g234
Chr14.H1 Htub.h1tg002779l.g128
Chr14.H2 Htub.h1tg000903l.g284
Chr14.H3 Htub.h1tg000559l.g342
Chr14.H4 Htub.h1tg002179l.g114
Chr14.H5 Htub.h2tg001975l.g125
Chr14.H6 Htub.h1tg002321l.g508
5′
3′
100bp
C
1-FEHI
True gene
Chr04.H3 Htub.h1tg002659l.g270*
Chr04.H5 Htub.h1tg000592l.g407
Chr04.H6 Htub.h2tg000943l.g424
Chr04.H1 Htub.h1tg003296l.g162
1-FEHI
Pseudogene
Chr04.H2 Htub.h1tg001868l.g40
Chr04.H4 Htub.h1tg000593l.g527
frameshift
5′
3′
100bp

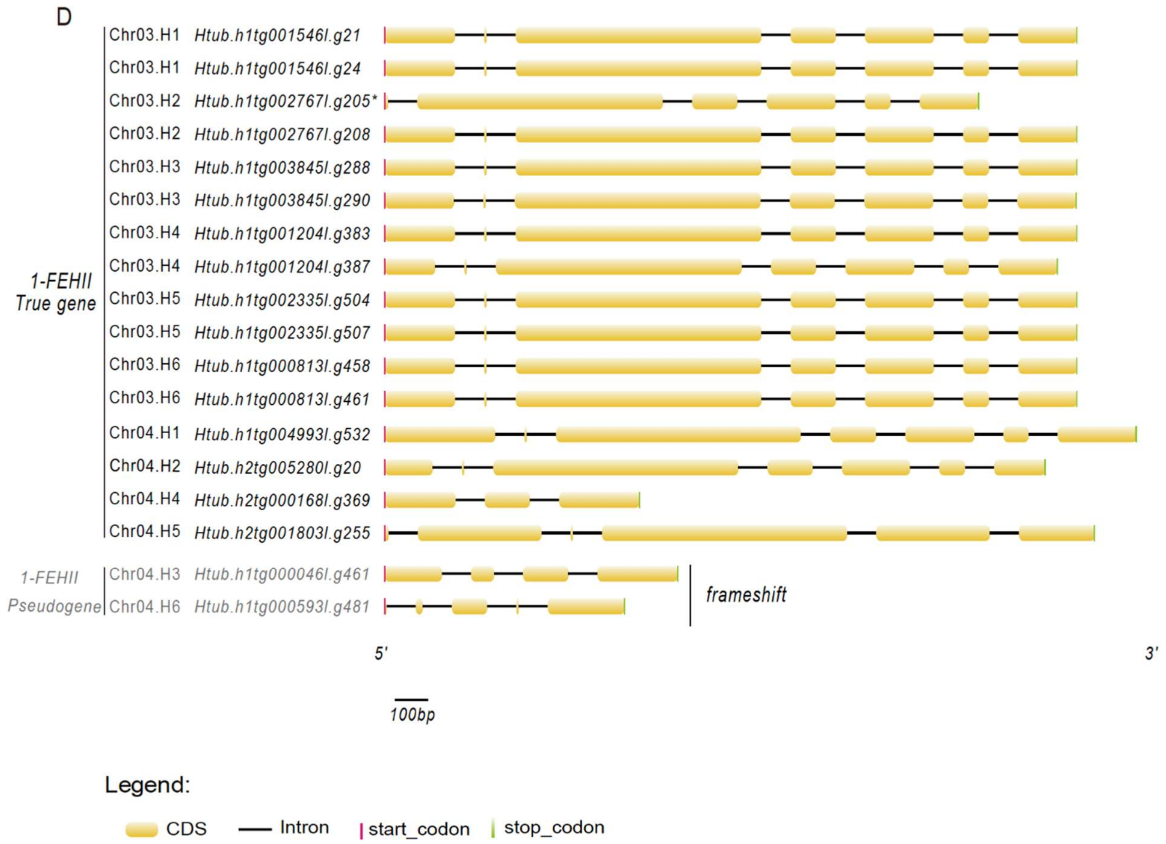

Supplemental Figure 16 Exon-intron structures of inulin metabolism genes of *H. tuberosus* (*Htub*). A Structure diagrams of 12 Htub *1-SST* genes. **B** Structure diagrams of 12 Htub *1-FFT* genes. **C** Structure diagrams of 6 Htub *1-FEHI* genes. **D** Structure diagrams of 18 Htub *1-FEHII* genes. True genes (in black color) and pseudogenes (in gray color) and their locations are given to the left of their structures, and asterisks indicate previously-cloned genes. Most pseudogenes have internal frameshift mutations or stop codons. The structural diagrams were drawn by the online tool of GSDS2.0 (http://gsds.gao-lab.org/).

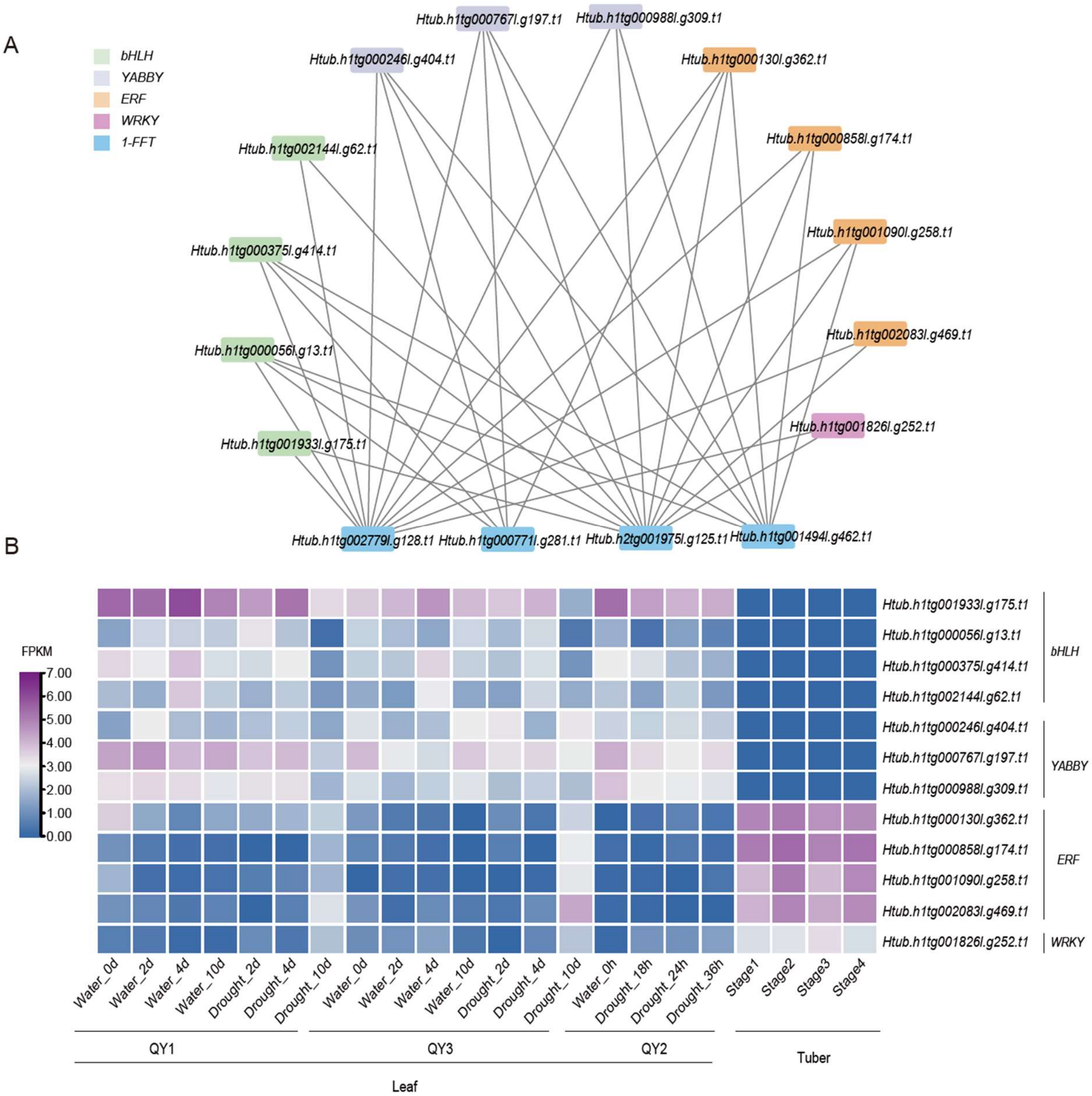

Supplemental Figure 17 Identification of potential transcription factors regulating inulin metabolism genes in hexaploid *H. tuberosus* (*Htub*). **A** Co-expression network of *1-FFT* genes and transcription factors of *bHLH*, *YABBY*, *ERF* and *WRKY* families (labeled in different colors), generated by the weighted gene co-expression network analysis (WGCNA) of transcriptomic profile of various tuber and leaf tissues. The prediction of transcription factors was done by the online tool of PlantTFDB (http://planttfdb.gao-lab.org/prediction.php). Line widths are proportional to the expression correlations among transcription factors and inulin metabolism genes. **B** Expression heatmap of transcription factor genes in tuber tissues of various growth stages and leaf tissues under various water or drought conditions. The color of each square is proportional to the gene expression level of Log2-transformed FPKM (fragments per kilo-base per million mapped fragments). The heatmap was drawn by TBtools.

Supplemental Table 1 Genome sequencing data of *H. tuberosus*

Sequencing technology	Number of reads	Number of bases	SRA accession
PacBio CCS (HiFi)	2,379,809	29,436,118,474	SRR23004845
PacBio CCS (HiFi)	2,242,765	28,025,076,097	SRR23004846
PacBio CCS (HiFi)	2,152,312	26,839,037,330	SRR23004849
PacBio CCS (HiFi)	1,648,530	26,268,319,594	SRR23004868
PacBio CCS (HiFi)	629,108	12,674,253,674	SRR23004869
PacBio CCS (HiFi)	924,872	19,035,337,903	SRR23004870
PacBio CCS (HiFi)	1,414,664	24,402,993,344	SRR23004871
PacBio CCS (HiFi)	1,403,694	24,104,012,019	SRR23004872
PacBio CCS (HiFi)	2,519,881	32,152,513,098	SRR23004859
PacBio CCS (HiFi)	850,161	17,515,001,358	SRR23004841
PacBio CCS (HiFi)	785,017	17,223,039,643	SRR23004842
PacBio CCS (HiFi)	2,537,996	31,674,481,367	SRR23004843
PacBio CCS (HiFi)	2,265,031	28,337,743,748	SRR23004844
Hi-C with PE150	2,065,696,945	302,811,263,849	SRR23023708<br>SRR23023709
WGS with PE150	2,091,999,391	312,922,868,769	SRR23023710<br>SRR23023711

Supplemental Table 2 Statistics of hexaploid genome assembly for *H. tuberosus*

Indicator	Length (bp)		Number	
	Contig	Scaffold	Contig	Scaffold
**Total**	21,591,932,544	21,592,521,744	15,667	9,943
**N50**	6,274,117	200,619,794	1,033	49
**N60**	4,909,041	197,668,618	1,420	60
**N70**	3,716,789	189,594,085	1,926	71
**N80**	2,507,823	178,404,404	2,632	82
**N90**	1,313,541	149,347,181	3,801	96

Supplemental Table 3 Statistics of Hi-C reads mapping to assembled contigs of *H. tuberosus*

Type of read pairs	Number of read pairs	Percent of read pairs (%)
**Total pairs processed**	2,126,126,542	100.00%
**Unmapped pairs**	124,683,526	5.86%
**Low qual pairs**	0	0.00%
**Pairs with singleton**	609,867,551	28.68%
**Unique paired alignments**	298,597,183	14.04%
**Valid interaction pairs**	1,087,708,554	51.16%
**Dangling end pairs**	286,802,404	13.49%
**Re-ligation pairs**	14,261,653	0.67%
**Self-cycle pairs**	2,419,855	0.11%
**Filtered pairs**	0	0.00%
**Dumped pairs**	382,999	0.02%
**Valid interaction**	1,087,708,554	51.16%

Supplemental Table 4 Statistics of repeat annotation of the hexaploid genome of *H. tuberosus*

Repeat	Length (bp)	N50 size (bp)	Percent of genome
**Total TRs**	805,119,755	727	3.72%
**Intact TEs**	4,325,757,383	10,161	20.01%
**Homology TEs**	15,651,896,967	3,426	72.39%
***De novo* TEs**	186,282,389	502	0.86%
**Total TEs**	20,072,276,420	3,626	92.6%

Note: TR is short for tandem repeat, and TE is short for transposon element. Intact TEs were predicted by EDTA based on structural characters of TE, homology TEs were identified by RepeatMasker based on sequence similarity to intact TEs and known TEs in RepBase and TEProtein, and *de novo* TEs were identified by RepeatMasker and RepeatModeler based on sequency copy number. Total TEs refers to all the identified intact TEs, homology TEs and *de novo* TEs.

Supplemental Table 5 Statistics of major types of transposable elements (TEs) in the hexaploid genome of *H. tuberosus*

TE class / order	TE superfamily	Total length (bp)	Percent of genome
**Class I / LTR**		16,578,235,320	76.7%
	Gypsy	9,884,031,229	45.7%
	Copia	2,609,779,936	12.1%
	Unknown	4,016,590,793	18.6%
**Class II / DNA**		3,285,129,515	15.2%
	Mutator	880,430,301	4.1%
	CMC-EnSpm	431,578,160	2.0%
	hAT	467,548,754	2.1%
	PIF-Harbinger	119,950,084	0.6%
**Class II / MITE**		171,258,346	0.8%
**Class II / Helitron**		1,362,401,153	6.3%

Note: LTR is short for long-terminal-repeat retrotransposons, DNA refers to DNA transposon, and MITE is short for minor inverted terminal repeats of transposons.

Supplemental Table 6 Statistics of full-length transcript alignments to the hexaploid genome of *H. tuberosus*

Tissue	Type of transcripts	Number	Percent
Leaf, stem, rhizome and root of seedling stage	Transcripts sequenced by Iso-Seq	1,163,450	100.00%
	Isoseq3 full-length transcripts	938,158	80.64%
	Isoseq3 non-redundant transcripts	96,168	8.27%
	Transcripts aligned to genome	95,793	8.23%
	Alignment identity and coverage >= 95%	94,366	8.11%
Flower of anthesis stage	Transcripts sequenced by Iso-Seq	228,527	100.00%
	Isoseq3 full-length transcripts	203,585	89.09%
	Isoseq3 non-redundant transcripts	23,956	10.48%
	Transcripts aligned to genome	23,839	10.43%
	Alignment identity and coverage >= 95%	23,713	10.38%
Tuber of anthesis stage	Transcripts sequenced by Iso-Seq	293,425	100.00%
	Isoseq3 full-length transcripts	268,761	91.59%
	Isoseq3 non-redundant transcripts	28,338	9.66%
	Transcripts aligned to genome	28,205	9.61%
	Alignment identity and coverage >= 95%	27,983	9.54%

Note: Iso-Seq is the sequencing technology developed by PacBio to sequence full-length transcripts, and IsoSeq3 is the software package developed by PacBio to process the sequencing data of Iso-Seq and generate full-length and non-redundant transcripts. GMAP and filterPSL.pl (an Augustus script, with parameters '–minId=95 –minCover=95') were used to align full-length non-redundant transcripts to genome, and blat2hints.pl (another Augustus script) was used to convert the transcript alignments to the hints file used for gene prediction.

Supplemental Table 7 Statistics of RNA-seq mappings to hexaploid genome of *H. tuberosus* used for gene prediction

Sample	Reads number	Mapping rate	Filtered mapping rate	Number of transcripts
Leaf of QY2 under 18-h drought	18,964,838	96.56%	95.87%	1,100,413
Leaf of QY2 under 24-h drought	18,741,970	96.84%	96.09%	1,087,824
Leaf of QY2 under 36-h drought	18,664,018	96.59%	95.80%	1,090,893
Leaf of QY2 under 10-d drought	16,094,600	97.89%	96.55%	954,465
Leaf of QY2 under 17-d drought	16,690,913	97.68%	96.46%	974,619
Leaf of QY2 under 21-d drought	21,301,522	98.01%	96.72%	1,031,332
Leaf of cultivar Dafeng	12,512,860	99.08%	97.68%	874,881
Leaf of QY1 under 10-d drought	23,917,571	97.33%	96.17%	1,043,540
Leaf of QY1 under 2-d drought	16,644,740	97.13%	96.17%	916,030
Leaf of QY1 under 4-d drought	17,019,482	96.93%	96.17%	953,833
Leaf of QY3 under 10-d drought	20,726,581	99.00%	98.32%	1,155,274
Leaf of QY3 under 2-d drought	17,505,903	98.53%	98.33%	1,058,212
Leaf of QY3 under 4-d drought	17,239,828	99.12%	98.25%	1,079,591
Leaf of cultivar Hindung	10,853,510	98.33%	97.06%	830,384
Leaf of purple cultivar	12,676,263	97.87%	96.73%	785,442
Leaf of QY1 under 0-d water	18,143,784	97.95%	97.17%	975,089
Leaf of QY3 under 0-d water	19,279,152	98.47%	98.22%	1,168,601
Tuber of stolon formation stage	49,640,277	99.35%	98.01%	1,601,600
Tuber of formation stage	48,135,217	99.33%	97.81%	1,497,842
Tuber of rapid expansion stage	47,295,095	99.37%	97.94%	1,426,522
Tuber of maturity stage	42,729,427	99.38%	97.84%	1,392,088
Tuber of cultivar Dafeng	17,099,864	99.11%	98.14%	660,893
Tuber of cultivar Hindung	10,853,510	98.24%	96.99%	644,878
Tuber of stolon formation stage	27,594,202	96.75%	98.26%	1,299,699
Tuber of formation stage	27,480,314	96.59%	98.19%	1,312,324
Tuber of rapid expansion stage	25,279,601	96.66%	98.16%	1,184,444
Tuber of maturity stage	26,982,668	96.70%	98.18%	1,224,213
Tuber of purple cultivar	12,676,263	98.31%	97.16%	552,997

Note: QY1, QY2 and QY3 are cultivars of H. tuberosus. Reads mapping was done using HISAT2, and the alignments were filtered using the Augustus script FilterBam with parameters "--minId=95 --minCover=95". The transcripts of each sample were assembled separately, and merged to remove redundance using StringTie, and then converted into hints file used by Augustus gene prediction.

Supplemental Table 8 Number of predicted genes in the hexaploid genome of *H. tuberosus* by homologous proteins of 10 Asteraceae species

Species used for homology prediction	Number of predicted genes
Arctium lappa	130,305
Artemisia annua	126,828
Artemisia argyi	193,166
Cichorium endivia	184,181
Cichorium intybus	150,658
Erigeron canadensis	89,610
Glebionis coronaria	140,850
Helianthus annuus	214,294
Scalesia atractyloides	176,581
Smallanthus sonchifolius	288,068

Note: the protein sequences of the above 10 species were mapped to the hexaploid genome of *H. tuberosus* using Miniport. For each query sequence, no more than 12 highest-scored target predictions with alignment identities higher than 70% were retained, and converted to hints file used by Augustus gene prediction.

Supplemental Table 9 Statistics of protein-coding genes in the hexaploid genome of *H. tuberosus*

Gene feature	Value
**Number of protein-coding genes**	388,053
**Average CDS length per gene (bp)**	1,151
**Average exon number per gene (bp)**	4.4
**Number of genes with hints**	238,070
**Percent of genes with supporting hints (%)**	61.35%
**BUSCO results of eudicots_odb10**	C:98.4%[S:1.6%,D:96.8%],F:0.4%,M:1.2%,n:2326

Note: The predicted genes were generated by Augustus on the TE (> 80 bp) soft-masked genome with the hints file of transcript and homology protein alignments. In the BUSCO results, C means complete, S means complete single copy, D means complete duplicate, F means fragmental, and M means missing genes of the 2,326 BUSCO genes of eudicots_odb10 database.

Supplemental Table 10 Statistics of function annotation to protein-coding genes of hexaploid *H. tuberosus*

Functional annotation	Number of genes	Percent of genes (%)
**Protein-coding genes**	388,053	100.00%
**Genes with NCBI-NR hits**	349,287	90.01%
**Genes with KEGG hits**	244,435	62.99%
**Genes with InterProScan hits**	349,519	90.07%
**Genes with InterPro terms**	278,506	71.77%
**Genes with GO terms**	203,495	52.44%
**Genes with function terms**	365,080	94.00%

Note: the protein-coding genes were searched against NCBI-NR, KEGG databases using Diamond and InterPro databases using InterProScan to get the functional assignments. Genes with functional terms refer to the genes with at least one hit in NCBI-NR, KEGG, InterPro, or GO database.

Supplemental Table 11 Statistics of each ploid of *H. tuberosus*

	Length (bp)	TE-ratio	Gene-number	tRNA-number	BUSCO	QV	LAI	Reads-mapping%
**Haplotype H1**	3,556,733,948	93.68%	62,787	1,831	89.40%	52.27	18.22	98.17%
**Haplotype H2**	3,292,837,489	96.02%	60,027	1,812	86.80%	52.16	17.96	98.00%
**Haplotype H3**	3,438,070,018	93.77%	61,449	1,907	90.90%	52.27	17.64	98.14%
**Haplotype H4**	3,191,634,373	91.32%	57,873	1,754	83.80%	51.92	17.68	97.82%
**Haplotype H5**	3,484,266,179	91.50%	61,707	1,689	88.50%	52.46	17.59	98.11%
**Haplotype H6**	3,267,864,681	91.71%	56,412	1,330	85.90%	52.50	17.2	97.86%

Supplemental Table 12 Feature statistics of 102 pseudochromosomes of hexaploid *H. tuberosus*

Chr	Length (bp)	Contig number	TE-ratio	Gene number	tRNA number	QV	LAI
Chr01.H1	212,055,481	64	93.26%	3,647	112	51.95	18.87
Chr01.H2	172,772,467	58	92.25%	3,214	126	52.71	18.58
Chr01.H3	207,103,226	61	93.11%	3,716	135	52.07	18.53
Chr01.H4	194,780,507	47	92.62%	3,514	120	51.43	17.45
Chr01.H5	197,668,618	60	93.16%	3,538	116	53.26	18.37
Chr01.H6	189,594,085	48	92.81%	3,212	84	51.80	17.61
Chr02.H1	198,003,350	61	93.76%	3,198	116	52.28	17.52
Chr02.H2	186,553,275	50	93.53%	2,979	74	51.45	18.44
Chr02.H3	183,357,911	52	93.57%	2,918	90	51.97	17.03
Chr02.H4	130,132,382	44	92.42%	2,374	78	51.41	17.53
Chr02.H5	200,270,673	72	93.70%	3,129	73	51.57	17.79
Chr02.H6	194,280,497	58	93.78%	3,021	55	52.08	18.2
Chr03.H1	223,930,843	58	93.21%	3,909	130	51.62	17.89
Chr03.H2	217,360,341	48	92.85%	3,975	132	52.26	17.5
Chr03.H3	214,316,459	74	93.09%	3,663	110	52.57	16.87

Chr	Length (bp)	Contig number	TE-ratio	Gene number	tRNA number	QV	LAI
Chr03.H4	217,148,793	69	93.17%	3,838	128	51.46	18.22
Chr03.H5	230,013,764	41	93.25%	4,109	118	52.65	17.68
Chr03.H6	205,661,489	54	92.92%	3,799	94	51.87	18.18
Chr04.H1	234,777,701	235	92.92%	4,273	125	52.38	18.06
Chr04.H2	249,351,921	77	93.04%	4,534	150	51.96	18.06
Chr04.H3	224,626,285	61	92.87%	4,010	119	51.96	17.5
Chr04.H4	165,760,602	74	93.94%	2,615	80	52.62	17.59
Chr04.H5	254,663,628	55	93.06%	4,539	119	52.05	17.3
Chr04.H6	212,116,677	38	93.22%	3,739	107	52.53	17.38
Chr05.H1	193,142,728	55	94.28%	3,010	102	51.84	18.14
Chr05.H2	175,062,967	52	93.34%	3,078	55	53.08	16.99
Chr05.H3	181,319,132	34	93.38%	3,065	61	52.98	17.31
Chr05.H4	143,565,859	43	93.28%	2,628	95	52.46	18.33
Chr05.H5	186,106,489	40	93.59%	3,058	85	52.82	17.24
Chr05.H6	186,544,835	49	93.65%	3,140	55	52.96	17.55
Chr06.H1	208,927,764	61	92.82%	3,882	101	52.50	16.98
Chr06.H2	199,461,199	64	92.44%	3,917	92	50.97	17.58
Chr06.H3	203,104,519	84	92.78%	3,828	118	52.94	17.17
Chr06.H4	197,693,409	78	92.49%	3,823	86	52.57	18.24
Chr06.H5	180,985,612	62	92.96%	3,491	113	51.68	16.92
Chr06.H6	159,471,504	38	92.19%	3,230	43	52.56	16.34
Chr07.H1	172,084,673	51	94.11%	2,544	55	53.14	17.4
Chr07.H2	167,289,314	54	93.90%	2,795	51	52.37	17.38
Chr07.H3	172,895,877	43	94.04%	2,648	53	52.12	16.72
Chr07.H4	155,454,680	30	93.70%	2,450	63	53.97	16.54
Chr07.H5	165,854,593	42	93.61%	2,637	41	52.58	16.16
Chr07.H6	154,860,823	42	94.10%	2,309	35	52.22	17.65
Chr08.H1	229,366,829	47	93.66%	3,858	115	52.03	17.79
Chr08.H2	218,405,125	57	93.61%	3,671	123	51.97	17.18
Chr08.H3	201,527,393	62	93.11%	3,600	114	53.08	17.87
Chr08.H4	193,321,631	44	93.29%	3,290	106	52.98	17.58
Chr08.H5	209,801,722	68	93.14%	3,672	126	52.25	19.19
Chr08.H6	200,331,118	36	93.06%	3,599	126	53.07	17.77
Chr09.H1	211,635,655	57	92.73%	3,808	115	52.44	17.21
Chr09.H2	225,640,645	66	92.41%	4,336	118	52.69	17.01
Chr09.H3	223,868,824	86	92.60%	4,086	109	51.76	17.4
Chr09.H4	193,634,118	108	92.57%	3,531	133	50.93	18.89
Chr09.H5	192,679,956	47	92.60%	3,533	114	53.93	16.98
Chr09.H6	203,837,971	69	92.38%	3,781	70	53.41	17.66
Chr10.H1	227,521,969	43	93.28%	3,779	129	52.47	18.23

Chr	Length (bp)	Contig number	TE-ratio	Gene number	tRNA number	QV	LAI
Chr10.H2	218,385,818	40	93.29%	3,797	112	51.75	17.85
Chr10.H3	219,070,697	67	92.50%	3,997	141	52.40	17.48
Chr10.H4	197,776,477	49	92.92%	3,443	151	52.50	17.65
Chr10.H5	216,187,059	59	92.80%	3,927	100	52.22	17.84
Chr10.H6	214,957,670	38	93.20%	3,833	102	52.68	17.38
Chr11.H1	212,826,509	50	92.83%	3,777	139	51.54	18.25
Chr11.H2	215,859,532	69	92.89%	3,873	168	51.93	18.24
Chr11.H3	223,483,716	43	93.07%	3,942	163	52.02	18.25
Chr11.H4	212,768,019	52	93.27%	3,449	161	51.20	17.78
Chr11.H5	217,659,014	68	93.03%	3,901	154	51.74	18.19
Chr11.H6	188,249,379	51	92.78%	3,286	102	52.08	17.96
Chr12.H1	200,619,794	97	93.09%	3,657	67	54.01	18.13
Chr12.H2	188,337,866	60	93.20%	3,497	77	54.77	18.61
Chr12.H3	195,318,154	49	92.75%	3,619	113	51.89	17.88
Chr12.H4	178,404,404	59	93.27%	3,230	62	51.81	17.39
Chr12.H5	185,692,016	57	92.90%	3,399	19	54.23	17.23
Chr12.H6	147,719,899	89	93.03%	2,599	27	53.51	17.68
Chr13.H1	223,861,238	50	92.73%	3,989	100	51.90	17.29
Chr13.H2	226,807,695	67	93.10%	3,921	109	52.80	17.25
Chr13.H3	211,514,492	94	92.96%	3,721	117	52.10	17.22
Chr13.H4	190,114,262	48	93.64%	3,156	69	51.85	17.66
Chr13.H5	197,619,903	84	93.08%	3,426	103	52.40	17.7
Chr13.H6	210,898,529	74	92.86%	3,782	67	52.37	17.73
Chr14.H1	204,751,212	56	92.55%	4,044	101	51.95	18.98
Chr14.H2	192,141,033	52	91.87%	3,984	121	53.23	18.68
Chr14.H3	199,215,346	54	92.04%	4,104	124	51.33	18.06
Chr14.H4	199,989,373	44	92.09%	4,072	90	51.34	18.21
Chr14.H5	199,784,625	54	92.47%	3,893	92	52.74	18.58
Chr14.H6	183,708,172	59	92.15%	3,702	117	52.28	18.45
Chr15.H1	165,933,527	59	92.92%	2,921	100	52.25	17.69
Chr15.H2	99,095,262	30	91.05%	1,998	69	50.90	17.56
Chr15.H3	143,810,945	35	92.48%	2,818	92	53.01	18.84
Chr15.H4	151,017,016	47	92.70%	2,743	85	51.19	18.21
Chr15.H5	164,986,335	29	92.57%	3,079	68	52.50	18.82
Chr15.H6	149,347,181	40	93.04%	2,753	72	53.20	17.97
Chr16.H1	228,876,035	43	92.75%	4,164	151	52.89	16.71
Chr16.H2	246,409,973	56	93.01%	4,369	151	51.60	16.9
Chr16.H3	230,091,574	101	92.49%	4,461	162	52.37	17.68
Chr16.H4	213,951,254	54	92.32%	4,120	182	53.08	17.27
Chr16.H5	213,590,533	63	92.59%	3,886	178	51.97	16.71

Chr	Length (bp)	Contig number	TE-ratio	Gene number	tRNA number	QV	LAI
Chr16.H6	205,270,347	83	93.14%	3,419	94	53.04	18.05
Chr17.H1	228,292,981	55	93.08%	4,123	73	52.18	18.46
Chr17.H2	203,117,482	60	93.30%	3,454	84	51.51	17.25
Chr17.H3	235,497,988	56	92.84%	4,215	86	52.76	18.04
Chr17.H4	198,827,422	58	93.32%	3,477	65	51.40	16.83
Chr17.H5	213,812,042	66	92.79%	3,824	70	52.61	18
Chr17.H6	215,321,836	55	92.96%	3,871	80	51.75	18.57

Supplemental Table 13 Statistics of predicted non-coding RNA genes in hexaploid *H. tuberosus*

Type of non-coding genes	Number of non-coding genes
**5S rRNA genes**	27,985
**18S rRNA genes**	4,652
**28S rRNA genes**	4,708
**Met tRNA genes**	810
**Asp tRNA genes**	624
**Asn tRNA genes**	462
**Ser tRNA genes**	600
**Thr tRNA genes**	488
**Ala tRNA genes**	410
**Ile tRNA genes**	1,319
**His tRNA genes**	301
**Glu tRNA genes**	516
**Gly tRNA genes**	827
**Val tRNA genes**	647
**Arg tRNA genes**	711
**Leu tRNA genes**	1,216
**Lys tRNA genes**	495
**Pro tRNA genes**	538
**Gln tRNA genes**	400
**Phe tRNA genes**	425
**Cys tRNA genes**	216
**Trp tRNA genes**	181
**Tyr tRNA genes**	229

Note: rRNA and tRNA genes were predicted by tRNAScan-SE and RNAmmer, respectively.

Supplemental Table 14 Summary of public genome data used in this study

Species Name	Ploidy	Sequencing technology	Genome Size (G)	Assembled size (G)	Contig N50 size	Data version
Arctium lappa	2n = 36	Pacbio+HiC	1.72	1.72	74.7Mb	ASM2352574v1

Artemisia annua	2n = 18	Illumina+Roche454+PacBio	1.76	1.74	18.9 Kb	ASM311234v1
Artemisia argyi	2n = 34	Pacbio+Illumina+HiC	8.00	8	6.3Mb	ASM3068699v1
Cichorium endivia	2n = 18	Pacbio+HiC	0.89	0.89	9.0Mb	v1
Cichorium intybus	2n = 18	Pacbio+HiC	1.28	1.28	15.32Mb	ASM2352571v1
Coffea canephora	2n = 22	Roche 454+Sanger+genetic map	0.71	0.57	49.4 Kb	AUK_PRJEB4211_v1
Cynara cardunculus	2n = 34	illumina+genetic map+HiC	1.07	0.73	17 Kb	CcrdV1
Erigeron canadensis	2n = 18	Pacbio+HiC	0.47	0.43	1.68 Mb	C_canadensis_v1
Glebionis coronaria	2n = 18	Pacbio+HiC	6.80	6.8	3.87Mb	Gcor_v1
Helianthus annuus	2n = 34	Pacbio+genetic map+phyical map	3.60	3	399 Kb	HanXRQr2.0-SUNRISE
Lactuca sativa	2n = 18	illumina+HiC+genetic map	2.70	2.38	36 Kb	Lsat_Salinas_v7
Mikania micrantha	2n = 38	Pacbio+HiC	1.86	1.79	1,352 Kb	ASM936387v1
Stevia rebaudiana	2n = 22	Pacbio+HiC	1.16	1.41	616 Kb	14169491.v1
Scalesia atractyloides	2n = 68	Pacbio+Dovetail	3.20	3.20	5.8Mb	scalesia_atractyloides_v1
Smallanthus sonchifolius	2n = 58	Pacbio+HiC	2.70	2.70	66.5Mb	ASM2352597v1

243

Supplemental Table 15 Statistics of RNA-seq reads mappings to hexaploid *H. tuberosus*

Sample description	Sequence Read Archive accession	Reads Number	Alignment Rate
**QY2 Water 0h replicate 1**	SRR12998554	21,430,315	99.93%
**QY2 Water 0h replicate2**	SRR12998551	21,801,397	99.91%
**QY2 Water 0h replicate 3**	SRR12998550	18,881,845	99.93%
**QY2 Drought 18h replicate 1**	SRR12998553	18,964,838	99.86%
**QY2 Drought 18h replicate2**	SRR12998552	18,227,343	99.83%
**QY2 Drought 18h replicate 3**	SRR12998549	18,642,298	99.87%
**QY2 Drought 24h replicate1**	SRR12998560	18,741,970	99.92%
**QY2 Drought 24h replicate 2**	SRR12998559	18,776,288	99.93%
**QY2 Drought 24h replicate 3**	SRR12998558	18,607,205	99.93%
**QY2 Drought 36h replicate1**	SRR12998557	18,664,018	99.94%
**QY2 Drought 36h replicate2**	SRR12998556	18,773,479	99.94%
**QY2 Drought 36h replicate3**	SRR12998555	18,940,141	99.80%
**QY2 Water 10dreplicate1**	SRR12978321	18,461,729	99.25%
**QY2 Water 10dreplicate2**	SRR12978320	19,444,150	99.07%
**QY2 Water 10dreplicate3**	SRR12978311	19,160,703	99.77%
**QY2 Water 17dreplicate1**	SRR12978310	20,483,109	99.88%
**QY2 Water 17d replicate2**	SRR12978309	18,284,530	99.90%
**QY2 Water 17d replicate3**	SRR12978308	25,801,358	99.80%
**QY2 Water 21d replicate1**	SRR12978307	17,002,173	99.22%
**QY2 Water 21d replicate 2**	SRR12978306	18,443,789	99.33%
**QY2 Water 21d replicate 3**	SRR12978305	21,060,102	99.36%
**QY2 Drought 10d replicate 1**	SRR12978304	16,094,600	99.81%
**QY2 Drought 10d replicate 2**	SRR12978319	15,559,227	99.72%
**QY2 Drought 10d replicate 3**	SRR12978318	15,128,277	99.82%
**QY2 Drought 17d replicate 1**	SRR12978317	16,690,913	99.71%
**QY2 Drought 17d replicate 2**	SRR12978316	20,188,515	99.46%
**QY2 Drought 17d replicate 3**	SRR12978315	16,201,194	99.85%
**QY2 Drought 21d replicate 1**	SRR12978314	21,301,522	99.84%
**QY2 Drought 21d replicate 2**	SRR12978313	22,610,618	99.56%
**QY2 Drought 21d replicate 3**	SRR12978312	23,479,965	99.87%
**QY1 Drought 10d replicate 1**	SRR10338835	23,917,571	99.70%
**QY1 Drought 10d replicate 2**	SRR10338824	19,329,673	99.60%
**QY1 Drought 2d replicate 1**	SRR10338847	16,644,740	99.06%
**QY1 Drought 2d replicate 2**	SRR10338826	18,639,728	98.63%
**QY1 Drought 4d replicate 1**	SRR10338846	17,019,482	98.83%
**QY1 Drought 4d replicate 2**	SRR10338825	17,004,337	99.14%
**QY3 Drought 10d replicate 1**	SRR10338821	20,726,581	99.26%
**QY3 Drought 10d replicate 2**	SRR10338844	17,323,130	99.82%
**QY3 Drought 2d replicate 1**	SRR10338823	17,505,903	98.36%

Sample description	Sequence Read Archive accession	Reads Number	Alignment Rate
**QY3 Drought 2d replicate 2**	SRR10338820	18,361,572	99.36%
**QY3 Drought 4d replicate 1**	SRR10338822	17,239,828	99.40%
**QY3 Drought 4d replicate 2**	SRR10338845	17,142,117	99.44%
**QY1 Water 0d replicate 1**	SRR10338843	18,143,784	99.16%
**QY1 Water 0d replicate 2**	SRR10338842	18,555,542	99.04%
**QY1 Water 10d replicate 1**	SRR10338837	19,896,757	99.23%
**QY1 Water 10d replicate 2**	SRR10338833	19,205,742	99.44%
**QY1 Water 2d replicate 1**	SRR10338839	17,041,557	99.11%
**QY1 Water 2d replicate 2**	SRR10338836	16,875,354	99.06%
**QY1 Water 4d replicate 1**	SRR10338838	16,667,397	99.34%
**QY1 Water 4d replicate 2**	SRR10338834	18,451,113	99.16%
**QY3 Water 0d replicate 1**	SRR10338841	19,279,152	98.33%
**QY3 Water 0d replicate 2**	SRR10338840	19,151,381	99.10%
**QY3 Water 10d replicate 1**	SRR10338830	16,650,398	99.78%
**QY3 Water 10d replicate 2**	SRR10338827	17,471,873	98.44%
**QY3 Water 2d replicate 1**	SRR10338832	16,249,395	99.27%
**QY3 Water 2d replicate 2**	SRR10338829	18,050,890	98.27%
**QY3 Water 4d replicate 1**	SRR10338831	16,124,164	99.13%
**QY3 Water 4d replicate 2**	SRR10338828	18,839,885	98.89%
**Stolon formation stage1 replicate 1**	SRR10586247	27,594,202	99.45%
**Stolon formation stage1 replicate 2**	SRR10586246	36,832,554	99.40%
**Stolon formation stage1 replicate 3**	SRR10586243	21,902,615	99.52%
**Tuber formation stage2 replicate 1**	SRR10586242	27,480,314	99.40%
**Tuber formation stage2 replicate 2**	SRR10586241	23,120,341	99.49%
**Tuber formation stage2 replicate 3**	SRR10586240	24,801,883	99.45%
**Tuber rapid intamescentia period3 replicate 1**	SRR10586239	25,279,601	99.46%
**Tuber rapid intamescentia period3 replicate 2**	SRR10586238	28,377,348	99.47%
**Tuber rapid intamescentia period3 replicate 3**	SRR10586237	30,349,670	99.29%
**Tuber maturity stage4 replicate 1**	SRR10586236	26,982,668	99.46%
**Tuber maturity stage4 replicate 2**	SRR10586245	24,916,267	99.52%
**Tuber maturity stage4 replicate 3**	SRR10586244	21,834,813	99.52%
**Dafeng Leaf**	SRR3161859	12,512,860	99.92%
**Hindung Leaf**	SRR3161862	13,476,361	99.85%
**Purple Leaf**	SRR3161863	12,515,384	99.79%
**Dafeng Tuber**	SRR3161860	17,099,864	99.63%
**Hindung Tuber**	SRR3161861	10,853,510	99.70%
**Purple Tuber**	SRR3161864	12,676,263	99.63%
**Stolon formation stage 1**	SRR23004864	49,640,277	99.99%
**Stolon formation stage 2**	SRR23004863	45,045,585	99.99%
**Stolon formation stage 3**	SRR23004861	41,440,662	99.99%

Sample description	Sequence Read Archive accession	Reads Number	Alignment Rate
**Stolon formation stage 4**	SRR23004860	45,354,586	99.99%
**Tuber formation stage 1**	SRR23004858	48,135,217	99.99%
**Tuber formation stage 2**	SRR23004857	58,656,555	99.99%
**Tuber formation stage 3**	SRR23004856	60,530,226	99.99%
**Tuber formation stage 4**	SRR23004862	44,582,512	99.99%
**Tuber rapid intamescentia period 1**	SRR23004855	47,295,095	99.99%
**Tuber rapid intamescentia period 2**	SRR23004854	47,315,121	99.99%
**Tuber rapid intamescentia period 3**	SRR23004853	54,106,220	99.99%
**Tuber rapid intamescentia period 4**	SRR23004852	53,909,638	99.99%
**Tuber maturity stage1**	SRR23004851	42,729,427	99.99%
**Tuber maturity stage2**	SRR23004850	52,255,109	99.99%
**Tuber maturity stage3**	SRR23004848	40,033,073	99.99%
**Tuber maturity stage4**	SRR23004847	42,472,242	99.99%

Note: Rep is short for replicate. Reads mapping was done using HISAT2 with default settings. Of the above RNA-seq datasets, SRR23004847 to SRR23004864 were generated in this study and the others were public datasets downloaded from NCBI SRA.

Supplemental Table 16 Identification of binding motifs of transcription factors (TFs) in the promoter regions of inulin metabolism genes of hexaploid *H. tuberosus (Htub)*

Pattern name	Sequence name	Family	Start	Stop	P-value	Matched sequence
AT2G20180	Htub.h2tg001975l.g125.t1	bHLH	1132	1145	2.56E-07	GGCGCGCCACGTGT
AT1G69010	Htub.h1tg000559l.g342.t1	bHLH	1671	1685	8.99E-07	CACGTGCAATACCCG
AT3G59060	Htub.h1tg000901l.g450.t1	bHLH	1175	1182	4.87E-06	TCACGTGG
AT1G09530	Htub.h1tg001494l.g462.t1	bHLH	1260	1269	8.09E-06	AGCCATGTGG
AT1G09530	Htub.h1tg000771l.g281.t1	bHLH	1260	1269	8.09E-06	AGCCATGTGG
AT1G22810	Htub.h1tg000771l.g281.t1	ERF	540	554	3.49E-06	TTTGGTGGTGGTGGA
AT5G18450	Htub.h1tg001494l.g462.t1	ERF	538	552	3.44E-06	TTTGGTGGTGGTGGA
AT1G71450	Htub.h1tg002779l.g128.t1	ERF	209	228	2.34E-10	CGCCTCCACTGTCGCC GCCG
AT1G44830	Htub.h1tg000559l.g342.t1	ERF	1513	1527	1.69E-07	CGACAACCACCGCCA
AT1G19210	Htub.h1tg000901l.g450.t1	ERF	986	999	4.53E-06	GGGGCCGGAGGAGA
AT2G04880	Htub.h1tg000559l.g342.t1	WRKY	1328	1338	1.17E-05	TTGACCCACCG
AT2G04880	Htub.h1tg002779l.g128.t1	WRKY	1493	1503	1.17E-05	TTGACCCACCG
AT2G25000	Htub.h1tg001494l.g462.t1	WRKY	105	115	1.85E-05	ACGGTCAAACT
AT4G24240	Htub.h2tg001975l.g125.t1	WRKY	53	66	4.33E-05	TTAGTTGACATTTT

AT2G04880	Htub.h1tg000901l.g450.t1	WRKY	653	663	4.80E-05	TTGTCCGAGCC
AT2G45190	Htub.h1tg000901l.g450.t1	YABBY	1225	1234	5.96E-05	CAATCATGAC

Note: known TFs of *Arabidopsis thaliana* were used as queries to search for their binding motifs in the promoter regions (upstream 2-Kb) of target genes. The analysis was performed using the online tool of PlantRegMap (http://plantregmap.gao-lab.org/binding_site_prediction.php).
